# Recent Advances in Non‐Ti MXenes: Synthesis, Properties, and Novel Applications

**DOI:** 10.1002/advs.202303998

**Published:** 2024-06-18

**Authors:** Karim Khan, Ayesha Khan Tareen, Waqas Ahmad, Iftikhar Hussain, Mujeeb U. Chaudhry, Asif Mahmood, Muhammad Farooq Khan, Han Zhang, Zhongjian Xie

**Affiliations:** ^1^ School of Electrical Engineering and Intelligentization Dongguan University of Technology Dongguan 523808 China; ^2^ Shenzhen Nuoan Environmental and Safety Inc. Shenzhen 518107 China; ^3^ Additive Manufacturing Institute Shenzhen University Shenzhen 518060 China; ^4^ Shenzhen Engineering Laboratory of Phosphorene and Optoelectronics International Collaborative Laboratory of 2D Materials for Optoelectronics Science and Technology of Ministry of Education Institute of Microscale Optoelectronics Engineering Shenzhen University Shenzhen 518060 China; ^5^ School of Mechanical Engineering Dongguan University of Technology Dongguan 523808 China; ^6^ Institute of Fundamental and Frontier Sciences University of Electronic Science and Technology of China Chengdu 610054 China; ^7^ Department of Mechanical Engineering City University of Hong Kong 83 Tat Chee Avenue Kowloon 999077 Hong Kong; ^8^ A. J. Drexel Nanomaterials Institute and Department of Materials Science and Engineering Drexel University Philadelphia PA 19104 USA; ^9^ Department of Engineering Durham University Lower Mountjoy South Rd Durham DH1 3LE UK; ^10^ School of Chemical and Biomolecular Engineering The University of Sydney Sydney 2006 Australia; ^11^ Department of Electrical Engineering Sejong University Seoul 05006 Republic of Korea; ^12^ Shenzhen Children's Hospital Clinical Medical College of Southern University of Science and Technology Shenzhen Guangdong 518038 P. R. China

**Keywords:** non‐Ti MXenes, properties of non‐Ti MXenes, M‐based MXenes, synthesis of non‐Ti MXenes, 2D nanomaterials

## Abstract

One of the most fascinating 2D nanomaterials (NMs) ever found is various members of MXene family. Among them, the titanium‐based MXenes, with more than 70% of publication‐related investigations, are comparatively well studied, producing fundamental foundation for the 2D MXene family members with flexible properties, familiar with a variety of advanced novel technological applications. Nonetheless, there are still more candidates among transitional metals (TMs) that can function as MXene NMs in ways that go well beyond those that are now recognized. Systematized details of the preparations, characteristics, limitations, significant discoveries, and uses of the novel M‐based MXenes (M‐MXenes), where M stands for non‐Ti TMs (M = Sc, V, Cr, Y, Zr, Nb, Mo, Hf, Ta, W, and Lu), are given. The exceptional qualities of the 2D non‐Ti MXene outperform standard Ti‐MXene in several applications. There is many advancement in top‐down as well as bottom‐up production of MXenes family members, which allows for exact control of the M‐characteristics MXene NMs to contain cutting‐edge applications. This study offers a systematic evaluation of existing research, covering everything in producing complex M‐MXenes from primary limitations to the characterization and selection of their applications in accordance with their novel features. The development of double metal combinations, extension of additional metal candidates beyond group‐(III–VI)B family, and subsequent development of the 2D TM carbide/TMs nitride/TM carbonitrides to 2D metal boride family are also included in this overview. The possibilities and further recommendations for the way of non‐Ti MXene NMs are in the synthesis of NMs will discuss in detail in this critical evaluation.

## Graphical abstract

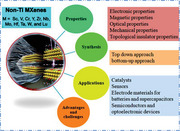

## 1 Introduction: MXene in Brief

Generally, depending on dimensionality, a material has a variety of qualities.^[^ ^1^ ^]^ Recently, 2D materials have received numerous scientific study research interests. The 2D materials have distinctive features that set them distinct from their bulk counterparts.^[^ ^2^, ^3^, ^4^, ^5^, ^6^, ^7^, ^8^, ^9^, ^10^, ^11^ ^]^ The 2D materials with high aspect ratio structure, extended lateral dimension, and an atomic/molecular thickness have been intensively explored in the materials research community in recent era. Graphene, the first invented 2D nanomaterial (NM), has demonstrated a broad range of possible novel devices applications in advanced nanotechnological world. Since the discovery of Nobel Prize awarded material, graphene (2004), a range of other novel 2D NMs, for example, MXenes, transition metal dichalcogenides (TMDs), Xenes, and 3D structure of 2D materials as metal organic frameworks (MOFs), etc., were widely discovered as functional materials for energy producing/storage, environmental protection, sensing, biomedical, catalysis, etc., applications. By now 2D materials family has over 150 members, with hundreds more expected to join by modifying transition metal (TM) components ranging from metals, semimetals, semiconductors, and insulators.^[^ ^2^, ^3^, ^4^, ^8^, ^12^, ^13^, ^14^, ^15^, ^16^, ^17^, ^18^, ^19^, ^20^, ^21^, ^22^, ^23^, ^24^, ^25^, ^26^, ^27^, ^28^, ^29^, ^30^, ^31^ ^]^

**Figure 1:**
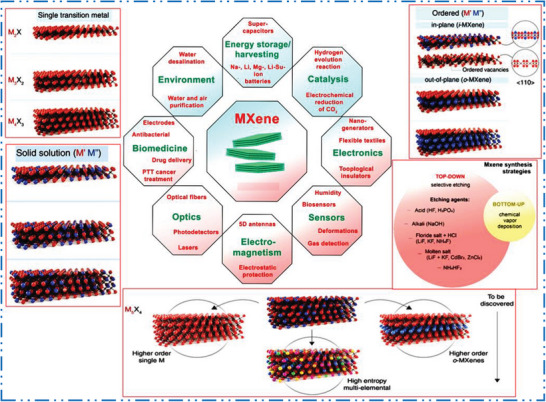
Scheme shows MXenes structures, synthesis methods, and applications. Reproduced with permission.^[^ ^34^ ^]^ Copyright 2021, American Association for the Advancement of Science (AAAS).

For the first time, 2D titanium carbide (Ti_3_C_2_) was accidently invented in 2011 by selective etching of Al layer of Ti_3_AlC_2_ precursor via employing acid, like, HF, called a first member of 2D MXene NMs family.^[^ ^32^ ^]^ Later on, further members of 2D MXenes family were discovered and now consists of all 2D transition metals (TMs) carbides (TMCs), TM nitrides (TMNs), and TM carbonitrides. Now this family consists of more than 50 members, who are experimentally investigated and the discovery of further more than 100 MXene NMs members is theoretically studied, causes it as a future biggest family of 2D materials, till date. M*_n_*_+1_X*_n_* T*_x_* (*n* = 1 to 4) is a general formula for MXene‐based NMs, where M indicate early TMs (e.g., Sc, Ti, V, Cr, Hf, Zr, Nb, Mo, Ta, and so on), and X shows carbon or nitrogen,^[^ ^33^ ^]^ and T*_x_* (where *x* varies) denotes single or mixed termination groups (e.g., T = F, O, NH, S, OH, Cl, Br, Te, and Se). Mono‐M elements (like, Ti_2_C, Nb_4_C_3_, etc.), solid solutions at least from two different “M” elements (such as (Ti,V)_3_C_2_ and (Cr,V)_3_C_2_), and ordered double‐“M” elements (where one TM occupies the perimeter layers while another fills a central “M” layer) are all possible (e.g., Mo_2_TiC_2_, Mo_2_Ti_2_C_3_, etc., where the outer “M” layers are “Mo” and the central “M” layers are “Ti”). Carbonitrides are formed by solid solutions on “X” site (**Figure** **1**). Regarding to applications, 2D MXene NMs have raised to prominence as a next‐generation NMs for the investigation of ecologically acceptable catalysis for energy storage devices, optoelectronics, electromagnetic interference shielding, biomedicine, sensors environmental technology solutions, etc., thanks to their fascinating electrical and structural features (Figure 1).

In contrast to other 2D NMs mostly MXene NMs are not commonly bonded via van der Waals (vdWs) forces in their precursors. The link between MXene layers is really given by the monoatomic group 13 and 14 elements, which make up most of MAX A‐layer atoms (e.g., Si, Al, and Ga). Elemental layer from MAX precursors can be removed to produce various MXene NMs. Various chemical reactivates in metallic bonds (M–A) and mixed metallic/ionic/covalent bonds (M–X) in the MAX‐based precursors make this technique theoretically feasible.^[^ ^35^ ^]^ MXenes have hydrophilic surfaces termination with hydroxyl, oxygen, and fluorine groups. They can be turned into films, devices, and coatings because of easy processing and least required stabilization.

**Figure 2:**
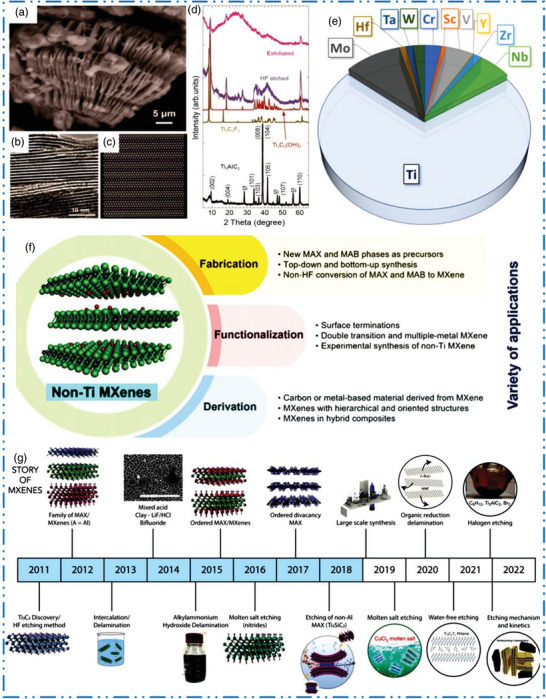
a,b) SEM, along with TEM of ML Ti_3_C_2_T*_x_* etched by using HF, c) atomic model of Ti_3_C_2_T*_x_* after Li insertion (Ti_3_C_2_Li_2_), d) XRD of Ti_3_AlC_2_, computational‐XRD of Ti_3_C_2_F_2_ as well as Ti_3_C_2_(OH)_2_, and Ti_3_C_2_T*_x_* gained by etching/exfoliation. Reproduced with permission.^[^ ^42^ ^]^ Copyright 2022, Wiley. e) Chart shows the comparison of Ti‐based and non‐Ti MXene. Reproduced with permission.^[^ ^37^ ^]^ Copyright 2023, Elsevier. f) Summary of article focuses areas and future directions of non‐Ti MXene NMs. g) Story of MXenes family production. Reproduced with permission.^[^ ^34^ ^]^ Copyright 2021, American Association for the Advancement of Science (AAAS).

As first Ti_3_C_2_T*_x_* MXene was experimentally synthesized in 2011 by Al layers etching of Ti_3_AlC_2_ MAX in HF‐acid.^[^ ^32^ ^]^ The resulting Ti_3_C_2_T*_x_* exhibited an accordion‐like composition with several layers linked together by hydrogen and vdWs bonds (**Figure** **2**a–c).^[^ ^36^ ^]^ The 2D Ti_3_C_2_T*_x_* nanosheets (NSs) maintain the same stoichiometry of titanium (Ti) as well as carbon (C) as in bulk Ti_3_AlC_2_ precursor while having a different shape, with surface‐bound functional O, OH, and F groups (Figure 2d). Reactivity of each MAX precursor differs depends on various bonding strengths in M–A and M–X, it should be noted that the etching method of the 2D Ti_3_C_2_T*_x_* NSs cannot be used as a generic method for various 2D MXene NMs.^[^ ^34^ ^]^ Figure 2e shows the comparison of Ti‐ and non‐Ti based MXene NMs, which clear that mostly studied 2D MXene NMs are based on Ti‐MXene NMs.^[^ ^37^ ^]^ MXene NMs’ crystals have closed packed M atoms, while X atoms reside in octahedral interstitial positions, resulting in hexagonal closed packed configuration with *P63/mmc* space group symmetry.^[^ ^38^ ^]^ Ti_3_C_2_ investigation laid the groundwork for the production of Ti_2_C, Ta_4_C_3_, and additional 2D MXene NMs from their MAX‐based predecessors, exhibiting three special kinds of potential configurations (such as M_2_X, M_3_X_2_, and M_4_X_3_). M_5_C_4_ was afterward introduced; boosting structural variety and getting theoretically achievable compositions are over 100, including in‐plane MXene (i‐MXenes) as well as out‐of‐plane TM atom ordered MXene (O‐MXenes). When different T*_x_* on MXene NMs surface are considered, the figure of special compositions developed by order of magnitude. MXene NMs' capacity to create carbonitrides and solid solutions implies a theoretically infinite quantity of compositions and leads in a novel period of theoretically determined atomistic formation of the 2D NMs.

Most members of 2D materials are dielectrics, semiconductors, or semimetals in nature but MXene‐based NMs are mostly metallic conductors because TMs can give free electrons and function as carriers^[^ ^39^ ^]^ (e.g., phosphorene and molybdenum disulfide). The 2D MXene NMs, especially Ti_3_C_2_T*_x_* with few defects can achieve a highest conductivity of 20 000 S cm^−1^. Furthermore, abundance of surface terminations allows them to be very hydrophilic, in contradiction of various hydrophobic 2D NMs (e.g., graphene).^[^ ^40^, ^41^ ^]^ Its high conductivity enables their application in current collectors, interconnects, with conductive type ink, etc. MXene NMs can be used in photothermal therapy and electrochromics because of their interband transitions and plasmon resonance peaks that widen an entire UV, visible (vis), as well as NIR bands. These plasmonic properties are chemically and electrochemically tunable. As a consequence of their large interfaces with electromagnetic waves at frequencies range from tera‐to‐giga hertz, they are applicable in electromagnetic interference shielding and communication. The redox activity of TMs atoms on MXene NMs surface allows electrocatalysis; rechargeable batteries as well as supercapacitors (SCs).

The 2D NSs are regulated spaced apart to conduct dialysis, gas separation, and water purification. MXenes' high surface charges enable liquid crystal growth and aqueous molecule hanging out with no requirement of surfactants or binders. For the purpose of synthesizing multilayer (ML) structures and modifying property, 2D MXene NMs’ layer can intercalate organic molecules, polymers, and ions. As the 2D MXene NMs have revealed various chemical, optical, electrical, and mechanical capacities so, the proposal of MXetronics (all‐MXene optoelectronics) was suggested. The 2D Ti‐based MXene NMs, their hybrids and composites with polymers, ceramics, and metals are mostly drawing attentions and since they are readily accessible materials, which are nontoxic and environmental friendly. On the other hand, in this article we will spotlight on current discoveries of non‐Ti based MXene NMs, which use various possible metal atoms in substitution of Ti. The ≈70% above studied MXene NMs are Ti‐based MXenes while the other part is subdivided to other less studied early TM‐based M‐MXenes‐based NM (Figure 2e). On the other hand, there are a small number of articles related to the 2D non‐Ti MXene NMs generally because of their difficult production process based on conventional synthesis techniques. Regardless of their complexity of synthesis, each metal has potential intrinsic properties, with abundance at market price, and once integrated in the M‐based 2D MXene NMs, have extremely fascinating devices uses and even provide amazing properties than the usual Ti‐based MXenes.^[^ ^43^ ^]^ A summary representation in Figure 2f shows the regions covered in this article. The enormous attractiveness of the 2D MXene NMs over past 11 years was outcome in important number of research articles; especially review articles that include their synthesis mechanisms, properties, intercalations, and novel devices applications.^[^ ^5^, ^13^, ^24^, ^44^ ^]^ Yet the majority of these articles are relied in the region of Ti‐based MXene NMs classification that was intensely examined in broad applications, for example, optoelectronics, UV‐type photodetector, biosensor, batteries, electro(photo)catalysts, membranes, and so on.^[^ ^5^, ^13^, ^24^, ^44^ ^]^ At this time, in this article we will give a distinctive evaluation on the challenges related to synthesis, their novel properties and uses of 2D non‐Ti MXene NMs. In other words, we will explain their definite atomic configurations, various properties that occur, and possible uses that these properties allow. Particularly, an evaluation of advantage and disadvantage relative to the 2D non‐Ti MXene NMs family is obtainable to demonstrate their possible impact for increasing this novel materials research field of NMs. At the end we will explain in detail about the future views as well as suggestions to the direction of non‐Ti MXene NMs growth, properties, and novel applications.

## 2 Important Factor to Know before MXenes Synthesis

### 2.1 Why Non‐Ti MXenes Are Important to Study

Here are a few reasons why 2D non‐Ti MXene NMs based research should be taken seriously.

1. Unique properties: Non‐Ti MXenes exhibit unique properties, such as high electrical conductivity, mechanical strength, thermal stability, and good electrochemical and thermal properties, which make them attractive for a variety of applications. Exploring the potential of these materials could lead to the discovery of new materials with novel properties and applications.
2. Broad range of applications: Non‐Ti MXenes have been shown to have potential applications in areas such as energy storage, catalysis, sensors, electromagnetic interference (EMI) shielding, structural materials, coatings, and more. As such, exploring non‐Ti MXenes could lead to the development of new technologies and materials that could have a significant impact on various industries.
3. Sustainability: As previously mentioned, non‐Ti MXenes may be synthesized from more abundant and environmentally friendly sources, making them a more sustainable alternative to Ti‐based MXenes. With growing concerns about sustainability and environmental impact, the development of sustainable materials is becoming increasingly important.
4. Potential economic benefits: The development of new materials and technologies based on non‐Ti MXenes could lead to economic benefits for countries and companies investing in research and development. For example, the development of new energy storage materials could have significant economic benefits in the renewable energy industry.

In summary, non‐Ti MXenes research should be taken seriously due to their unique properties, broad range of potential applications, potential sustainability benefits, and potential economic benefits. Now we will explain various factors those strongly affect on the properties as well as applications of non‐Ti MXene NMs.

### 2.2 Chemistry of MXene Precursors

**Figure 3:**
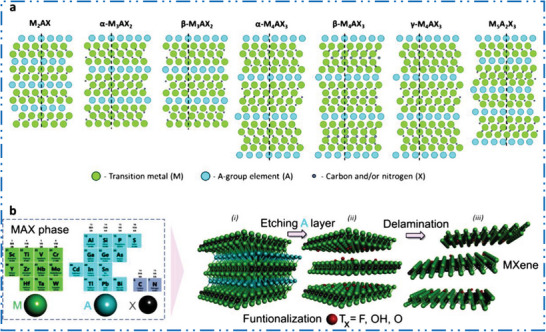
a) Various MAX crystal structures, schematics of the (1120) planes in various MAX phases. Reproduced with permission.^[^ ^45^, ^50^ ^]^ Copyright 2013, John Wiley & Sons. b) schematic illustration of MAX to MXene transformation. Reproduced with permission.^[^ ^37^ ^]^ Copyright 2023, Elsevier.

The beginning of an accomplishment and future of the 2D MXene NMs as NMs was made possible by MAX predecessors. Using the general formula M*_n_*_+1_AX*_n_*, Barsoum^[^ ^45^ ^]^ studied a new group of solids, where M is early TMs, A is an element from group‐(12–16), and X is carbon or nitrogen (**Figure** **3**a). Value of *n* signifies the configuration of M*_n_*_+1_AX*_n_* layers as 211, 312, and 413, for M_2_AX, M_3_AX_2_, and M_4_AX_3_, respectively. The majority of MAX phases are reported having *P63/mmc* space group hexagonal crystal structures.^[^ ^46^ ^]^ Layers of “A” elements positioned in center of trigonal prisms are placed between layers of edge‐shared M6X‐octahedra in their crystal structure.^[^ ^47^ ^]^ The non‐MAX phases of Sc, Zr, and Hf, by contrast, are predicted to form layered compounds with alternate NaCl‐type M‐C and Al_4_C_3_‐like Al(A)‐C sublayers. The typical formula for the non‐MAX precursors is (MC)*_n_* [Al(A)]*_m_* C*_m_*_‐1_, where *n* is 2–4, *m* is 3–4, and A is Si and/or Ge.^[^ ^48^ ^]^ The formulas M_5_A_2_X_3_ and M_7_A_2_X_5_ have also been used to find hybrid MAX precursors with metastable phases (Figure 3a).^[^ ^49^ ^]^ In Ti–Si–C arrangements of Ti_5_Si_2_C_3_ with combinations of 312 and 211 MAX precursors and Ti_7_Si_2_C_5_ with combinations of 312 and 413 MAX phases, the hybrid MAX phases were first evaluated.^[^ ^49^ ^]^ Recent studies have shown that alloying a new novel MAX precursors can produce quaternary MAX precursors materials that exhibit out‐of‐plane MAX (o‐MAX) or in‐plane MAX (i‐MAX) type arrangements,^[^ ^48^ ^]^ which then permitted the creation of novel MXene‐based NMs.^[^ ^33^ ^]^ More than 155 MAX formulations have been tested experimentally and conceptually as of this writing.^[^ ^50^ ^]^ As their mixed metallic‐covalent character, MAX precursors feature laminated structures with comparatively strong M–X along with a weak M–A bonds (Figure 3b(i)). As a result, the solid MAX phase can be broken into MX layers with accordion type structure,^[^ ^51^ ^]^ which is seen in Figure 3b(ii). This renders M–A bond sensitive to careful acid‐based etching. Delamination processes, such as ultrasonication, are subsequently utilized to relax the accordion‐like configuration in order to create a 2D few layer (FL) MXene NMs, as shown in Figure 3b(iii). Even though bottom‐up techniques were employed to synthesize some MXene NMs, but majority of 2D MXene NMs are synthesized by means of top‐down technique and so derives their formation and nanocomposition from their particular bulk layered MAX carbide/nitride precursor‐phases.^[^ ^34^ ^]^ Because of their stability with M as well as X vacancies or mixed occupancies, bulk TMCs and TMNs have a wide range of chemical compositions and morphologies.^[^ ^52^, ^53^ ^]^ First effective exfoliation of Ti_3_AlC_2_ MAX precursor in 2D Ti_3_C_2_ form with ML configurations was reported by Gogotsi and co‐workers.^[^ ^32^ ^]^ Since then, as a minimum 100 stoichiometric MXene based compositions were excrementally as well theoretically studied, with Ti_3_C_2_T*_x_* receiving more than 70% of attention in MXene NMs novel research.^[^ ^54^ ^]^ In addition, properly designed synthesis of the o‐MAX, i‐MAX, non‐MAX, and hybrid MAX has raised a prospect of the emergence of additional types of MXene NMs, thereby extending the list of MXene NMs. MXene family has recently referred to this historic richness of research on bulk TMCs and TMNs to regulate a chemistry of precursors as well as produce 2D MXene NMs with adjustable and/or great properties. Thus far as MXene precursors, the compositions produced have taken advantage of the chemical variety of layered TMCs/TMNs, e.g., both MAX and non‐MAX phases.^[^ ^55^ ^]^ General chemistry of these precursors is discussed in this section, and how their configuration and stoichiometry impact the fabrication and characteristics of resultant MXenes.

### 2.3 Precursor Chemistry to Predict Exfoliation Energy

**Figure 4:**
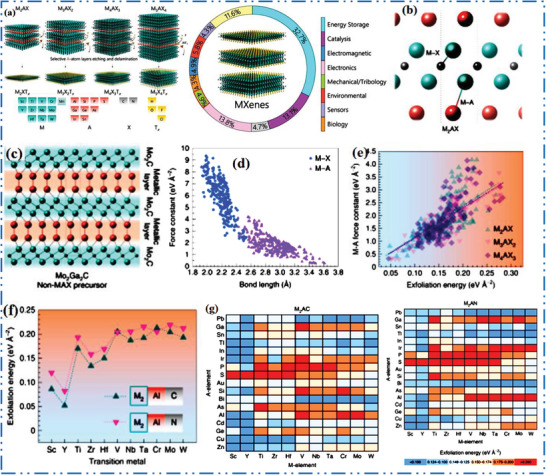
a) MAX with probable M–A–X compositions as well as capability to make various *n*‐based configurations (*n*  =  1, 2, 3, 4, M_5_AX_4_ not shown), b) scheme shows M–X besides M–A bonds in MAX precursors, d) M–A as well as M–X bond lengths, with bond force constants, e) exfoliation energy of precursor raise with higher M–A bond force constants, g) heat map of M_2_AX precursor exfoliation energy with different M (horizontal), A (vertical), and X (top, bottom) compositions. Reproduced with permission.^[^ ^63^ ^]^ Copyright 2022, Springer Nature.

Generally, majority of experimentally produced novel 2D MXene NMs are synthesized from their bulk MAX precursors, which are physically characterized via M*_n_*_+1_X*_n_* layers interleaved by single layer (SL) of group A‐atoms in MAX phases (**Figure** **4**a).^[^ ^35^ ^]^ The M*_n_*_+1_X*_n_* layers are kept jointly in these precursors via strong ionic/covalent M–X bonds inside a layers and weak metallic bonds (M–A) in layers (Figure 4b). A few number of non‐MAX precursors were exfoliated into 2D MXene NMs’ feature comparable to M–X and M–A bonds, for example, Mo_2_CT*_x_* from Mo_2_Ga_2_C (Figure 4c). As these disparities in binding strength so, exfoliation is possible by a cleave M–A bonds while retaining M*_n_*_+1_X*_n_* 2D layers.^[^ ^56^ ^]^ Precursors are frequently put in liquid acidic etchants^[^ ^57^ ^]^ or molten salts^[^ ^58^ ^]^ to exfoliate the M*_n_*_+1_X*_n_* layer of MXene NMs. Because early TMCs/TMNs are highly stable in such applied atmosphere.^[^ ^59^ ^]^ The M*_n_*_+1_X*_n_* layers remain intact while M–A bonds are targeted by particular etchant, resulting in selective etching and loss of A layers. Relative potency of the present chemical bonds, especially a strength of M–X contrast to M–A bonds,^[^ ^60^ ^]^ is an important element in the effective separation of 2D M*_n_*_+1_X*_n_* MXene NMs layers from their bulk‐predecessor phases. As SL crystals of MAX as well as non‐MAX precursors were segregated through shearing at M–A interfaces,^[^ ^61^ ^]^ the influence of these differing bond strengths in MAX and non‐MAX predecessor phases may be detected in the 2D MXene NMs synthesis using mechanical exfoliation. Differences in composition (M, A, and X) and bond chemistry also have an impact on the configuration, for instance, the ordering of M layers (such as o‐MAX compared with i‐MAX predecessor phases^[^ ^62^ ^]^). From a practical standpoint, additional aspects, for instance, etchant solution and synthetic techniques influence the effectiveness of the MAX to 2D MXene family members. These synthesis aspects govern a thermodynamic possibility and kinetics of etching response.

Usually, MAX and non‐MAX precursors exfoliation energy to the 2D MXene NMs are determined by comparative M–X (vs M–A) bond strength, which is estimated by bond force constants (Figure 4d).^[^ ^60^ ^]^ When both links are strong then poor exfoliation into 2D MXene NMs take place, and when both bonds are weak, MAX precursor phases dissolve at some point in etching.^[^ ^63^ ^]^ Precursor compositions with 1) very weak M–A bonds and 2) extraordinarily strong M–X bonds, for instance, have reduced exfoliation energy and are therefore simply exfoliated to a various MXene NMs. Trend (1) in computed exfoliation energy and M–A bond force constants is shown in Figure 4e.^[^ ^64^ ^]^

Regardless of their bulk MAX phase precursors’ configuration (M*_n_*_+1_AX*_n_*, *n* = 1–3), the examined MAX predecessor phases were anticipated to have the similar growing exfoliation energy development with greater M–A force constants.^[^ ^60^ ^]^ This finding suggests that the energy of MAX‐to‐MXene involved is determined less by MAX configuration (*n*‐value) and further by surface TMs M occupied in both M–X and M–A bonding. The *n*‐value's significance is addressed in etching with exfoliation system part. Chemical identity of X (X = C or N)‐trend (2) in Figure 4f also substantially influences M–X bonds’ strength and thus exfoliation energy.

Nitrides are projected to have greater exfoliation energy than carbides with a similar configuration and M atoms,^[^ ^60^ ^]^ for example, using M_2_AlX MAX predecessor structure (Figure 4f).^[^ ^57^, ^65^ ^]^ This discrepancy was ascribed to poorer M–N (vs M–C) bond strength caused by an extra valence electron in N (vs C)^[^ ^66^ ^]^ and other electron positions other nitride (vs carbide) MAX candidates over stability barrier of 3.6 to 4.5 electrons per atom. Therefore, nitride MAX phases are more unstable than carbide MAX phases, which may explain why there are less nitride MXene NMs than carbide MXenes family members.^[^ ^67^ ^]^ The M–A pairings with fewer M and/or A atoms and/or a greater number of valence electrons often make a strong M–A bonds, resulting in a slightly variation in M–X and M–A bond energies.^[^ ^68^ ^]^ MAX exfoliation varies considerably in M–A compositions for both TMCs as well as TMNs are shown in Figure 4g. Consequence of high M‐atoms, with high valency expects a lower exfoliation energy,^[^ ^68^ ^]^ and is revealed in comparatively simple exfoliation.^[^ ^69^ ^]^ When contrast the exfoliation energies, the outcome of high valency and small atomic radii in A elements is also obvious.^[^ ^57^, ^70^ ^]^ In addition to MAX precursors phase, which have proven experimentally challenging to exfoliate into various 2D MXene‐based NMs, compositional manipulation of chemical bonding was also used in non‐MAX precursors phase. For instance, it has not been able to synthesize 2D Mo_2_CT*_x_* MXene by exfoliating a single Ga‐layer from the MAX Mo_2_GaC precursor phase. Instead, Mo_2_CT*_x_* was created by synthesizing the non‐MAX Mo_2_Ga_2_C layered phase (Figure 4c), which relies by etched a weak Mo–Ga along with Ga–Ga metallic connections. This phase has two atomic layers of Ga in layers of 2D Mo_2_C NM. Another illustration is a challenging production of pure Hf_3_C_2_T*_x_* because Hf_2_AlC is frequently found in Hf_3_AlC_2_ MAX phases,^[^ ^71^ ^]^ resulting in low phase purity of produced Hf_3_AlC_2_ MAX phases.

Non‐MAX Hf_3_Al_4_C_6_ precursor, which consists of covalently bound Al_4_C_4_ layers sandwiched by Hf_3_C_2_ layers,^[^ ^55^ ^]^ is a novel approach. The interfacial M‐(Al, Si) bonding in the Al_4_C_4_ layers diminishes when Si is added, resulting in a (Al, Si)_4_C_4_ solid solution, which lowers the exfoliation energy of the layers toward 2D Hf_3_C_2_T*_x_* type MXene based NMs.^[^ ^55^ ^]^ Therefore, 2D MXene NMs that would normally be challenging to exfoliate using MAX precursor phase can be produced by intentional compositional study of non‐MAX phases. In summary, both MAX and non‐MAX precursors may be designed to a great extent depending on composition because of the distinction between the metallic M–A bonds and the ionic/covalent M–X interactions. Range of 2D MXene precursors and subsequently the 2D MXene NMs will increase with further experimental investigation across various M–A–X chemical compositions, directed by their chemical bonding behavior.

### 2.4 Precursor Synthesis Effect on MXene Structure

**Figure 5:**
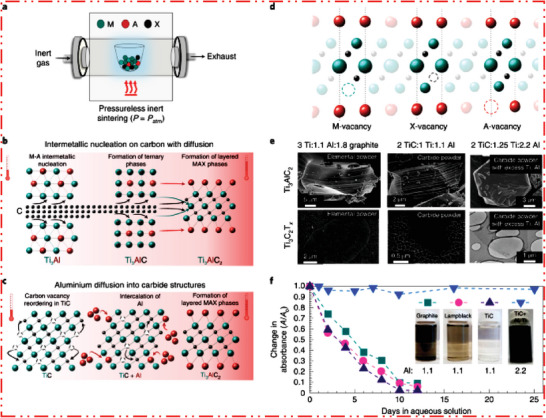
a) Production of MAX, b) employing elemental powder type precursors, production of Ti_3_AlC_2_ precursor starts by Ti–Al intermetallic creation, afterward carbon diffusion in intermetallics to synthesize Ti_3_AlC_2_. c) Production of Ti_3_AlC_2_ precursor phase via carbide powder precursors, d) production of MAX precursors forms M, A, and/or X vacancies, and e) selection of precursors have a diverse impact on MAX grain size/shape and properties of resulting MXene nanoflakes. f) Defects in MAX converted to synthesize expected MXene NMs. Reproduced with permission.^[^ ^63^ ^]^ Copyright 2022, Springer Nature.

Next step is to further evaluate the research study synthesis of the MAX precursor for the 2D MXene NMs after taking into account how chemical bonding in MAX as well as non‐MAX phases affects their exfoliation energy. According to a top‐down strategy from precursor to 2D MXene NMs, future MXene characteristics will be significantly influenced by handling actions to synthesize MAX and non‐MAX precursor phases. Here, we talk about how characteristics and purity of MAX phases are affected by elemental phase precursors (M, A, and X) and heat treatment. Precursor phases are frequently created at atmospheric pressure, direct sintering in applied inert gas based atmosphere (**Figure** **5**a). This procedure produces porous sintered billets^[^ ^72^, ^73^ ^]^ and makes it easier to convert them into powders for etching. Dense MAX precursors bodies created by hot pressing and hot isostatic pressing, although they are more challenging to crush and grind into powders. A pristine elemental powder combination of M, A, and X or carbide/nitride powder mixes of MX and/or AX, with further elemental powder of M, A, and X were employed to create the majority of MAX precursors for synthesis of various members of 2D MXene NMs family.^[^ ^74^ ^]^ Also metal hydrides are used.^[^ ^75^ ^]^ Additionally, impetus is growing for MAX phase production in molten salts, which can be expanded to include the 2D MXene production.^[^ ^76^ ^]^ These powder mixture combinations lead to highly unique production routes for MAX‐phase (Figure 5b,c).^[^ ^72^ ^]^ Therefore, intermetallic M–A compounds are initially formed during the reactive heat treatment of precursor powders, and that these compounds then nucleate binary carbide layers on a carbon interface to produce nonlayered ternary M–A–X precursors.^[^ ^77^, ^78^ ^]^ According to other studies, Ti_2_AlC is transformed into Ti_3_AlC_2_ in the presence of TiC*_y_*. According to this research, the formation route for the MAX phase employing powdered carbide or nitride mixes depends on the diffusion of M and/or A atoms into a configuration with assistance of carbon or nitrogen vacancies (Figure 5c). The MAX precursor's structure is significantly influenced by initial precursor powders used and various formation methods employed to synthesize MAX phases. Examples include variances in MAX grain size/shape were on micro/macroscopic scale (Figure 5e, top row) and defects, for example, vacancies (M, A, and/or X; Figure 5d), etc. The resultant 2D MXene NMs form and behavior are directly related to these differences in MAX structure and stoichiometry.^[^ ^79^ ^]^

MAX‐precursor phase sintering temperature has an impact on electrical conductivity. The formation of defects (vacancies) or absences of stoichiometric ratio in elements during higher temperature synthesis are the two main causes of low MXene electrical conductivity. Synthetic MXene NMs with a modest usual nanoflakes’ size with noticeably increased conductivity after removal of impurity and HF/HCl etching are observed. This progress was credited to a rise in carbon substance,^[^ ^74^ ^]^ which raises the possibility that abundance of metals may remove oxygen from heat treatment atmosphere as well as avoid carbon removal, which causes reduction in vacancies (or substitutional oxygen). Moreover, because defects in the 2D MXene NMs’ structure significantly increase MXene oxidation.^[^ ^79^ ^]^ The rate at which delaminated MXene NMs degrade in aqueous colloidal solutions might qualitatively reflect the defect focus in the bulk MAX precursors employed.^[^ ^74^ ^]^ In conclusion, it is possible to anticipate the exfoliation energy of precursors used for 2D MXene NMs synthesis by using the chemical composition of M–A–X. Formation process, elemental precursors selection, their ratios, and applied heat treating situations all need to be taken into account while manufacturing these MAX as well as non‐MAX materials since they have an impact on the characteristics of the precursor that is synthesized and, ultimately, the 2D MXene NMs. Even though these factors can significantly influence the characteristics of 2D MXene NMs, they are still under examined in larger MXene NMs literature, mainly for MXene NMs other than Ti‐based carbide MXene NMs. Future research should examine the impacts of M, A, and X composition as well as the processes by which MAX as well as non‐MAX precursors are formed.

### 2.5 Surface Terminations, Intercalation, and Delamination Strategies of MXenes

**Figure 6:**
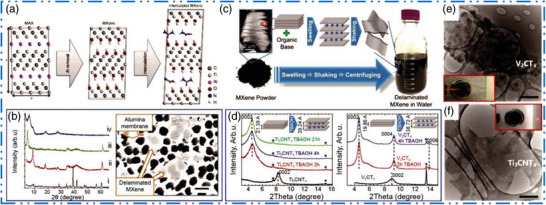
Intercalation–delamination mechanism. (a) Intercalation of Ti_3_C_2_T*_x_*. b) XRD of (i) Ti_3_AlC_2_, (ii) exfoliated, (iii) DMSO intercalated, and (iv) delaminated Ti_3_C_2_T*_x_* (left). SEM of delaminated Ti_3_C_2_T*_x_* on alumina membrane (right). c) Scheme of MXene delamination route via organic base, d) XRD of Ti_3_CNT*_x_* before and after mixing with TBAOH, e) TEM of a delaminated V_2_CT*_x_* NS and f) a delaminated Ti_3_CNT*_x_* NS. Insets show the Tyndall effect for V_2_CT*_x_* and Ti_3_CNT*_x_* aqueous solution, respectively. Reproduced with permission.^[^ ^42^ ^]^ Copyright 2022, Wiley.

Surface termination of as‐synthesized 2D MXene NMs is primarily dependent on the synthesis method we applied. While OH, F, and O are most often mentioned surface terminations in 2D MXene NMs, theoretical calculations that have also demonstrated the potential electrical, magnetic, chemical, as well as optical features of additional nonmetal surface T*_x_* including S, Se, Te, and Cl.^[^ ^80^ ^]^ Therefore, analysis of alternative functional group T*_x_* could offer certain surface functions that are useful in some applications. Depending on 2D MXene and etching conditions, mixed O, F, OH, and T*_x_* are also typically seen when aqueous HF is utilized.^[^ ^81^ ^]^ By means of liquid chloride salts, Cl‐based termination prevailed^[^ ^58^ ^]^ and when NH_4_HF_2_ employed, then F‐rich surface termination achieved.^[^ ^82^ ^]^ Termination can also be changed by postsynthesis processes.^[^ ^83^ ^]^ In their as‐synthesized 2D MXene NMs, which were produced by etching MAX precursors in molten CdBr_2_ and CdCl_2_. In the same way, Kamysbayev et al.^[^ ^39^ ^]^ studied Br and Cl based termination with S, Se, Te, and NH via postsynthesis effects with Li and Na salts in molten bromides at 300 °C, afterward heat treatment and anhydrous washing. Their ability to change and modify terminations expands the already vast compositional space available to them. Through intercalation and delamination, ML MXene NMs can further be converted in an SL or FL 2D NSs. The attributes of their resultant 2D MXene nanoflakes are similar to those of accordion like MXene NMs, including rich surface chemistry, strong hydrophilicity, high conductivity, and a number of standout qualities including exceptional flexibility and wide interlayer spacing. To successfully delaminate an ML of 2D MXene NMs, it is essential to dissolve the dominant bonds between them. The use of ultrasonication allowed the delamination of 2D MXene nanoflakes that were only FLs thick. Although the yield is far from acceptable, this is because of strong contacts between 2D MXenes layers that are only sporadically broken by shearing force produced by ultrasonication. It has been demonstrated that introducing intercalators, both organic and inorganic, is a successful tactic for weakening the interlayer connections and promoting delamination (**Figure** **6**a). Since the discovery of 2D MXene NSs, several intercalation and delamination combinations have been produced by intercalator dimethyl sulfoxide (DMSO).^[^ ^84^ ^]^

Other organic solvents, including as urea and *N*,*N*‐dimethylformamide (DMF) were also utilized as intercalates for delaminating ML 2D MXene NMs in addition to DMSO. However, even with the intercalation of the DMSO and its family, the yield of delaminated NSs is insufficient. Additionally, intercalations using DMSO have failed to prevent the delamination of various other 2D MXene NMs.^[^ ^35^ ^]^ It has been demonstrated that a group of organic alkali with comparatively great molecular configurations, for instance, *n*‐butylamine and tetrabutylammonium hydroxide (TABOH), intercalate different 2D MXene NMs more effectively (Figure 6c).^[^ ^85^ ^]^ Alkali cations would intercalate in MXene NMs layers and greatly enlarge their interlayer space following the dissociation of organic alkali in aqueous solution of ML 2D MXene NMs (Figure 6d). Therefore, hand shaking or moderate ultrasonication may cause ML MXene NMs to delaminate. Using aforementioned intercalation technique, SL V_2_CT*_x_* NSs were also produced, in addition to other MXene NMs (Figure 6e,f). In MXene NSs, employing organic alkali can lower F‐group concentrations and raise O_2_ value, both of which are advantageous for some applications, like in energy storage devices, e.g., batteries. In addition to intercalator screening, intercalation technique optimization also merits special attention since it can result in optimal ML MXene delamination.^[^ ^86^ ^]^ Even vigorous ultrasonication, delamination yield of MXene NSs in classical production is low.

TMAOH and ascorbic‐acid (AA) used as intercalator and reductant, respectively, in a hydrothermal‐assisted intercalation technique to enlarge intercalator diffusion.^[^ ^86^ ^]^ Intercalation of TMAOH and subsequent delamination of ML MXene NMs were both aided by a microwave treatment, with low yield.^[^ ^87^ ^]^ According to earlier studies, 2D MXene NMs would become unstable when oxygen and water mix, especially at high temperatures.^[^ ^88^ ^]^ As a result, during the intercalation, MXene NSs would produce a large number of defects, which would cause them to be unstable and have a low conductivity.^[^ ^89^ ^]^ Additionally, extended hydrothermal and ultrasonic treatments would result in a reduction in the lateral diameters of MXene NSs. Freeze‐and‐thaw (FAT)‐assisted technique has been developed to cleave MXene from ML MXene NMs based on expansion force of water‐freezing to address the aforementioned issues. The FAT development increased the yield of 2D MXene NMs with further ultrasonication, but the lateral size naturally shrank. The MXenes NSs produced by FAT approach demonstrated good conductivity and high mechanical strength as a result of the moderate circumstances of their synthesis, making them well suited for bendable renewable energy storage systems. Etching continues to pose more of a difficulty for the MXene NMs synthesis than intercalation and delamination. These adaptable and electrically conductive MXene NSs may diffuse uniformly in a range of solutions^[^ ^90^ ^]^ and aggregate into freestanding films, flexible fibers, compressible sponges,^[^ ^91^ ^]^ and vertical arrays, among other structures. Through creation of an excellent utilization of their surface chemistry, MXene NMs have extra functions, such as superconductivity.^[^ ^92^ ^]^

### 2.6 Diversity of Structures and Compositions

MXenes family benefits directly from any development for production of MAX phases with different composition and structure variety; in fact, the synthesis of 2D MXene NMs has been a major driving force behind many recent investigations on MAX phases. To illustrate this connection, we will provide a few examples of these technological developments. Figure 1 depicts the known structures of 2D MXene NMs schematically. In 2D MXene NMs feature which have hexagonal close‐packed crystal configuration with *P63/mmc* space group symmetry, similar to the basal planes in MAX precursors (where TMs in M sites are close‐packed and X atoms occupy the octahedral sites in M atomic planes).^[^ ^38^ ^]^ There have been reports of 2D MXene NMs containing Mo, Cr, Ti, Nb, V, ZrHf, Sc, W, Ta, and Y at M position. It should be noted that Cr, Sc, W, and Y were only recorded as components of o‐(i)‐MXene NMs, together with the other metals indicated above. At least 26 distinct o‐MXene NMs were calculated in theory, including Mo_2_ScC_2_T*_x_*, Cr_2_TiC_2_T*_x_*, Mo_2_TiC_2_T*_x_*, and Mo_2_Ti_2_C_3_T*_x_* having previously been experimentally observed.^[^ ^93^ ^]^ Ideal o‐MXene composition has an M′ to M″ ratio of 2:1 or 2:2, derived from an appropriate ratio of various metal lattice locations in 2D MXene configuration. By contrast, 2D i‐MXene NMs are preferred only when the ratio of M′ to M″ is 2:1 and the size difference in M″ and M′ is at least 0.2.^[^ ^94^ ^]^ In MXenes M′ atoms form a honeycomb lattice and M″ atom positioned on the hexagon centers and extend out from the M layers. A monoclinic (*C_2_*/*c*) or orthorhombic (*C_2_*/*mor Cmcm*) configuration of i‐MAX phases is inherited by 2D i‐MXene NMs.^[^ ^95^ ^]^ Up to now, 32 distinct i‐MAX phases were synthesized.^[^ ^96^ ^]^ However, most i‐MAX phases have both A and M″ components scratched away at the same time. Using HF, for example, Al and Sc or Y were selectively eliminated from (W_2/3_Sc_1/3_)_2_AlC and (W_2/3_Y_1/3_)_2_AlC, yielding W_1.33_CT*_x_* i‐MXene with ordered divacancies.^[^ ^97^ ^]^ This phenomenon results from M″ elements' poorer connection to carbon sites, as shown by their outward displacement from M plane. These structural properties also enable a notion of targeted etching, in which adjusted production circumstances can assist removal of either A alone or both A and M″ components from the i‐MAX, as demonstrated for Mo_4/3_Y_2/3_AlC.^[^ ^98^ ^]^

For many years, solid solutions of MAX phases containing several TMs have been discovered.^[^ ^99^ ^]^ They just lately become aware of their 2D MXene NMs, per force; here, we provided few examples.^[^ ^100^ ^]^ For example, double TMs based MAX phase with ordered structure Cr_2_TiAlC_2_ was discovered in 2014. Ti‐atoms dominate the core layer of M in this o‐MAX (*P63/mmc* space group), while Cr atom nearly entirely resides in the outside layers of M (4f Wyckoff sites) in the M_3_C_2_ blocks, while Ti‐atom occupies a central layer (2a Wyckoff sites).^[^ ^101^ ^]^ Anasori et al.^[^ ^33^ ^]^ also studied on three novel o‐MXene NMs and identified Mo_2_TiAlC_2_ and Mo_2_Ti_2_AlC_3_ (Mo_2_TiC_2_T*_x_*, Mo_2_Ti_2_C_3_T*_x_*, and Cr_2_TiC_2_T*_x_*). The o‐Mo_2_ScAlC_2_ and its equivalent MXene Mo_2_ScC_2_T*_x_* were reported on by Meshkian et al. in 2017.^[^ ^102^ ^]^ Tao et al. revealed the existence of a different sort of layered ordered double TMC, namely, (Mo_2/3_Sc_1/3_)_2_AlC, in which two TMs (Mo and Sc) are carbides based i‐MXenes NSs. Sc‐atoms are also etched in etching method. As a result, Mo_4/3_CT*_x_* MXene acquires ordered vacancies at the Sc atoms’ original M locations.^[^ ^103^ ^]^ New TMs for the MAX precursors, for example, (W_2/3_Y_1/3_), are present in additional in‐plane ordered MAX. There have been reports of 2AlC (W or Y alone does not form a MAX precursor),^[^ ^104^ ^]^ as well as their analogous MXene NMs with ordered vacancies (e.g., W_4/3_CT*_x_*). By etching Sc and Al from (Nb_2/3_Sc_1/3_)_2_AlC, random vacancies at M sites for Nb_1.33_CT*_x_* were discovered.^[^ ^105^ ^]^ According to Persson et al.,^[^ ^106^ ^]^ etching (Mo_2/3_Y_1/3_)_2_AlC, which displayed in‐plane ordering, under the right circumstances can leave behind an in‐plane ordered double M compound called (Mo_2/3_Y_1/3_)_2_CT*_x_*) MXene while just removing the Al layers. Only small impurity phases of MAX precursors with *n* > 3 have been found in bulk production or sputtered thin films.^[^ ^107^ ^]^ By contrast, Deysher et al.^[^ ^108^ ^]^ recently studied on the bulk powder metallurgy synthesis at 1650 °C of a *n* = 4 phase with a composition of (Mo_0.8_V_0.2_)_5_AlC_4_. The formation of (Mo_0.8_V_0.2_)_5_C_4_T*_x_* by successfully etching Al makes way for the synthesis of other MAX and MXene compounds. There are boundless possibilities for novel MXene compositions thanks to the high‐entropy 2D MXene NMs with numerous M elements^[^ ^109^ ^]^ and random binary solid solution on M sites. As this is a potent method for modifying the properties of MXene NMs, theoretically and structure space of MAX precursors and their equivalent MXene NMs will continue to grow in the upcoming years, particularly in direction of more than one M per MXene NMs (solid solution, o‐MXenes, and/or i‐MXenes type structures).^[^ ^110^ ^]^

Carbon, nitrogen, or both can occupy the X sites with exemption of ml‐Ti_2_NT*_x_* and ml‐Ti_4_N_3_T*_x_*,^[^ ^65^, ^111^, ^112^ ^]^ research on nitride 2D MXene NMs was restricted since production of nitride MXenes is somehow difficult. There are far fewer studies on nitride as well as carbonitride type MXenes than there are on C‐based MXenes.^[^ ^111^ ^]^ This is partially due to the insufficiency of MAX phases that contain nitrogen and challenges associated with synthesizing nitride MXene NMs because a nitride layers tend to dissolve in acids. It is thought that in carbonitrides MXene NMs, the carbon and nitrogen atoms reside in the octahedral positions at random, regardless of carbonitride stoichiometry.^[^ ^113^ ^]^ On the other hand, further research is needed to have a better knowing organization of X site atoms in these MXene NMs. In bulk TMCs as well as TMNs, oxygen may substitute for both carbon and nitrogen in the lattice, generating oxycarbides or oxynitrides, correspondingly. It is essential to investigate the possibilities of such replacement in MXenes. It is still anticipated that with continued dedication, other nitride MAX precursors will eventually be created, and their etching will be exploited to create 2D nitrides. The Mo_2_NT*_x_*, V_2_NT*_x_*, and nitrogen‐doped Ti_2_CT*_x_* have all been successfully produced using other cutting‐edge methods, for example, nitriding 2D MXene NM to substitute some or all of carbon with nitrogen, which significantly improved their performance as electrocatalysts.^[^ ^114^ ^]^ Other layered carbides, such as Zr_3_Al_3_C_5_, Hf_3_(AlSi)_4_C_5_,^[^ ^55^ ^]^ and Mo_2_Ga_2_C, were effectively employed to manufacture Zr_3_C_2_T*_x_*, Hf_3_C_2_T*_x_*, and Mo_2_CT*_x_*, respectively. This proves that production of MXene NMs by selective etching is not confined to MAX precursor materials. Other bottom‐up methods, for instance, CVD technique, were employed to produce large‐area ultrathin‐Mo_2_C, WC, and TaC films additionally to selective etching and top‐down synthesis. By reducing hexagonal oxides in ammonia, Xiao et al.^[^ ^115^ ^]^ studied on salt‐templated formation of 2D MoN, V_2_N, as well as W_2_N.

Reliant on production technique and 2D MXene composition, MXene NMs surface has single or mixed T*_x_* (T*_x_* = NH, OH, Br, S, Se, O, F, Te, Cl, etc.). Postprocessing can change or delete these terminations altogether,^[^ ^39^, ^116^ ^]^ which has a significant impact on materials characteristics. MXenes with mixed terminations are produced via synthesis in F and Cl containing acidic solutions, with a composition of T*_x_* (surface groups) in 2D MXene NMs’ formula being (OH)*_m_* O*_x_* F*_y_* Cl*_z_*.^[^ ^117^ ^]^ Depending on the synthesis technique, density functional theory (DFT) predictions, and the use of NMR, elastic neutron scattering, XPS, and STEM techniques have recommended a random allocation for OH, O, in addition to F with a changeable ratio. Typically F and O T*_x_* predominate over OH groups in the dry state.^[^ ^118^, ^119^ ^]^ In relation to M and X atoms, T*_x_* atoms can be located at the surface in a variety of locations. The face‐centered cubic (FCC) site, which is centered over TM atoms of atomic plane under an external layer, is the most actively advantageous and thermodynamically stable location for these moieties on a surface of M*_n_* C (*n* = 2, 3, 4) layers.^[^ ^120^ ^]^ Functional groups may also organize themselves on a surface, so that they are positioned above the X atoms (hcp sites). The STEM investigations have demonstrated that F atoms only inhabit FCC sites, although O atoms can dwell at both sites, despite competition at ambient temperature in various groups to exist in favored thermodynamically stable locations. Thermal processing and vacuum annealing allow for the modification of the composition and coordination of MXene NMs' T*_x_*.^[^ ^121^, ^122^ ^]^ There are two forms of intercalated (structural) water found in the configuration of MXene NMs made via aqueous etchants: physisorbed and chemisorbed. The SL of chemisorbed water with H_2_ bonding to a surface may still intercalated in the 2D MXene NMs, necessitating vacuum heat treatment at above 500 °C for taking away. Physisorbed H_2_O may be detached at lower 200 °C heat treatment.^[^ ^123^ ^]^ The CuCl_2_ or CdCl_2_ salt melts can be generated by etching in Lewis acids to yield Cl‐terminated ML MXene NMs (M*_n_*_+1_X*_n_* Cl_2_).^[^ ^58^ ^]^ To create Br terminations, bromides such as CdBr_2_ can be employed instead of CdCl_2_.^[^ ^39^ ^]^ In order to create a collapsed 3D structure with significant interlayer contact between the 2D MXene NSs, these homogeneous halogens based terminations (particularly Br) at the surface of MXenes must be deleted. This structure is comparable to electrides.^[^ ^124^ ^]^ Through subsequent surface reactions, these terminations can also be altered for different functional groups, extending a variety of 2D MXene NMs’ compositions. The production of MXene NMs with Te, Se, NH, O, and S based T*_x_* is comparable. According to assessments using Rietveld analysis with EXAFS, and pair distribution function, the type of uniform T*_x_* that causes in‐plane compressive or tensile strain causes a minor alteration in the structure of MXene NMs.^[^ ^39^ ^]^

## 3 General Synthesis Methods and Processing of Non‐Ti MXenes

Due to clumsy size, the ML 2D MXene applications in several fields, including as nanoelectronics and medical, may be severely constrained.^[^ ^125^ ^]^ MAX (and non‐MAX) phases are designed as well as synthesized by etched, and exfoliated method to get MXene NMs. Since innovative of first MXenes, then, several other methods have been introduced, ranging from electrochemical, alkaline, molten salt, and halogen etching^[^ ^35^, ^126^ ^]^ to direct and indirect HF‐based synthesis. It is possible to further delaminate weakly bound ML 2D MXenes into few layers (FLs) to SL‐type NSs. The initial steps toward understanding why particular processes simply construct definite MXene NMs and how the characteristics and surface chemistry of 2D MXenes may be regulated through production come from atomicity knowledge of MXene etching and delamination. This section examines the selectivity of various procedures toward various 2D MXenes, highlighting trends and contrasts between the intercalation–delamination and etching–exfoliation techniques currently in use. Therefore To produce SL or FLs 2D NSs, intercalation and subsequent delamination are required. However, the yield of MXene NSs might occasionally be poor because of the robust contact between MXene layers. As a result, several improved techniques developed to produce MXene NSs with a high yield, which might even enable large‐scale manufacturing.^[^ ^127^ ^]^ By splitting the production and processing of MXene NMs into two sections—etching tactics and intercalation and delamination strategies—this subsection reviews the production and characterization of non‐Ti MXene NMs.

**Figure 7:**
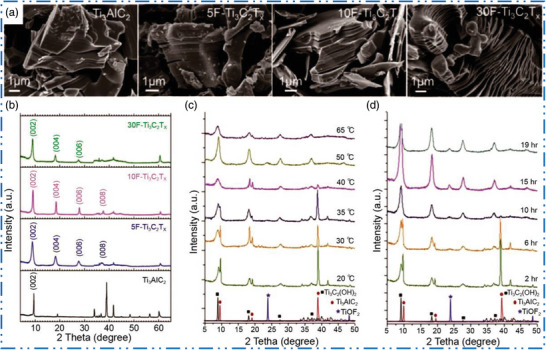
a) SEM of Ti_3_AlC_2_ after etching with 5, 10, and 30 wt% HF. b–d) XRD of MXenes and MAX phases. Reproduced with permission.^[^ ^42^ ^]^ Copyright 2022, Wiley.

a) Etching strategies of MXenes: As the large quantity or bulk of MXenes NM synthesis heavily relied on MAX precursor phase so, MXenes bulk quantity can be synthesized from solid state reaction, whereas large size MXenes from magnetron sputtering (MS), epitaxial production, and CVD technique. High‐quality 2D MXene NMs which are carefully regulated or with no functional groups were given by bottom‐up strategies novel technique therefore; future analysis of different bottom‐up strategies should be looked into using 2D MXene. After using HF MXenes etched at room temperature and variety of MXene NMs was synthesized with various HF concentrations, with different etching durations, at different temperature. According to certain experimental findings, greater HF concentrations might reduce etching time in addition to increase etching effectiveness (**Figure** **7**a,b).^[^ ^57^ ^]^ Figure 7c,d demonstrates that etching temperature and duration may be critical for producing ML 2D MXene NMs.^[^ ^128^ ^]^ As HF is extremely corrosive so, rising etchant concentration, applied etching heat treatment, and time blindly will cause defects to appear quickly and dramatically lower lateral diameters of the 2D MXene NMs.^[^ ^129^ ^]^ Therefore, MXene etching settings should be modified based on the materials' intended characteristics and uses. It is an ideal to introduce a synthesis method by which we can synthesize MXene with large lateral diameters and small defects. This will help out to use MXenes as an electrocatalysts in numerous applications like in fuel cell, batteries, sensors, etc., because these characteristics provide excellent conductivity with strong structural stability that heavily influences rate capability as well as cyclability.^[^ ^130^ ^]^ For that reason, moderate conditions of HF etching procedure, for instance, low HF concentrations, short etching times, and low etching applied heat treatment value, should be adapted to synthesize 2D MXene NMs in order to build high performance devices. Instead of alterations in XRD pattern, the emergence of accordion‐like morphology is sometimes misinterpreted as an indication of effective etching of 2D MXene NMs. In reality, this accordion‐like configuration is primarily due to emission of hydrogen gas in the etching method, which cannot be used to determine how thoroughly the MXene NMs were etched.^[^ ^131^ ^]^ For the production of the 2D MXene NMs with the required characteristics for elevated devices performance, it is advantageous to make effective employing of the XRD investigation, where (002) diffraction peak would expand as well as downshift to a lower angle as a result of enhanced interlayer spacing. Conductivity is affected by the termination group(s) like F groups in HF etching, on outer surface of MXene NMs based on HF etching techniques.^[^ ^132^ ^]^ Thus, it was crucial to introduce alternative methods to replace first introduced HF etching technique.

b) Non‐HF conversion of MAX to MXene: While HF is reputed for being hazardous, making MXene frequently requires the employing such a strong acid. Another efficient etchant for some MAX phases etching to MXenes NMs is a combination of various kind acids (e.g., HCl) and fluoride salts (such as LiF).^[^ ^133^ ^]^ Therefore, non‐HF techniques for making 2D MXene have also been reviewed in this article. One of the potential ways to safely prepare 2D MXene is by the combination of LiF/HCl or FeF_3_/HCl, chemical etching, and other methods. Thus, it is important to carefully assess each of their purity, production yield, surface, as well as physicochemical characteristics as well. The ease of delamination is another benefit of this process over conventional HF etching, additionally to an effective etching with improved safety. After etching, Li‐ions can enter and reside in the space in 2D MXene layers, increasing the interlayer gap. Therefore, MXene NSs may be produced by physical‐shaking or sonication without the need for further intercalation procedures. Theoretically, employing various fluoride salts, an interlayer spacing of MXene NMs may be more restricted to suit needs of various metal‐ion batteries. As LiF/HCl can etch and delaminate MAX precursors in single step, it is still the most used method among the several fluoride salt and acid combinations based techniques.^[^ ^35^ ^]^ Additionally, there are various F‐based etching techniques that can avoid using HF directly. For instance, in water or propylene carbonate, NH_4_HF_2_ may etch A layers.^[^ ^65^, ^82^ ^]^ Currently, the common fluoride etching of MXene NMs unavoidably creates several F‐groups and has a detrimental impact on efficiency of devices, for example, energy storage devices.^[^ ^134^ ^]^ In fact, few F‐free etching techniques were created recently that gave 2D MXene NMs the necessary characteristics.^[^ ^131^ ^]^ A notable example is electrochemical etching, which is F‐free electrolyte eliminates Al atomic layers from MAX precursors. Specifically, 3‐electrode structure with Ag/AgCl, Pt, and HCl as electrolyte, reference, and counter electrodes, correspondingly was used to transform the working electrode of MAX into MXenes and carbide‐derived carbon at 0.6 V.^[^ ^135^, ^136^ ^]^

Alkali etching techniques are yet an additional category of alternatives to prevent fluorine contamination.^[^ ^137^ ^]^ Although the aforementioned methods can get rid of F‐group, but still require corrosive chemicals as necessary elements, posing the same safety issues as conventional HF etching. The majority of the currently used etching techniques are simply efficient for Al‐MAX precursors, which cannot completely use MXene NMs. Therefore, developing a green etching technique for MXene production that is more safe and versatile is difficult. By means of Lewis acidic molten salt etching technique, which has good chemical protection and a broad etching spectrum, a substantial breakthrough was recently made.^[^ ^58^ ^]^ Using ZnCl_2_ or CuCl_2_ as Lewis acidic molten salts at temperatures between 500 and 750 °C, distinct MXenes with A elements of Si, Zn, Ga, and Al may be etched from various MAX phases. There is still potential for improving etching techniques for MXene separation for particular devices. Now, we will explain stepwise in detail about various 2D non‐TI MXene NMs production.

### 3.1 Sc‐MXene Synthesis

**Figure 8:**
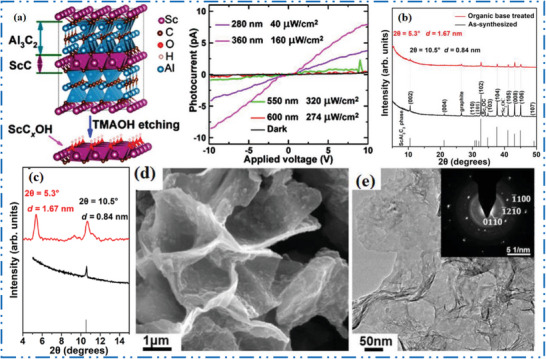
a) Scheme shows ScC*_x_* OH, from ScAl_3_C_3_ MAX etching, and *I*–*V* curves, b) XRD of ScAl_3_C_3_ before/after TMAOH etching, c) full analysis with 2*θ* (5°, 15°), d) SEM after etching at 30–40 °C for 72 h, and e) TEM 2D nanoflakes, with SAED pattern. Reproduced with permission.^[^ ^138^ ^]^ Copyright 2019, American Chemical Society.

Scandium (Sc) has not obtained much attention in synthesis of novel 2D NMs since it is hard to extract in large amounts. The poor market availability and high price of Sc make it difficult to procure material. Applications should be supported by special characteristics or noteworthy performance advantages because comparable MXene NMs’ end products will always be more expensive due to higher price of Sc precursors than Ti‐based 2D MXene NMs. The intention of avoiding expense related to lost material via trial‐and‐error detection methodologies; it is better to identify first detail characteristics of the Sc‐MXene based 2D NMs by theoretical evaluation. To get a ScAl_3_C_3_, the parent MAX may be converted into the 2D hydroxyl‐terminated, carbon‐deficient ScC*_x_* OH (**Figure** **8**).^[^ ^138^ ^]^ Initially, in situ reactive pulsed electric current synthesis technique used to produce a ScAl_3_C_3_ precursor. Subsequently, distinct ScAl_3_C_3_ layers were etched using tetramethylammonium hydroxide (TMAOH) like etchant. Figure 8a,c shows a scheme of ScC*_x_* OH, from ScAl_3_C_3_ MAX and their XRD before/after etching in TMAOH with full analysis between 5° and 15°. Figure 8d shows layer expansion and individual grain exfoliation along basal planes, which indicate 70% transfer of the ScAl_3_C_3_ to the 2D ScC*_x_* NM. Delaminated flexible 2D ScC*_x_* nanoflakes with hexagonal symmetry are clearly seen in the TEM picture and SAED pattern (Figure 8e).

Using a traditional pulsed DC magnetron sputtering technique, a Sc_0.29_Al_0.71_N MXene thin film measuring 780 nm thick was created on a Mo/SiO_2_/AlN/SOI substrate. ScAlN thin‐film was deposited using a standard pulsed DC magnetron sputtering method. Four process chambers (AlN chamber, AlScN chamber, Mo chamber, and preclean chamber) and one transport chamber made up this PVD cluster system. A 12‐in. Sc_0.3_–Al_0.7_ alloy target was installed in the AlScN sputtering chamber. Without vacuum breaking, the ScAlN film was deposited on a 6‐in. Mo/SiO_2_/AlN/SOI substrate. Using the 7500 W power, then a Mo (110) thin film was created keeping the target substrate distance 70 mm at 300 °C as a bottom electrode for electrical property measurements. The SOI wafer is cleaned progressively by argon ion soft etching in the clean chamber to assure clean surfaces film growth. The argon gas pressure during the 20 min sputtering period was 2.6 mTorr. Four 90 °C rotations of the substrate were made during the deposition to provide uniform film thickness. It is important to note that in order to increase the quality of the ScAlN (002) with better crystal orientation employed SiO_2_ and AlN as a seed layer before sputtering the Mo layer. The prepared ScAlN thin film offered high‐quality crystal orientation and a high effective piezoelectric coefficient *d*_33_ of 12.6 pC N^−1^. In addition, there was no wurtzite‐to‐rock‐salt phase transition under high pressure (≤20 GPa), which is quite beneficial for application in strong coupling piezoelectric devices with high‐pressure stability.^[^ ^139^ ^]^

### 3.2 V‐MXene Synthesis

**Figure 9:**
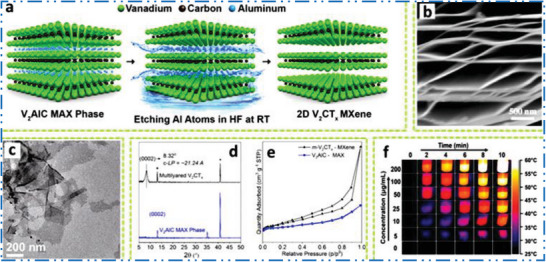
a,b) Scheme shows selective etching of V_2_AlC to synthesis V_2_CT*_x_*, Reproduced with permission.^[^ ^13^ ^]^ Copyright 2017, American Chemical Society, and its SEM, Reproduced with permission.^[^ ^140^ ^]^ Copyright 2016, American Chemical Society. c) TEM of FL V_2_C after sonication. Reproduced with permission.^[^ ^147^ ^]^ Copyright 2021, Springer Nature. d) XRD of V_2_AlC and the m‐V_2_CT*_x_*, e,f) TG/DTA of m‐V_2_CT*_x_* (surface area = 10.25 m^2^ g^−1^) as well as V_2_AlC (surface area = 1.50 m^2^ g^−1^). Reproduced with permission.^[^ ^148^ ^]^ Copyright 2020, American Chemical Society, and thermal photographs of V_2_C NSs irradiated with 808 nm. Reproduced with permission.^[^ ^149^ ^]^ Copyright 2020, Wiley‐VCH.

In comparison to Ti, vanadium (V) is one of the mainly abundant metal exist on earth, making 2D V‐MXene NMs as a cost‐effective one 2D NMs. In contrast to other 2D V‐based MXene, non‐Ti 2D MXene has demonstrated increased research for experimental investigation in various applications, including nuclear waste adsorption,^[^ ^140^ ^]^ pseudocapacitors,^[^ ^141^ ^]^ electrocatalysis,^[^ ^142^, ^143^ ^]^ theranostics,^[^ ^144^ ^]^ etc. The V has also been successfully incorporated into double‐TMs, including Ti‐V, Mo‐V, and Cr‐V based 2D MXene NMs,^[^ ^145^ ^]^ according to various studies.^[^ ^146^ ^]^ The Al atoms are typically removed by HF acid‐based etching in conventional preparation of V_2_CT*_x_* MXene NMs^[^ ^13^ ^]^ from parent V_2_AlC MAX precursors phases (**Figure** **9**a). Figure 9b shows V_2_CT*_x_* with multiple stacks of NSs that sonicated to create FL 2D NMs.^[^ ^147^ ^]^

Figure 9d,e shows how nitrogen physisorption and XRD may be apply to find out the transfer of MAX to 2D MXene based NM.^[^ ^148^ ^]^ There have been information of HF free and harmless production methods employing HCl with LiF^[^ ^150^ ^]^ or NaF that successful produce 2D vanadium carbide of high quality. Tetrabutylammonium hydroxide (TBAOH) and TMAOH are two delaminating agents that may be used to efficiently create vacancies and/or intercalation in a configuration of V‐based MXene NMs. Expanding interlayer gap and creating V–O–Sn covalent bond in vanadium carbide both have been accomplished using a similar ion intercalation approach.^[^ ^151^ ^]^ The 2D V_2_CT*_x_* MXene NMs quality, durable stability, as well as optoelectronic capabilities were all enhanced because of careful control of the 2D MXene NMs’ structure. The production of V_2_C with a high yield of 90% and strong structural integrity has also been examined utilizing green delamination techniques employing algae extraction.^[^ ^149^ ^]^

### 3.3 Cr‐MXene Synthesis

**Figure 10:**
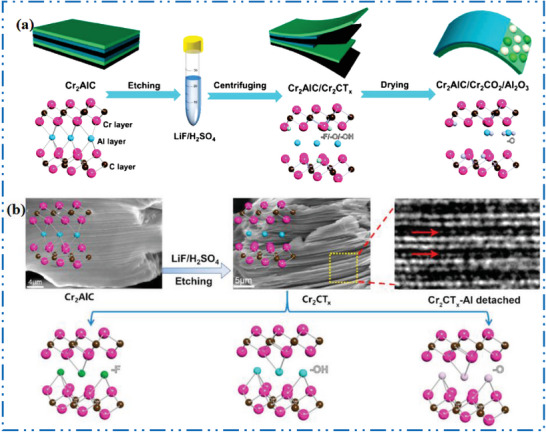
a) Scheme shows Cr_2_AlC crystal structure with its MXene. b) TEM image. Reproduced with permission.^[^ ^158^, ^159^, ^168^ ^]^ Copyright 2020, American Chemical Society.

Intense rivalry with distinguished MXene NMs based on Nb, V, and Mo, Cr‐based MXenes have received little attention. Currently the Cr motivations are receiving a great desirable consideration in the 2D MXene NMs growth due to the increased interest in its ferromagnetic performance,^[^ ^152^ ^]^ H_2_ storage potential,^[^ ^153^ ^]^ as well as electronic properties.^[^ ^154^ ^]^ The most extensively researched MAX phase for Cr‐MXene is often the MAX phase, Cr_2_‐AlC (**Figure** **10**a). Although it has attractive features for nanoelectronics^[^ ^155^ ^]^ and water‐splitting uses.^[^ ^156^ ^]^ The Cr‐based 2D MXene NMs are one of the hardest types of MXene NMs to manufacture experimentally.^[^ ^157^ ^]^ Figure 10a illustrates a graphic version of exfoliation of Cr_2_AlC to Cr_2_CT*_x_*.^[^ ^158^ ^]^ Contrast between Cr (bright) and Al (less‐bright) can be seen in envoy and HAADF‐STEM images of Cr_2_AlC MAX precursor phase (Figure 10b).^[^ ^159^ ^]^ Ball milling,^[^ ^160^ ^]^ the sintering technique,^[^ ^161^ ^]^ electrochemical molten salt production,^[^ ^162^ ^]^ with magnetron‐sputtering (MS) physical vapor deposition techniques^[^ ^163^ ^]^ are further methods that may be used to create the Cr_2_AlC MAX phase. The Cr_2_GaC^[^ ^164^ ^]^ is an option to Cr_2_AlC MAX precursor. There is still a difficulty in synthesizing Cr‐MXene with high purity since the transition into Cr‐MXenes. Although, it was failed and bulk and anisotropic Cr‐carbide was preferred over Cr‐based MXenes.^[^ ^165^ ^]^ It is difficult to synthesize Cr‐MXene, such as Cr_2_C, Cr_2_N, Cr_3_C_2_, Cr_3_N_2_, Cr_4_C_3_, and Cr_4_N_3_,^[^ ^166^ ^]^ which are accounted by low cohesive energy and low stability of Cr‐carbide in comparison to other carbides.^[^ ^45^ ^]^ Another factor is that other Cr‐derived compounds, rather as Cr‐MXene, are created during HF etching.^[^ ^165^ ^]^ The HF acid is used to exfoliate most of the documented MAX precursors into a 2D MXene, although doing so also dissolves metals and creates fluoride salts.^[^ ^166^ ^]^ Combining FeCl_3_ with metal‐complexing agent tartaric acid to create soluble, readily washable complexes of Al and Fe metal cations that employed to overcome the negative reaction of the Cr‐based MAX precursor in F‐based etchants.^[^ ^158^ ^]^ Exfoliation experiments using a combination of acids (KF, HF, NaOH), and studied molten salt method were all failed till date.^[^ ^166^ ^]^ The Cr_2_CT*_x_* was also effectively generated by electrochemically removing Al from MAX precursors using diluted HCl.^[^ ^167^ ^]^

Thin films of Cr_2_GeC and Cr_2‐_ *_x_* Mn*_x_* GeC were deposited by utilizing the magnetron sputtering method. As long as the temperature was above 650 °C, which allowed the reaction to take place, it was found that the deposition rates and heating regimes had no impact on phase formation. Most likely, the development of a thin film of the MAX phase was primarily influenced by the Cr:Ge:C atomic ratios. The Cr_2_GeC MAX phase was more likely to develop when there was a minor surplus of carbon. Significant morphological changes brought about by increasing film thickness resulted in the formation of faceted crystallites. The AFM and XRD data further showed that manganese doping the films caused additional morphological changes and enhanced the phase composition. It was discovered that Cr_2_GeC films with a thickness greater than 40 nm exhibited metallic behavior based on optical spectra, resistivity, and Hall tests. The columnar structure of crystallites may be responsible for the hopping‐type conduction mechanism shown in the Cr_2−_ *_x_* Mn*_x_* GeC film. The concentration of electrons and holes in the well‐synthesized Cr_2_GeC films was calculated to be 10^27^ cm^−1^ using a two‐band model. The carrier mobility of the thin films was ≈200 cm^2^ V^−1^ s^−1^. This carrier mobility was one order of magnitude more than that of bulk polycrystal, demonstrating their superior crystal quality. This thorough investigation of Cr_2−_ *_x_* Mn*_x_* GeC thin films has improved knowledge of MAX phase formation processes and shows potential for the creation of durable conductive and protective coatings.^[^ ^169^ ^]^

### 3.4 Y‐MXene Synthesis

**Figure 11:**
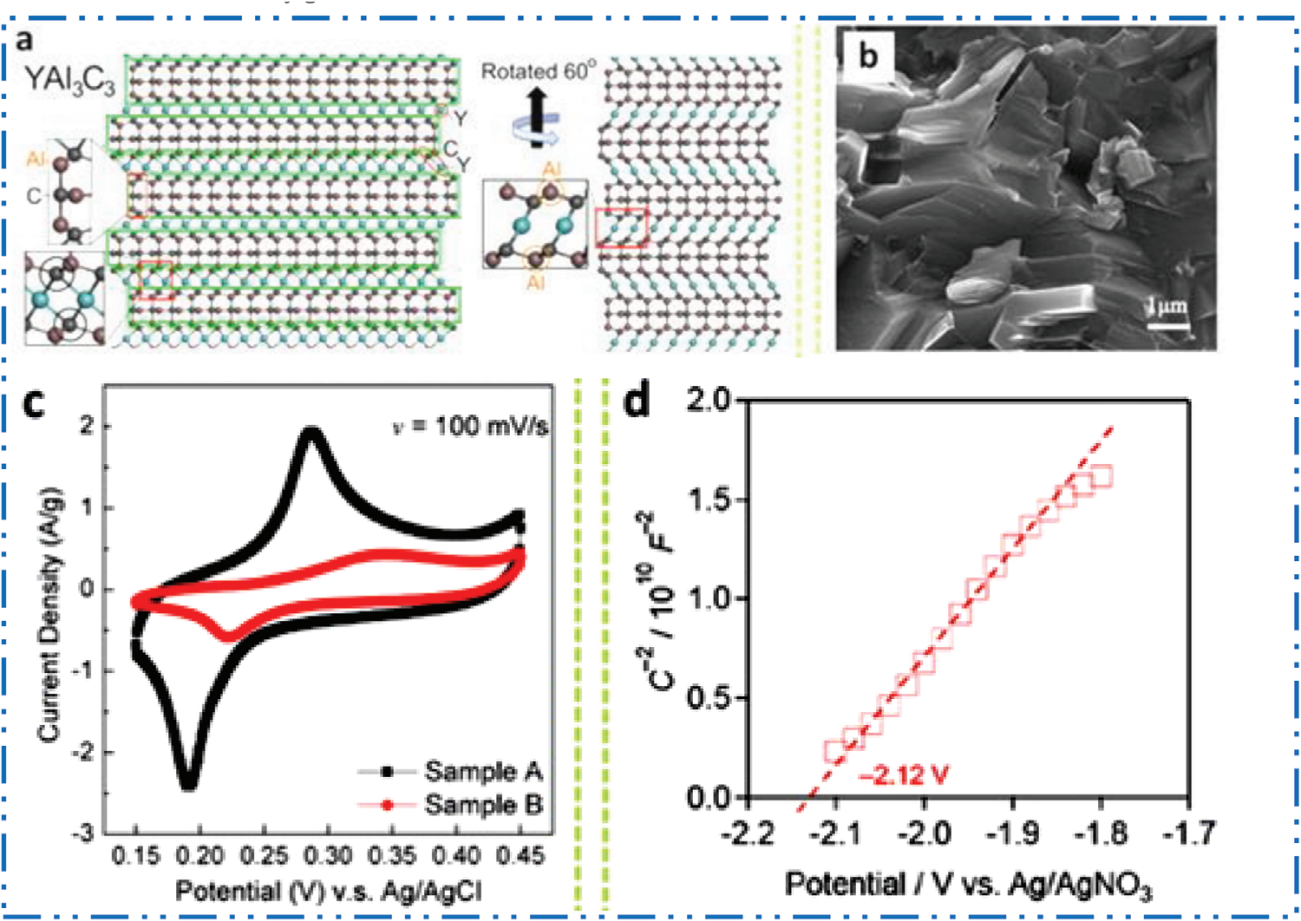
a,b) Scheme shows YAl_3_C_3_ crystal structure with 60° rotation, and its SEM with its crystal structure, Reproduced with permission.^[^ ^170^ ^]^ Copyright 2017, Elsevier. c) CVs of sample A (HF‐etched and DMSO delamination), and sample B (HCl/LiF etching) electrodes in 6 m KOH at 100 mV s^−1^, Reproduced with permission.^[^ ^172^ ^]^ Copyright 2020, Elsevier. d) Mott–Schottky plot of Y_2_CF_2_. Reproduced with permission.^[^ ^173^ ^]^ Copyright 2020, American Chemical Society.

The top‐down method based exfoliation of MAX precursor in the 2D MXene type NM is a common production method for several MXene‐based NMs synthesis. Unfortunately, very few studies have examined Y‐Al‐non‐MAX C's systems. Crystal configuration of YAl_3_C_3_ is comparable to that of layered ternary carbides, which have a common formula T*_m_* Al_3_C_2_^+^ *^m^* (**Figure** **11**a).^[^ ^170^ ^]^ Crystal configuration of these layered ternary carbides may be seen in this configuration as intergrowth configurations made up of two different types of layers: i) Al_3_C_2_ units arranged similarly to Al_4_C_3_, and ii) [T*_m_* C*_m_*] slabs in NaCl kind configuration. The YAl_3_C_3_, contains Y metal atoms sandwiched between Al_3_C_3_, is one arrangement that has been examined (Figure 11a). The Al–C is bound inside the Al_3_C_3_ layer with its effect on how Y atoms interface in the 2D, this configuration looks to be hard to delaminate into 2D NSs having Y atoms. The Y‐atoms share C‐atoms at the top as well as bottom of the crystal lattice in projected image of Figure 11a, creating a Y–C linked network in this 1D direction. When the crystal projection is turned by 60° (Figure 11a) then the Y‐atoms now allocate a top and bottom C–Al atom pair. Chemical etching of Al (orange circle) is anticipated to cause the Y–C to separate into 1D linear chain before 2D MXene NSs. In an experiment, sintering stoichiometric quantities of YC_2_ and Al_4_C_3_ resulted in the formation of YAl_3_C_3_ with additional Y_2_O_3_, Y_4_Al_2_O_9_, and amorphous carbon products. In comparison to the Zr–Al–Si–C and Zr–Al–C, the YAl_3_C_3_ produced improved thermoelectric characteristics. The YH_2_, Al, and C powders can be in situ hot pressed at 1500 °C to create bulk YAl_3_C_3_ ceramic (Figure 11b).^[^ ^170^ ^]^ High purity YAl_3_C_3_ displayed excellent elastic, mechanical, and thermal characteristics. By using high heat treatment based solid‐state processes to build crystals that permitted only F to terminate the layers, bottom‐up synthesis of the Y_2_CF_2_ material was accomplished.^[^ ^171^ ^]^ The Y_2_CF_2_ is a semiconductor by nature, as determined by an investigation of its electronic structure, with a direct bandgap (B.G) of 1.9 eV, indirect B.G of 1.6 eV, and low ionization potential of 3.8 eV. Yet it was discovered that Y_2_CF_2_ is delicate to water and oxygen.

### 3.5 Zr‐MXene Synthesis

**Figure 12:**
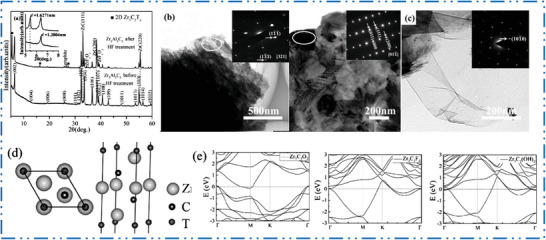
a) XRD of Zr_3_Al_3_C_5_ before/after HF dealing, b,c) TEM of vacuum heat treated Ti_3_C_2_T*_z_* and Zr_3_C_2_T*_z_* after ultrasonication, d) top and side view of Zr_3_C_2_T_2_, and e) energy bands structures. Reproduced with permission.^[^ ^177^ ^]^ Copyright 2016, Wiley‐VCH.

Layered ternary and quaternary compounds make up non‐MAX phases with the typical formulas M*_n_* Al_3_C_2_ and M*_n_* [−Al(Si)]_4_C_3_ (T = Zr or Hf, *n* = 1, 2, or 3…).^[^ ^174^, ^175^, ^176^ ^]^ The Zr–Al–C MAXs precursors are classified as layered ternary and quaternary TMCs, just like Hf–Al–C structures. Al–C units, as opposed to regular Al unit from MAX phases, are preferentially extracted for these non‐MAX phases. In situ reactive pulsed electric current synthesis technique was used to create Zr_3_Al_3_C_5_.^[^ ^177^ ^]^ **Figure** **12**a–c shows that 2D Zr_3_C_2_T*_z_* MXene may be generated by selectively etching Al_3_C_3_ from Zr_3_Al_3_C_5_ using HF acid, as seen by the variations in XRD peaks and its accordion‐like shape. Figure 12d,e shows a top and side view of 2D Zr_3_C_2_T_2_ (T = F, OH, O), and electronic energy bandgaps (B.Gs) of Zr_3_C_2_F_2_, Zr_3_C_2_(OH)_2_, and Zr_3_C_2_O_2_, respectively.^[^ ^177^ ^]^

Using a ZrC sputtering target and radio frequency magnetron sputtering, the 2D MXene ZrC film created on a fused silica substrate. The face‐centered cubic structure and excellent crystallinity of ZrC was confirmed by findings of the XRD examination. The ZrC films produced by magnetron sputtering deposition were exceptionally smooth and uniform, which is advantageous for lowering surface flaws. The surface roughness for these samples were found 7.21 nm.^[^ ^178^ ^]^

### 3.6 Hf‐MXene Synthesis

While M*_n_*_+1_AX*_n_*, an early TMC is the most often reported MAX phase, other layered ternary and quaternary TMCs, such as (MC)*_n_* Al_3_C_2_ and (MC)*_n_* [Al(Si)]_4_C_3_, have M = Zr or Hf and *n* = 1, 2, 3, are also required after as the non‐MAX precursors.^[^ ^174^ ^]^ Predominantly single MAX precursors, based Hf and Zr, have been said to be difficult to synthesize and challenging to create.^[^ ^174^ ^]^ Therefore, hot pressing may be used to create ternary carbides in Hf–Al–C structure, including Hf_3_Al_3_C_5_, Hf_2_Al_4_C_5_, and Hf_3_Al_4_C_6_.^[^ ^179^ ^]^ The elemental distribution of Hf, Al, and C depicts the microsize configuration of Hf:Al:C.^[^ ^180^ ^]^ Zr–Al–Si–C^[^ ^181^ ^]^ and Hf–Al–Si–C^[^ ^182^ ^]^ reports of quaternary carbide have therefore demonstrated success in the preparation of single‐phase 2D MXene NMs precursor ceramic. Anisotropic Hf_2_[Al(Si)]_4_C_5_ particles with a trace quantity of HfC grains are shown to have superior mechanical, thermal, and thermal conductivity characteristics than HfC.^[^ ^182^ ^]^ Two Hf–C layers sandwiched between four Al–C layers (Al_4_C_3_) may be created by combining hot pressing and spark plasma sintering (SPS) procedures to make a Hf_2_Al_4_C_5_ ternary carbide.^[^ ^181^ ^]^ As interfacial connections in M–C and Al–C layers for stacked Hf–Al–C carbides are particularly strong, selectively etching and exfoliating ternary Hf–Al–C carbides must be challenging. This previously shown in Ti_3_(Si_0.75_Al_0.25_)C_2_ system,^[^ ^183^ ^]^ methods to reduce the interfacial adhesion in Hf–C and Al(Si)C sublayers by alloying Si on Al sites allow HF to etch the configuration of Hf_3_[Al(Si)]_4_C_6_ and subsequently delaminate into ML Hf_3_C_2_T*_z_*. XRD findings of Hf_3_[Al(Si)]_4_C_6_ before and after being treated with concentrated HF based solution, showcasing peaks of typical MXene exfoliation from parent MAX phases. The thickness of a bilayer MXene NMs was displayed via delaminated Hf_3_C_2_T*_z_* NSs, matching height profile, and B.G diagram.^[^ ^118^, ^184^, ^186^, ^188^ ^]^

### 3.7 Nb‐MXene Synthesis

**Figure 13:**
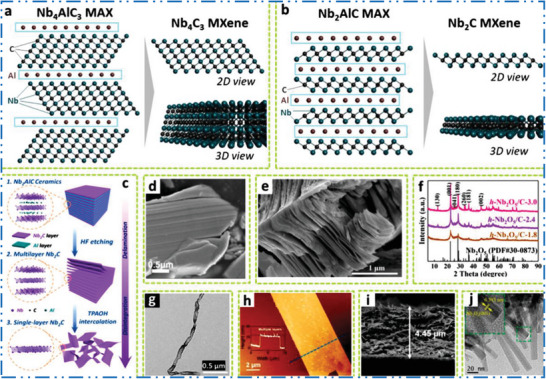
Scheme shows crystal structure of a) Nb_4_AlC_3_ and b) Nb_2_AlC and their MXene. c) Fabrication scheme diagram of Nb_2_C NSs, with HF based delamination and TPAOH intercalation, Reproduced with permission.^[^ ^186^ ^]^ Copyright 2017, American Chemical Society, d) SEM of Nb_2_AlC MAX, Reproduced with permission.^[^ ^187^ ^]^ Copyright 2020, American Chemical Society, e) SEM of HF‐etched Nb_2_C, Reproduced with permission.^[^ ^188^ ^]^ Copyright 2021, American Chemical Society, f) XRD of h‐Nb_2_O_5_/C, Reproduced with permission.^[^ ^190^ ^]^ Copyright 2019, Elsevier, g) TEM of E‐etched Nb_2_CT_*x*_ nanowires, Reproduced with permission.^[^ ^191^ ^]^ Copyright 2020, Wiley‐VCH, h) AFM of M‐KNbO_3_, Reproduced with permission.^[^ ^192^ ^]^ Copyright 2019, Wiley‐VCH, i) SEM of Nb_2_CT_*x*_‐CNT, Reproduced with permission.^[^ ^193^ ^]^ Copyright 2016, Elsevier, j) TEM of N‐Nb_2_O_5_@Nb_2_C composite, Reproduced with permission.^[^ ^194^ ^]^ Copyright 2022, Elsevier.

The uniqueness of its metallic property, outstanding photonic and optoelectronic features, high photothermal stability, biocompatibility, great conductivity, as well as capacity for high charge–discharge rates is only a few of niobium (Nb)‐based MXene NMs are characterized.^[^ ^185^ ^]^ To date, stacked Nb_2_CT*_x_* MXene may be created using three different etching methods: HF etching, LiF + HCl etching, and hydrothermal etching. **Figure** **13**a,b illustrates the typical MAX phases of Nb_4_AlC_3_ and Nb_2_AlC, respectively. Once the Al layers are successfully removed, 2D MXene is produced, giving rise to Nb_4_C_3_ and Nb_2_C. Figure 13c depicts the typical top‐down method of Nb‐based MXene NMs production,^[^ ^186^ ^]^ with Nb_2_AlC serving as the precursor MAX phases (Figure 13d).^[^ ^187^ ^]^ The ML, accordion‐like Nb_2_C structure is then created by selectively etching of Al‐layer by means of HF acid (Figure 13e).^[^ ^188^ ^]^ Nevertheless, HF‐based etched 2D MXene NM demonstrated a propensity to deteriorate even at room temperature.^[^ ^189^ ^]^ Hence, the HF‐free approaches that enable quick and safe production, a wide surface area, outstanding chemical stability, high porosity, high conductivity, and minimal cytotoxicity have also been very successful in producing 2D Nb‐MXenes. TPAOH and TBAOH are often used as intercalants in the delamination process, which is mainly used to create FLs of MXene based NMs. Surprisingly, rising interest in employing MXene is also to create NMs that have catered to the needs of academics. An oxidized form of Nb_2_CT*_x_* MXene has been obtained as Nb_2_O_5_ NPs decorated on surface of carbon layers (Nb_2_O_5_/C) by hydrothermal behavior (Figure 13f) or CO_2_ calcination.^[^ ^190^ ^]^ Another distinct type of niobium carbide is seen in a 1D nanowires (Figure 13g),^[^ ^191^ ^]^ ferroelectric crystals made of 2D MXene (Figure 13h),^[^ ^192^ ^]^ carbon nanotubes (CNTs) and 2D Nb_2_CT*_x_* (Figure 13i),^[^ ^193^ ^]^ and N‐doped niobium carbide‐supported niobium pentoxide (Figure 13j).^[^ ^194^ ^]^

The HF etching approach has a superior etching effect than the LiF + HCl etching method, although it was certified as an effective etchant for Ti_3_C_2_T*_x_*. It may not be suited for Nb_2_CT*_x_* due to the higher bond energy to overcome (Nb–Al). Peng et al.^[^ ^195^ ^]^ employed a hybrid solution of NaBF_4_ and HCl as an etchant in a hydrothermal etching process to synthesize Nb_2_CT*_x_*. With a significantly shorter etching period (20 h), a good exfoliation effect may be obtained. As a result, the hydrothermal etching approach is likely to be extended to additional 2D MXene NMs. A similar HF etching procedure may be used to generate Nb_4_C_3_T*_x_*. By using the magnetron sputtering deposition process, few‐layered 2D Nb_2_C NSs and microfiber‐based 2D Nb_2_C saturable absorber were created. The substantial third‐order nonlinear susceptibility and coexistence of saturable absorption and reverse saturable absorption processes are revealed by Nb_2_C saturable absorber. In comparison to reverse saturable absorption processes, the effective nonlinear absorption coefficient caused by the saturable absorption effect was found to be nonlinear absorption coefficient (β_eff_) ≈−10^5^ GW^−1^, which was two orders of magnitude greater. For the first time, the positive nonlinear refractive index *n*_2_ was calculated to be ≈10^−13^ m^2^ W^−1^. Additionally, square‐wave pulse (SWP) mode‐locked Yb‐doped fiber laser was successfully built using a microfiber‐based 2D Nb_2_C saturable absorber. The single pulse energy can be increased linearly up to 0.89 nJ while the peak power remains fixed, allowing the SWP pulse width to be adjusted from 0.652 to 1.616 ns. According to our findings, 2D Nb_2_C NSs would make a great saturable absorber for investigating saturable dynamics and ultrafast photonics dynamics in fiber lasers.^[^ ^196^ ^]^

### 3.8 Mo‐MXene Synthesis

**Figure 14:**
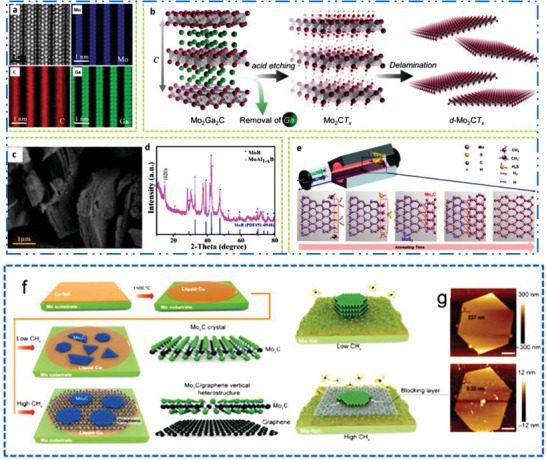
a) HRSTEM and EDX mapping of Mo_2_Ga_2_C, Reproduced with permission.^[^ ^197^ ^]^ Copyright 2019, Wiley, b) Scheme showing synthesis as well as delamination of Mo_2_CT_*x*_, Reproduced with permission.^[^ ^199^ ^]^ Copyright 2016, Wiley, c,d) SEM of MoB MBene and its XRD, Reproduced with permission.^[^ ^201^ ^]^ Copyright 2022, Elsevier, e) Scheme shows Mo_2_C production using heat treatment in CH_4_ as well as H_2_, Reproduced with permission.^[^ ^202^ ^]^ Copyright 2018, American Chemical Society, f,g) scheme shows Mo_2_C crystals synthesis in CH_4_ environment, and its AFM, Reproduced with permission.^[^ ^203^ ^]^ Copyright 2020, Springer.

Effectiveness in the renewable‐energy storage, electrocatalysis, and medicinal uses, molybdenum (Mo) was the next most investigated form of the 2D MXene NM despite its low crustal abundance.^[^ ^197^ ^]^ The conventional basic materials used to produce MXenes are known as MAX phases. Nonetheless, because the thermodynamic conditions are inadequate to generate the MAX precursor of this material, the instability of Mo_2_AlC and Mo_2_GaN is seen. The Mo_2_GaC may be effectively manufactured as layer structure bulk ceramics in its MAX precursor and can be etched to create stable Mo_2_C MXene NMs (**Figure** **14**a).^[^ ^197^ ^]^ Little to no success, attempts to selective etching of Ga from Mo_2_GaC MAX to produce Mo_2_C MXene were made.^[^ ^198^ ^]^ A large‐scale synthesis of Mo_2_CT*_x_* nanoflakes was reported by Halim et al.,^[^ ^199^ ^]^ by selectively etching the Ga from Mo_2_Ga_2_C by means of a LiF/HCl solution (Figure 14b). Afterward, by hand shaking in water, delaminated 2D MXene NMs were easily produced. Unexpectedly, bulk ternary metallic boride, or MAB precursor^[^ ^200^ ^]^ has also demonstrated promise as a basic material for 2D MBene preparation. Boron is used in place of X (C/N) in the first MAX precursor. Similar to the concept and approach used with the 2D MXene NMs, MAB precursors are etched to get 2D TM‐based boride (MBene). Using a hydrothermal‐assisted, HF‐free etching approach, MoB MBene from MoAlB MAB phase was effectively produced (Figure 14c).^[^ ^201^ ^]^

MoAl_1−_ *_x_* B remnants, however, show that Al was not entirely eliminated (Figure 14d). Epitaxial synthesis has been reported to produce MXene in a different non‐MAX phase. For example, MoS_2_ crystals are chemically transformed in Figure 14e by thermal annealing in the existence of a CH_4_ and H_2_ to yield 2D Mo_2_C MXene NSs. The DFT simulations showed successive hydro‐desulfurization and carbide conversion processes took place on MoS_2_’s edge, where conversion method started. The Mo‐MXenes may also be created by bottom‐up techniques, for example, CVD and solid state reactions, which allow for fine control over the surface terminations of such MXenes. Mo_2_C was the first CVD‐grown MXene in 2015. Geng et al.^[^ ^204^ ^]^ established a one‐step CVD approach to manufacture high‐quality Mo_2_C crystals on in situ produced graphene using a Mo–Cu alloy as the catalyst, as illustrated in Figure 14f. Under high CH_4_ flow rates, the quickly growing graphene film acts as a blocking layer, which is critical in controlling the thickness and homogeneity of Mo_2_C hexagons (Figure 14g).

**Figure 15:**
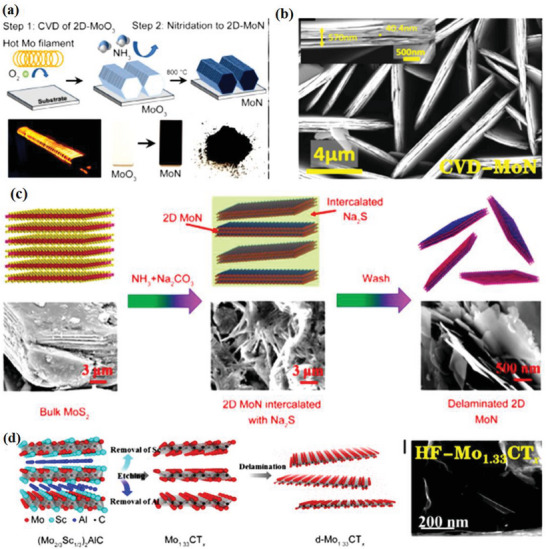
a) HRSTEM and EDX mapping of Mo_2_Ga_2_C, Reproduced with permission.^[^ ^197^ ^]^ Copyright 2019, Wiley, b) Scheme showing synthesis as well as delamination of Mo_2_CT*_x_*, Reproduced with permission.^[^ ^199^ ^]^ Copyright 2016, Wiley, c,d) SEM of MoB MBene and its XRD, Reproduced with permission.^[^ ^201^ ^]^ Copyright 2022, Elsevier.

The 2D MoN is the second CVD‐grown MXene NM. Joshi et al.^[^ ^205^ ^]^ devised a two‐step method to produce MoN (**Figure** **15**a). First, resistively heated Mo filament under O_2_ flowing was utilized to create MoO_3_ on fluorinated tin oxide (FTO) substrate via hot‐filament‐CVD. Following that, the MoO_3_ 2D NSs were annealed in an ammonia environment, resulting in the phase transition from MoO_3_ to MoN. The 2D MoN MXene SEM shows vertically aligned stacks with the basal plane perpendicular to the FTO substrate (Figure 15b). Sun et al.^[^ ^206^ ^]^ produced MoN from MoS_2_ using Na_2_CO_3_‐assisted nitridation (Figure 15c) in an ammonia environment at high temperatures (700–800 °C). Following the washing procedure, high purity MoN NSs may be obtained. CVD procedures, as opposed to top‐down approaches, are devoid of intricate solution reactions and may produce pure 2D MXene NMs without different termination groups, such as O, F, Cl, or OH.^[^ ^204^ ^]^ Notably, the amount of precursors (i.e., carbon or nitride resources) may be precisely regulated, allowing for the determination of the final quality of 2D MXene NMs. The effectiveness of the nitridation technique in producing 2D MoN MXene NM provides solid evidence for the synthesis of additional nitride‐based MXenes. It is also worth mentioning a novel form of Mo‐based MXene (i.e., Mo_1.33_CT*_x_*), the very defective MXene, which was discovered during the etching of (Mo_2/3_Sc_1/3_)_2_AlC. The etching procedure eliminates not only Al layers but also Sc, resulting in Mo_1.33_CT*_x_* MXene with ordered vacancies (Figure 15d).^[^ ^95^ ^]^ Figure 15e depicts typical nanolaminated NSs. This faulty Mo‐based MXene requires a shorter HF etching time of 24 h than Mo_2_CT*_x_*. Using the Mo_2_C sputtering target and radio frequency magnetron sputtering, a thin layer of Mo_2_C was created on a quartz plate substrate. A vacuum pump was used to raise the vacuum level to 6 × 10^−4^ Pa. At 600 °C, the Mo_2_C samples were annealed. Three distinct portions were selected for the measurements in order to gauge the thickness of Mo_2_C coatings. Atomic force microscopy revealed that the Mo_2_C films were between 4.3 and 4.7 nm thick. The uniform surface and thickness of the thin film sample created by radio frequency sputtering are much greater, and the initial transmission is simple to manage.^[^ ^207^ ^]^

### 3.9 Ta‐MXene Synthesis

**Figure 16:**
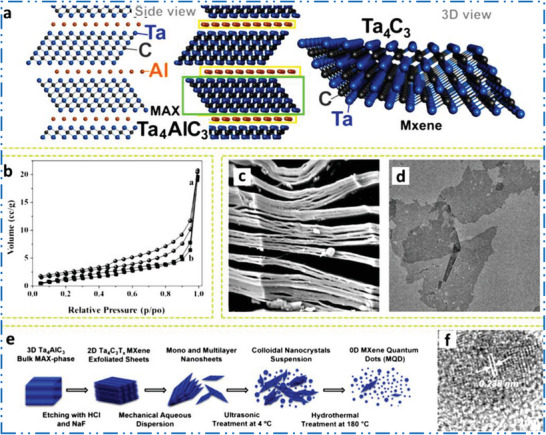
a,b) Scheme shows crystal structure of Ta_4_AlC_3_ and Ta_4_C_3_ and their BET, Reproduced with permission.^[^ ^208^ ^]^ Copyright 2013, Elsevier, c) SEM of multi‐layer Ta_4_C_3_, d) TEM of FL Ta_4_C_3_, Reproduced with permission.^[^ ^209^ ^]^ Copyright 2022, Elsevier, e) scheme on exchange of bulk Ta_4_AlC_3_ to Ta_4_C_3_T*_x_* MQDs, f) HRTEM of ML Ta_4_C_3_T*_x_* NSs discovered definite and exfoliated crystals with lattice d‐spacing of ∼0.260 nm, Reproduced with permission.^[^ ^210^ ^]^ Copyright 2021, Wiley.

One of the metals with the fewest unfavorable biological reactions is tantalum (Ta), with Ta‐derived 2D NMs.^[^ ^211^ ^]^ The Ta‐based NMs, like 2D MXene, possess adjustable characteristics as a result, making them interesting candidates for biological applications. Typical MAX phase arrangement may be observed in the schematic illustration of Ta_4_AlC_3_ MAX precursor's crystal configuration (**Figure** **16**a). When Al layers are take out from MAX precursor, Ta‐based 2D MXene NM is created, expanding similar to accordion configuration and increasing its surface area (Figure 16b).^[^ ^208^ ^]^ Figure 16d depicts a typical multilayered Ta_4_C_3_ accordion‐like structure with FLs (Figure 16c).^[^ ^209^ ^]^ At a particle size of below 5 nm, finer Ta‐MXene NMs were created as MXenes quantum dots (MQDs) (Figure 16e,f).^[^ ^210^ ^]^ The synthesized Ta_4_C_3_T*_x_* MQDs exhibited good biocompatibility, stable suspension, high surface area, and great thermal stability.

### 3.10 W‐MXene Synthesis

**Figure 17:**
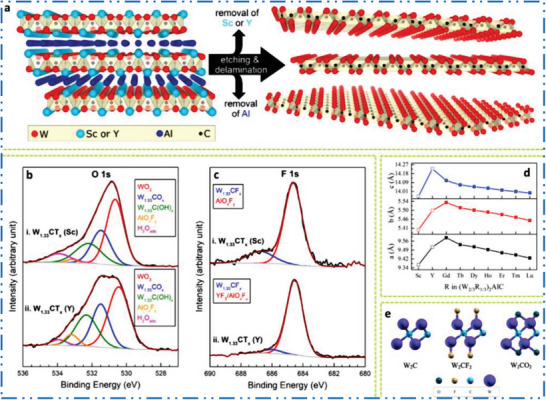
a) Scheme shows in‐plane chemical ordering in (W_2/3_Sc_1/3_)_2_AlC or (W_2/3_Y_1/3_)_2_AlC i‐ (left panel), cause W_1.33_C by controlled divacancies after etching as well as delamination (right panel), Reproduced with permission.^[^ ^97^ ^]^ Copyright 2018, Wiley‐VCH. XPS with curve fitting of (i) W_1.33_CT*_x_* (Sc) and (ii) W_1.33_CT*_x_* (Y) free‐standing delaminated films for b) O1s and c) F1s, Reproduced with permission.^[^ ^216^ ^]^ Copyright 2019, American Chemical Society, d) summary with evaluation of element‐dependent lattice parameters of (W_2/3_R_1/3_)_2_AlC (R = Gd, Tb, Dy, Ho, Er, Tm, Lu) (closed symbols) compared to Ref. [220] (R = Sc, Y) (open symbols), Reproduced with permission.^[^ ^217^ ^]^ Copyright 2021, Elsevier, e) Side view of optimized W_2_C, W_2_CF_2_, and W_2_CO_2_ structural unit‐cell, Reproduced with permission.^[^ ^218^ ^]^ Copyright 2022, Elsevier.

MAX precursors are employed as the primary precursor material for the production of MXene NMs, and it is already stated in the earlier section of this paper. Nevertheless, it was studied that it is challenging to synthesize W‐containing MAX precursors.^[^ ^45^ ^]^ Notably, 17 elemental compositions comprising W‐based M_2_AX phases, for instance, W_2_SC, W_2_AlN, W_2_SN, W_2_SiN, W_2_SnN, W_2_InN, W_2_GeN, W_2_GaN, and W_2_PbC, and other MAX phases, such as Cr_2_SN, V_2_SN, Mo_2_SiN, Mo_2_SC, Mo_2_GeN, Mo_2_InN, Mo_2_AlN, as well as Mo_2_SnN have demonstrated inclination to create unstable phases.^[^ ^212^ ^]^ Hence, rather than a typical MAX phase where only one TM is added, quaternary i‐MAX hybrid phases may be constructed to have a common equation of (M[1]_2/3_M[2]_1/3_)_2_AlC. By deliberately including M2 in a 1:2 ratio into the M1‐dominated layer, alloying MAX precursor phase in this situation can produce in‐plane chemically ordered configurations.^[^ ^213^ ^]^ Stability of i‐MAX hybrid structures may be anticipated when first‐principles computations based on DFT are used, which opens up the prospect of producing i‐MAX precursor experimentally.^[^ ^95^, ^214^ ^]^ Reliant on etching circumstances, such as i‐MAX can produce either (M1_2/3_M2_1/3_)_2_C or M1_1.33_C as 2D MXene NMs products.^[^ ^97^ ^]^ Exploration for i‐MAX was extended to rare‐earth (RE) elements, for example, Pr, Ce, Nd, Gd, Sm, Tb, Ho, Dy, Tm, Er, and Lu, in (Mo_2/3_RE_1/3_)_2_AlC hybrid phase,^[^ ^215^ ^]^ increasing the moiety of MAX precursor into new dimension. Computational approaches have served as theoretical guides in this expansion. By DFT study of more than 18 compositions of (W_2/3_M2_1/3_)_2_AC, where M2 = Sc, Y (W), and A = Al, Si, Ga, Ge, In, and Sn, Meshkian^[^ ^97^ ^]^ expected a phase stability. For experimental synthesis, the quaternary i‐MAX (W_2/3_Sc_1/3_)_2_AlC and (W_2/3_Y_1/3_)_2_AlC phases were determined to be the very stable (**Figure** **17**a). Sc and Y were effectively removed using HF acid or a solution of LiF/HCl, afterward TBAOH, producing 2D W_1.33_C with ordered divacancies. Different quantities of surface terminations were depicted in the fitted XPS peaks for O1s (Figure 17b) and F1s (Figure 17c) for i) W_1.33_‐CT*_x_* (Sc) and ii) W_1.33_CT*_x_* (Y).^[^ ^216^ ^]^ The feasibility of filling the M2 positions of i‐MAX (Figure 17d) with lanthanide elements (Gd, Tb, Dy, Ho, Er, Tm, and Lu) in place of Sc/Y was also examined.^[^ ^217^ ^]^ In a nutshell, although rare‐earth metals “R” = Lu behaved paramagnetically, R = Gd exhibited linear antiferromagnetic characteristics.

### 3.11 Double Transition Metal MXenes Synthesis

The idea of alloying has been widely used to incorporate useful metals and enhance the characteristics of original NMs. According to analysis of the Cr–Ti–Al–C^[^ ^101^ ^]^ as well as Mo–Ti–Al–C^[^ ^219^ ^]^ structures, metal alloying on initial MAX precursors is a widespread practice. Other techniques, like as microwave heating, SPS, and the arc‐melting approach have also been effective in doping MAX phases. Nonetheless, introducing secondary precursor metals by elemental replacement in recognized crystal configurations is the normal standard in the formation of multiple metal MAX phases.^[^ ^105^, ^220^ ^]^ The (Cr_2/3_Ti_1/3_)_3_AlC_2_ is a quaternary MAX precursor relied on M3‐AX2 lattice where two M elements (Cr:Ti) generated an o‐MAX out‐of‐plane ordering phase that is now referred to as the o‐MAX precursor.^[^ ^221^ ^]^ In the meanwhile, M2‐AX phase revealed the in‐plane chemical arrangement in M elements with the same 2:1 ratio, thus the name i‐MAX phase.^[^ ^95^ ^]^ Etching of i‐MAX precursors yields 2D NMs with ordered divacancies as opposed to o‐MAX precursor, where the structure is retained in the subsequent MXene NMs. Further investigations of metal combinations inside as well as outside of IIIB–VIB family were exhaustively looked for after the current finding of recently studied MAX precursors. Thermodynamic stability of M_5_AlC_4_ MAX precursors with M04 M00 AlC_4_ compositions was predicted using DFT calculations (M0 and M00 were Zr, Hf, Ta, Ti, V, Nb, Mo, Sc, and W). According to Deysher and group mates analysis's of the precise formation energy, compositions based on Hf, Zr, and Ta should form with more stability than compositions comprising W and Mo. It is interesting to note that unconventional metals have also had achievement creating MAX with greater magnetic characteristics. According to a systematic theoretical research of alloying Cr_2_AC (A = Al, Ge) with Mn, the resulting phases have varied magnetic orderings based on the Cr/Mn ratio as well as internal ordering. Another work effectively formulated for Mn and Fe substituted in place of Cr_2_AlC utilizing microwave heating and SPS.^[^ ^38^, ^228^ ^]^

### 3.12 Non‐IIIB to VIB Metal Group Synthesis

Majority of known 2D MXene NMs employed M as an early TM from group‐(III–VI)B. Notably, only MXene members have excellent B.Gs for semiconducting qualities such as Sc_2_C(OH)_2_ (IIIB), Y_2_C(OH)_2_ (IIIB), and Cr_2_TiC_2_(OH)_2_.^[^ ^155^, ^223^, ^224^ ^]^ Lutetium (Lu) is a family of group‐IIIB lanthanide series that has recently demonstrated potential benefits like Lu‐MXene and Lu_2_C. Lu‐MXene NMs with fluorine and hydroxyl terminated topologies are discovered to be useful as semiconductors in vis, and NIR optical devices.^[^ ^225^ ^]^ Ingason et al.^[^ ^226^ ^]^ impulse is to investigate stable but uncharted MAX phases led to the theoretical prediction and fabrication of Mn_2_GaC. By adopting heteroepitaxial thin film production, Mn effectively inserted in a MAX precursor, is showing the homogenous elemental layers and atomic arrangement of Mn, Ga, and C. In next section we will explain the theory and experimental demonstration of MXene general properties.^[^ ^37^ ^]^

## 4 Theory and Experimental Demonstration of Non‐Ti MXene General Properties

### 4.1 Electronic Properties

**Figure 18:**
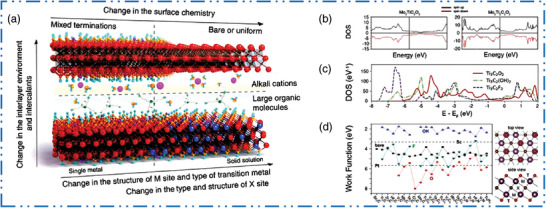
a) Scheme shows of different compositional and structural factors showing electronic as well as optical properties of MXene NMs, b) DOS, illustrating the result of MXene configuration, c) DOS shows a cause of surface chemistry on electronic properties of MXene NMs, and D) reliance of a work function of MXene NMs on their surface chemistry. Reproduced with permission.^[^ ^34^ ^]^ Copyright 2021, American Association for the Advancement of Science (AAAS).

MXene composition, structure, surface chemistry, with interlayer chemistry all affect their physical and (electro)chemical features (**Figure** **18**a). The majority of 2D MXene NMs that behave like metals in terms of electron transport, with linearly decreasing its resistivity with temperature (d*R*/d*T* > 0).^[^ ^125^, ^227^ ^]^ Changing the TM type and M site's structure in MXene NMs can lead to a negative temperature dependency of resistivity (d*R*/d*T* < 0), giving rise to MXene NMs that behave like semiconductors.^[^ ^121^ ^]^ These later include Mo_2_CT*_x_*, Nb_2_CT*_x_*, and V_2_CT*_x_* as examples.^[^ ^199^ ^]^ Additionally, the 2D Mo_2_TiC_2_T*_x_* o‐MXene NMs exhibited semiconductor‐like behavior.^[^ ^228^ ^]^ A sample of how the M‐site structure might alter DOS of MXene NMs is shown in Figure 18b. It is projected that all 2D MXene NMs without surface terminations functioned as metallic conductors with carriers made of free TM electrons.^[^ ^227^ ^]^ Surface terminations, in contrast to ordinary metals, can alter DOS and shift Fermi level, make them electrically adjustable (Figure 18c).^[^ ^229^ ^]^ A switch from metallic to semiconducting B.G opening, and a wide range in their work functions have all been anticipated for various MXenes other than Ti_3_C_2_T*_x_* (Figure 18d).^[^ ^230^ ^]^

Nb_2_CT*_x_* (T*_x_* = Se, S, or NH) has shown superconductivity, on the other hand, nonterminated or O‐based terminated ML Nb_2_C did not show a superconducting transition.^[^ ^39^ ^]^ Thermoelectric characteristics and heat transport are similarly impacted by MXene surface terminations.^[^ ^231^ ^]^ Electrical properties of ML and films are also impacted by the intercalants that are frequently utilized for delamination.^[^ ^121^ ^]^ Tetrabutylammonium (TBA^+^) and other large organic cations are intercalated in the 2D MXene‐based nanoflakes, resulting in a significant interlayer gap.^[^ ^232^ ^]^ This separation prevents interflake electron hopping; make the resulting films less conductive. By contrast, the high conductivity of MXenes and narrow interlayer distance are often maintained by intercalated alkali cations.^[^ ^233^ ^]^ For example, Mo_2_TiC_2_T*_x_* underwent a change from band semiconducting (−d*R*/d*T*) to metallic performance as a result of the deintercalation of water and TBA^+^.^[^ ^121^ ^]^ Additionally influencing a physical characteristics of 2D MXene NMs and their chemical stability are a flake size, stoichiometry, surface chemistry, and point defects, majority of which are vacancies in M and C sublattices that are either inherited from the precursor or produced by etching.^[^ ^234^ ^]^ Greater intraflake electron transport and chemical stability are demonstrated by larger, defect‐free 2D MXenes flakes, and the conductivity of these materials can exceed 20 000 S cm^−1^.^[^ ^74^ ^]^

### 4.2 Magnetic Properties

Magnetic characteristics of 2D MXene NMs are also intriguing based on different investigations techniques. Therefore, MXenes with desirable magnetic characteristics, especially of antiferromagnetic nature, were expected to be possible by tuning M site of o‐MXene NMs via altering the outer‐layer TMs (M″).^[^ ^146^ ^]^ For o‐MXene NMs with Mn in intermediate layer and Mn nitrides, ferromagnetism was anticipated.^[^ ^146^ ^]^ At about 30 to 35 K, a magnetic glass transition in Cr_2_TiC_2_T*_x_* was examined.^[^ ^235^ ^]^ Theoretical forecasts continue to offer direction for prospective compositions and structures. As, in definite simulated o‐MXene NMs, the surface M′ layers are under octahedral crystal force fields while a central M″ layers are not spin‐polarized, causing band splitting in their 3D orbitals moreover, so, a net spin moment showing magnetic order of o‐MXene NM.^[^ ^146^ ^]^ Liu et al.^[^ ^236^ ^]^ developed a crystal field theory‐based model to calculate a magnetic property of MXene NMs, as well as findings demonstrated that Mn_2_NT*_x_* acts as a highly ferromagnetic material at ground state, though the magnetic nature disappeared when surface groups are present. The 2D Cr_2_C, Cr_2_N, Ta_3_C_2_, and Cr_3_C_2_ MXene NMs are regarded as a ferromagnetic in nature that can be stripped from their MAX phase.^[^ ^237^, ^238^ ^]^ Single layer Mn_2_C MXenes are antiferromagnetic with high Neel temperature of 720 K, according to theoretical estimates.^[^ ^239^ ^]^ Surface functionalization with F, Cl, and OH, converts it to a ferromagnetic state with a high Curie temperature (520 K).^[^ ^240^ ^]^ A few MXene NMs, such as Cr_3_C_2_ and Mn_2_C, are just theoretically predicted and have not been synthesized experimentally till date. He et al.^[^ ^240^ ^]^ described a magnetic characteristics of the 2D MXene NMs (T = F, Cl, OH, O, and H) based on a theoretical analysis. MXenes containing functional groups having formal charges (F, Cl, and OH) preserve a ferromagnetic ground state following functionalization, but other MXenes turned into nonmagnetic as they are symmetrically functionalized on their surface. Crystal strain has significant impact on crystal magnetism, according to theoretical investigations. Ion doping and vacancy introduction were proposed as two key ways for controlling its magnetic characteristics.^[^ ^241^ ^]^

### 4.3 Optical Properties

**Figure 19:**
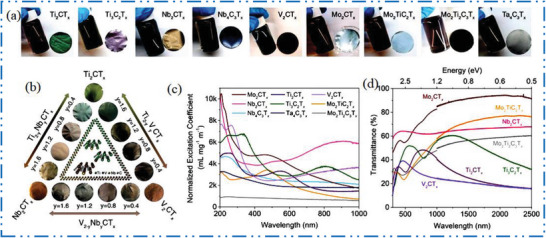
a) Color of colloidal solutions of a range of MXene NMs and their analogous freestanding films. b) Photographs of three M′_2‐_ *_y_* M″*_y_* CT*_x_* solid solution schemes, c) UV–vis–NIR optical extinction properties of aqueous dispersions of various TMCs, d) UV–vis–NIR transmittance spectra from 300 to 2500 nm for MXene thin films. Reproduced with permission.^[^ ^34^ ^]^ Copyright 2021, American Association for the Advancement of Science (AAAS).

In a vis and NIR range, MXene NMs demonstrated longitudinal and transversal surface plasmon types; it is demonstrated that transverse plasmons are independent of the lateral dimension of the nanoflakes.^[^ ^242^ ^]^ As interband transitions, they also reveal high UV‐absorption. Types as well as configuration of M and X sites, and stoichiometry of surface T*_x_*, all affect the optical characteristics of MXenes. **Figure** **19**a,b demonstrates that certain MXenes contain plasmonic peaks that span the whole vis–NIR spectrum area^[^ ^243^ ^]^ and plasmonic hues in transmission (colloidal solution or thin film) and reflection (solid‐state ML films).^[^ ^125^ ^]^ Generally, significant excitation peaks in MXenes' optical spectra go to high energies when their *n*‐value is reduced.^[^ ^243^ ^]^ In comparison, V_2_CT*_x_* has a greenish blue color in transmission and a brownish gold color in reflection, with no absorption peak in UV–vis–NIR band area.^[^ ^244^ ^]^ The V_2_CT*_x_* has shown a larger level of value.^[^ ^245^ ^]^ Figure 19c illustrates how different compositions of the (Ti, Nb)_2_CT*_x_* and (Ti, V)_2_CT*_x_* structures exhibit varied plasmonic hues as a consequence of changing the M′:M″ ratio in solid solution MXene NMs.^[^ ^110^, ^246^ ^]^ Figure 19d shows UV–vis–NIR transmittance spectra from 300 to 2500 nm for MXene thin films.^[^ ^34^ ^]^

### 4.4 Mechanical Properties

**Figure 20:**
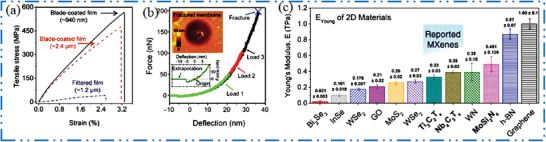
a) Tensile stress versus strain curves of Ti_3_C_2_T*_x_* films with different thickness formed by vacuum‐assisted filtration and blade coating. b) Force–deflection curves of a bilayer Ti_3_C_2_T*_x_* nanoflake at different loads. c) Comparison of the effective Young's moduli. Reproduced with permission.^[^ ^34^ ^]^ Copyright 2021, American Association for the Advancement of Science (AAAS).

Although early analysis indicated that MXenes would have a high elastic modulus like bulk carbides^[^ ^247^ ^]^ and experimental study of the 2D MXene NMs mechanical properties are only now at beginning.^[^ ^248^ ^]^ The SL flakes of Nb_4_C_3_T*_x_* MXene NMs were experimentally examined in terms of fundamental mechanical characteristics using AFM nanoindentation.^[^ ^249^ ^]^ As a result subsistence of point defects with T*_x_*, a studied modulus of SL Nb_4_C_3_T*_x_* only reached 0.39 TPa,^[^ ^250^ ^]^ which is still below theoretically supposed limit.^[^ ^251^ ^]^ The Nb_4_C_3_T*_x_* has a breaking strength of 26 ± 1.6 GPa. **Figure** **20**a,b shows a tensile stress versus strain curves of the Ti_3_C_2_T*_x_* MXenes based thin films with different thickness formed by vacuum‐assisted filtration and blade coating, and force–deflection curves of a bilayer 2D Ti_3_C_2_T*_x_* nanoflakes at different loads. Figure 20c shows the comparison of the effective Young's moduli.^[^ ^34^ ^]^ The published Young's modulus values of numerous 2D NMs,^[^ ^250^ ^]^ incorporated into the recently found vapor‐grown MoSi_2_N_4_ material, are compared in Figure 20c.^[^ ^252^ ^]^ The in‐plane Young's modulus of nitride‐technique based MXene NMs is projected to be superior than that of carbide MXene NMs.^[^ ^253^ ^]^

### 4.5 Topological Insulator Properties

**Figure 21:**
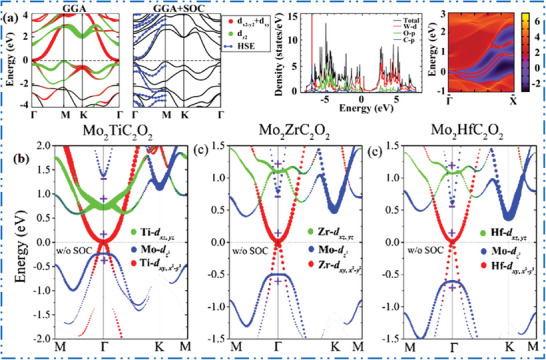
Band structures of a) W_2_CO_2_, Reproduced with permission.^[^ ^254^ ^]^ Copyright 2015, American Physical Society, Cornell University Press, b) without/with SOC, and respected edge states, Reproduced with permission.^[^ ^255^ ^]^ Copyright 2016, American Chemical Society.

Despite the fact that some functionalized 2D MXene NMs were anticipated to have a 2D topological insulator (TI) nature, in which electrons travel alongside an edge states, allowing in dissipation‐free transport appropriate for low‐power electronic devices. Relativistic spin–orbital coupling (SOC) can cause 2D TI phase, which has a substantial impact on electronic configuration of NMs containing heavy elements. The Mo, W, Ti, Zr, and Hf‐atoms make up the majority of observed TIs MXenes. For instance, O‐functionalized M_2_CO_2_ (M = W, Mo, and Cr) are 2D TIs with significant SOC‐induced B.Gs, with W_2_CO_2_ computed using the GGA (HSE06) functional reaching 0.194 (0.472) eV (**Figure** **21**a).

MLs phases and external disturbances not disrupt the 2D MXene NMs band structure, which has been proven. Si et al. as well as Khazaei independently studied the occurrence of the 2D TI phase in MXenes with double TM elements, like Mo_2_MC_2_O_2_ (M = Zr, Ti, or Hf) (Figure 21b), whereas Khazaei also explored W_2_MC_2_O_2_.^[^ ^255^, ^256^ ^]^ The Mo_2_MC_2_O_2_ has huge gaps of 0.1–0.2 eV depending on the “M”‐atoms, while W_2_HfC_2_O_2_ is projected to have the greatest B.G of 0.285 eV (0.409 eV at the HSE06 level). In Mo_2_M_2_C_3_O_2_ (M = Ti, Zr, or Hf), the quantum spin Hall phase was verified, with a range of M‐atoms contributing to SOC induced B.G of 38–152 meV.^[^ ^257^ ^]^ Recently, Liang et al. discovered that nitride type Zr_3_N_2_F_2_ and Hf_3_N_2_F_2_ MXene are transformed into TI by applying a lattice strain.^[^ ^258^ ^]^ As transfer of charge, substitution doping, and proximity induced effects in Sc_2_C(OH)_2_ MXene make it a novel form of s‐pd band inversion TIs that enters into the TIs phase when an electric field is applied. The almost free electron states revealed by parabolic energy dispersion in the Sc_2_C(OH)_2_ are suitable surface transport channels accountable for strong carrier mobility. Such states are near and above Fermi level, with no partial contributions from any elements. The above materials were previously discovered in early tests with mixed surface T*_x_*, and one of the recommended approaches for practically constructing TIs MXenes is careful regulation of surface T*_x_* of oxygen terminated Mo_2_CT*_x_* during synthesis.^[^ ^259^ ^]^ In next section we will explain in detail about each non‐Ti MXenes NMs properties individually.

### 4.6 High‐Entropy 2D MXene NMs

First‐principles calculations show the significance of high configurational entropy in equimolar multielements in producing pure and stable phases of multiprinciple elements (MPEs) MAX phases and link the synthesizability of quaternary high‐entropy MAX to an entropy‐driven stabilization. The efficient synthesis of MAX and MXene materials with high entropy by maximizing the configurational entropy to stabilize (near) equimolar mixtures in a manner similar to that in other disordered multicomponent systems (e.g., bulk ceramics and metals). The increasing family of MXenes now has a broad compositional space that may be investigated for uses such as energy storage, catalysis, and microstructural stability in harsh settings. This exotic subgroup of high entropy MPE MXenes was successfully synthesized. Furthermore, future modifications to the electrochemical, catalytic, electrical, and magnetic characteristics of MXenes may greatly benefit from knowledge of the control over the composition of each transition metal layer. The development of topological insulator MXenes have not yet been synthesized but are predicted in theoretical studies,^[^ ^260^, ^261^ ^]^ may be further made possible by a particular choice of multiple constituent transition metals. This is due to higher degrees of freedom in component selectivity and phase formation when using more than one transition metal in MXenes. The vast compositional space and the potential for entropic stabilization in the high‐entropy MAX and MXene phases present a variety of computational difficulties and opportunities.

The quantitative calculation of entropy, prediction of thermochemical properties, transition temperatures, and investigation of their transport properties are some intriguing questions that can be addressed by combining traditional modeling techniques with recent developments in big data analytics. This will help us better understand the mechanisms affecting the mechanisms affecting their synthesizability. With the use of molecular dynamics, which can illuminate the diffusion rates in disordered materials, the kinetic features may be investigated. A useful source of information on the durations and stability of metastable phases under various operating conditions can be found in recent data‐driven methodologies. Overall, the enormous amount of synthetic and experimental data sets that will be produced offers enormous opportunities for artificial intelligence and machine learning to pinpoint crucial trends for quickening the process of materials design and discovery in this significant class of high‐entropy 2D materials.

**Figure 22:**
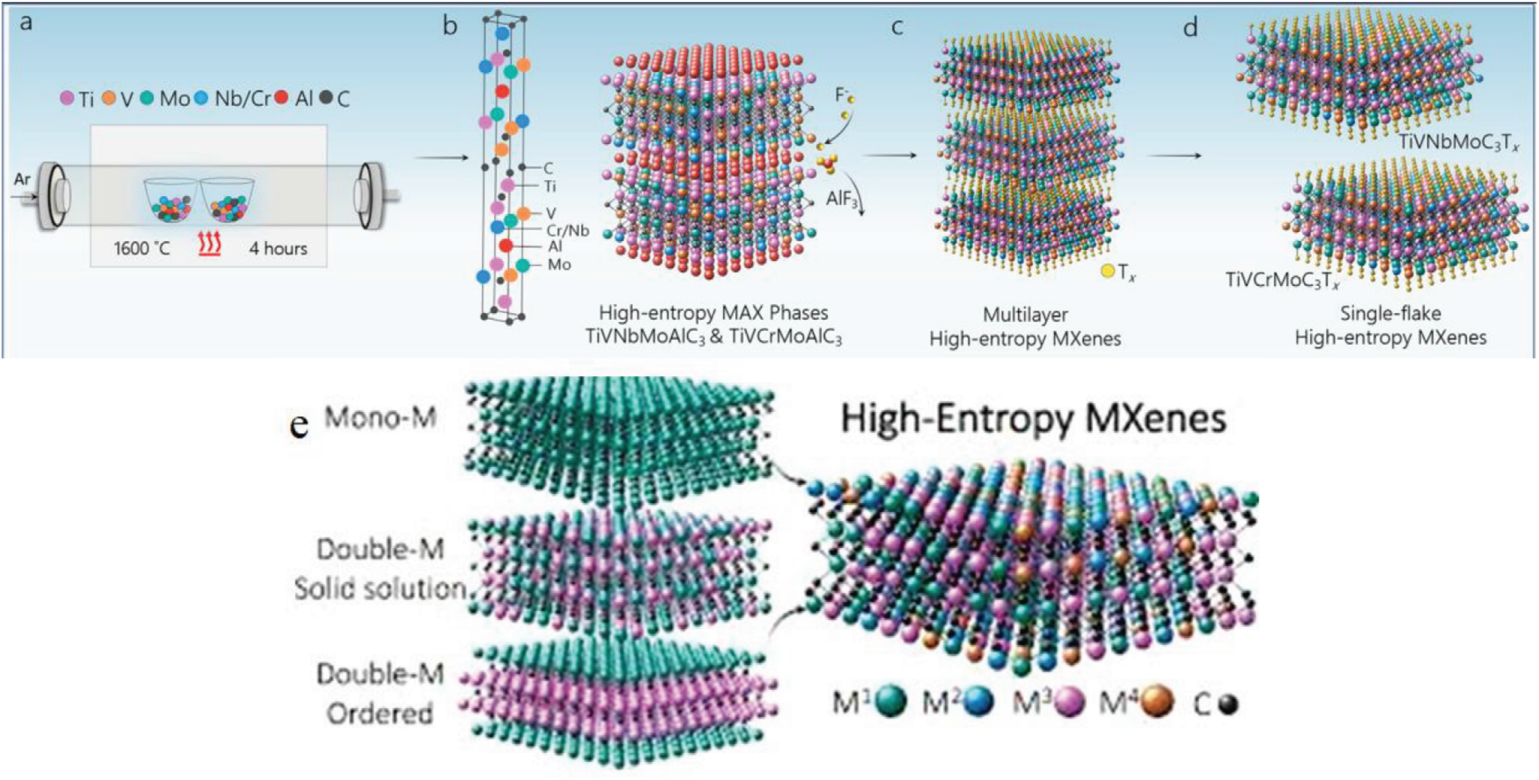
Schematic for the synthesis of high‐entropy MAX and MXenes. a) Reactive sintering of high‐entropy MAX phases. b) MAX phase unit cell (left) of M^1^ M^2^ M^3^ M^4^ AlC_3_ with elements Ti (pink), V (orange), Nb or Cr (blue), Mo (green), Al (red), and C (black). c) Selective etching of Al layer by HF to synthesize multilayer high‐entropy MXenes. d) Delamination of multilayer MXenes is completed via organic molecule intercalants, which leads to the formation of single flakes of high‐entropy MXenes VNbMoC_3_T*_x_* and TiVCrMoC_3_T*_x_*. e) High‐entropy MXenes. Reproduced with permission.^[^ ^34^ ^]^ Copyright 2021, American Association for the Advancement of Science (AAAS).

Investigate the formation enthalpy (*H*) of MAX compositions with reference to the combination of the most competitive phases, *H*_cp_ = *H*_MAX_ phase – *H*_competitive phases_, in order to comprehend the synthesizability of high‐entropy MAX and MXene phases and to quantify the thermodynamic stability of MAX compositions. The total energies of the phase structures and the total energies (per atom) of the bulk phases of M, A, and X elements should be used to compute the formation enthalpy of each phase. Then, a linear optimization procedure was used to determine which stage was the most competitive.^[^ ^262^ ^]^ The relative stability of MAX phases in relation to the competitive phases is indicated by the negative *H*_cp_. The likelihood of their experimental realization is often higher the more negative the enthalpy value. Configurational entropy, or statistically the number of discrete representative positions of the alloy constituents, which depends on the number of elements in the system under discussion, is a significant factor in the stabilization of multicomponent systems. For three and four transition‐metal (three‐M) MAX systems, the entropic contribution can be estimated using^[^ ^263^ ^]^

(1) $$\Delta S_{\mathrm{mix}}=-R\sum_{\left(i=1\right)}^{k}\left(\mathrm{xilnxi}\right)$$

where xi is the mole fraction of the *i*th component in a system with *k* total components.^[^ ^264^ ^]^ The calculated entropic contribution at 1600 °C for three‐M and four‐M systems is −0.1773 and −0.2238 eV f.u.^−1^, respectively. Based on configurational entropy, the four‐M MAX phase has more favorable entropy to form as a single phase compared to the three‐M one (**Figure** **22**). The entropic stabilization explains the preference for four‐M single‐phase MAX phase as compared to three‐M phases of MAX under similar synthesis conditions. Note, however, that our enthalpy calculations reveal that the three‐M MAX is also synthesizable. Since we only used one synthesis temperature and duration (1600 °C, 4 h) for the sake of consistency, we cannot rule out the single‐phase formation of the three‐M MAX by further annealing at a desired temperature and duration. It is known that a lower contribution of configurational entropy can lead to the formation of a multiphase system (undesired phases) in the absence of post annealing treatment.^[^ ^25^ ^]^ Further detailed studies on the nature of competing phases and formation pathways are required to understand the trends in synthesizability of high entropy MAX phases.

Two high‐entropy TiVNbMoAlC_3_ and TiVCrMoAlC_3_ MAX phases were successfully synthesized by Nemani et al.,^[^ ^265^ ^]^ and converted to high‐entropy TiVNbMoC_3_T*_x_* and TiVCrMoC_3_T*_x_* MXenes with an equimolar percentage of Ti:V:Nb:Mo and Ti:V:Cr:Mo major transition metals. These high entropy MAX phases were created via conventional pressureless reactive sintering, and their corresponding single‐ to few‐layer MXenes were created by selective etching with hydrofluoric acid and tetramethylammonium hydroxide delamination. XRD, SEM, and STEM were used to confirm the effective synthesis of single‐to few layer high‐entropy MXenes from high‐entropy MAX phases and their purity. Additionally, using XPS and EDS in SEM, researchers were able to confirm the bonding properties in high entropy MXenes and pinpoint the equimolar composition of transition metals. The elemental mapping using EDS in STEM was used to determine the equimolar distribution of transition metals in the transition metal layers in the delaminated single‐to‐few layer MXenes. First‐principles calculations show the significance of high configurational entropy in equimolar multielements creating pure and stable phases of MPE MAX phases and link the synthesizability of quaternary high‐entropy MAX to an entropy‐driven stabilization. Maximizing configurational entropy to stabilize (near) equimolar mixtures in a manner similar to that in other disordered multicomponent systems (e.g., bulk ceramics and metals) allowed for the successful synthesis of high‐entropy MAX and MXene materials.

## 5 Applications of Non‐Ti MXenes

### 5.1 Sc‐MXene Applications

**Figure 23:**
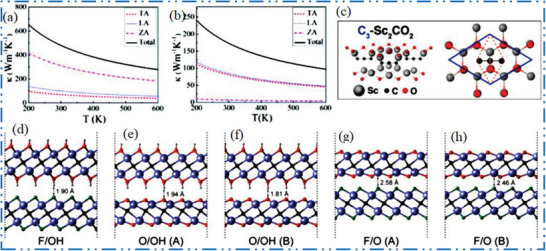
Thermal conductivities of a) Sc_2_CF_2_ and b) Sc_2_C(OH)_2_ along armchair directions, Reproduced with permission.^[^ ^270^ ^]^ Copyright 2016, Royal Society of Chemistry, c) structure and energetic stability of C_3_‐Sc_2_CO_2_, Reproduced with permission.^[^ ^269^ ^]^ Copyright 2021, Wiley, side view of d) F/OH, e) O/OH (A), f) O/OH (B), g) F/O (A), and h) F/O (B) hetero‐systems, Reproduced with permission.^[^ ^223^ ^]^ Copyright 2015, American Chemical Society.

The few experimental experiments that have been examined for Sc‐MXene have allowed systematic computational research to completely anticipate their characteristics. The characteristics of Sc_2_CT_2_ (T = F, OH, O, Cl) MXene NMs were studied using first‐principles calculations.^[^ ^266^, ^267^ ^]^ Sc_2_C(SH)_2_, Sc_2_CCl_2_, and Sc_2_NO_2_ were projected to be semiconductors whereas Sc_2_C(OH)_2_ and Sc_2_CF_2_, for instance, displayed advantageous optical absorption in the vis–light range.^[^ ^268^, ^269^ ^]^ **Figure** **23**a,b shows the thermal characteristics of the semiconducting materials Sc_2_CF_2_ and Sc_2_C(OH)_2_; Sc_2_CF_2_ exhibited high thermal conductivity (472 W m^−1^ K^−1^). Although while Sc_2_C(OH)_2_ has a lower thermal conductivity (173 W m^−1^ K^−1^) and is less anisotropic than silver, it nevertheless outperforms the metal. Inspire by the ferroelectric hexagonal phase (hex‐Sc_2_CO_2_) and antiferroelectric phase (AFE‐Sc_2_CO_2_) of Sc_2_CO_2_, Bae et al.^[^ ^269^ ^]^ developed a novel structural phase for Sc_2_‐CO_2_ MXenes NM with C_3_ unit (*C2/m* space group), C_3_‐Sc_2_CO_2_ (Figure 23c). In contrast to hex‐Sc_2_CO_2_ (trigonal symmetry) and AFE‐Sc_2_CO_2_, the C_3_‐Sc_2C_O_2_ has a rhombic structure with C_3_ structural units (oblique symmetry). The 2D electride insulator a novel reduced energy C_3_‐structure has demonstrated high applicability as anode NMs for LIBs. The SL Sc_2_CO_2_ with asymmetric configuration was shown to be the most stable model in Type I, II, and III heterostructures of functionalized SL of Sc_2_CF_2_, Sc_2_C(OH)_2_, and Sc_2_CO_2_.^[^ ^223^ ^]^ This is because of the many places where the O functional atom may be found. For the most stable geometries shown in Figure 23d–h, the heterojunctions between bilayers of Sc_2_CX_2_/Sc_2_CY_2_ (where X/Y, X, and Y = F, OH, and O) were also assessed. It is possible to better comprehend the characteristics of Sc‐MXenes that are practical to create before their production by utilizing computational approaches to determine their photonics, electronics, and optoelectronics features. Overall, it seems that the Sc‐based MXene family has intriguing semiconducting characteristics that might be useful for optoelectronic devices. Nonetheless, given the cost and raw material restrictions, a detailed examination of their theoretical qualities should be given serious consideration before beginning any practical research. This will lessen the requirement to manufacture Sc‐MXene NMs at a low cost for use in critical applications.

### 5.2 V‐MXene Applications

**Figure 24:**
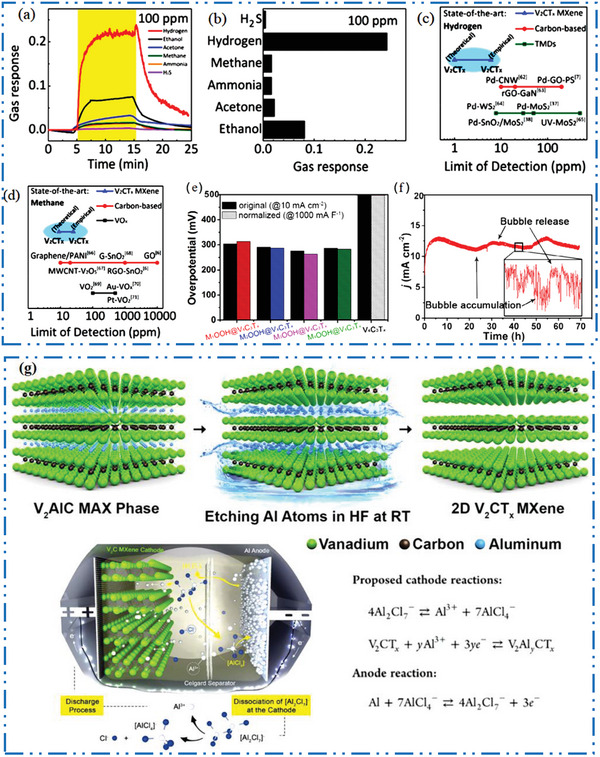
a,b) V_2_CT*_x_* sensor shows resistance variation at 100 ppm of H_2_, C_2_H_6_O, C_3_H_6_O, CH_4_, NH_3_, and H_2_S at RT, and summary of highest response, c,d) Comparison of limit of detection with other C‐ and V‐based materials at RT for H_2_ and methane, Reproduced with permission.^[^ ^272^ ^]^ Copyright 2019, American Chemical Society, e) *η* and *η*Cdl of V_4_C_3_T*_x_* and MOOH@V_4_C_3_T*_x_*, f) CVs of M_3_OOH@V_4_C_3_T*_x_*, Reproduced with permission.^[^ ^273^ ^]^ Copyright 2020, Wiley, g) scheme of V_2_AlC to V_2_CT*_x_* production and ion battery, Reproduced with permission.^[^ ^13^ ^]^ Copyright 2017, American Chemical Society.

Using a thermal image of 2D V_2_C MXene NSs solution at various concentrations, photothermal presentation of vanadium carbide was assessed, and it was shown to be effective for photoacoustic and MRI‐guided photothermal treatment of cancer.^[^ ^149^ ^]^ In terms of energy storage, V_2_CT*_x_*’s theoretically determined specific capacity (125 mAh g^−1^) stood out higher than Nb_2_CT*_x_* (110 mAh g^−1^), industrial graphite, and Ti_2_C.^[^ ^271^ ^]^ There are many opportunities to investigate V‐potential MXene NMs in further energy storage and conversion uses. In conclusion, several experimental investigations for creating the 2D V‐MXene NMs without the use of HF have shown successful results. This may serve as motivation for producing alternative M‐MXenes using less hazardous substances and simple procedures. Furthermore, doping techniques in V‐MXenes have shown positive results in enhancing the substance's general characteristics. The M‐MXene NMs can be used to test this method and introduce advantageous dopants in advanced uses. The remarkable sensitivity of a V_2_CT*_x_* MXene NMs‐based gas sensor toward nonpolar gases was recently described (**Figure** **24**a–d).

When hydrogen gas is present, then V_2_CT*_x_*‐based NMs effectively detects it and their gas response is three times greater than ethanol at 100 ppm at RT. For SNR = 3, it was calculated that the theoretical detection limits for hydrogen and methane were 1.3 and 25 ppm, respectively.^[^ ^272^ ^]^ It is not yet completely understood how inert nonpolar gases are sensed. The gas sensing performance of V_2_CT*_x_* NM is noticeably different from that of Ti_3_C_2_T*_x_* MXene, in which the same group has previously examined.^[^ ^274^ ^]^ Due to their broad range of gas‐selectivity and tunability, the huge family of MXene NMs is suggested a very useful gas sensing platform. The obvious unanswered problems for their gas sensing mechanism are the functions of transition‐metal species and the population of surface functional groups. The unique nine atomic multilayer V_4_C_3_T*_x_* was explored by Du et al. (Figure 24e,f).^[^ ^273^ ^]^ The nanohybrid with the optimum Ni/Fe ratio can achieve a current density of 10 mA cm^−2^ at overpotential (*η*) as low as 275.2 mV, which is superior to other MXene NMs based derivatives and similar to the majority of the state‐of‐the‐art OER catalysts. We may imagine the prospective function of the M_4_X_3_‐based MXene NMs as substrates for a variety of energy conversion and storage materials. It is demonstrated by variable physicochemical characteristics and high structural stability of these nanohybrids. In order to create a intercalation‐type cathode material for Al batteries with outstanding cycle stability and high energy density, Vahidmohammadi et al.^[^ ^13^ ^]^ showed that V_2_CT*_x_* NMs may reversibly intercalate Al^3+^ cations into their structures. Among the cathode materials for Al batteries that have been described so far, V_2_CT*_x_* NMs based electrodes have one of the greatest performances. The method they used to construct the unique ALB with the suggested charge–discharge mechanism at the liquid–solid boundary is schematically illustrated in Figure 24g. At a current density of 100 mA g^−1^, the TBAOH‐FL‐V_2_CT*_x_* cathode produced unusually high specific capacities of more than 300 mAh g^−1^. As a result, our research created a new route for raising ALB performance. As a result, an ALB with an MXene‐based composite cathode (F‐Ti_3_C_2_T*_x_* @Ag) was created for another investigation. After 2000 cycles at a current density of 0.5 A g^−1^, the new ALB's discharge‐specific capacity was around 150 mAh g^−1^.^[^ ^275^ ^]^

**Figure 25:**
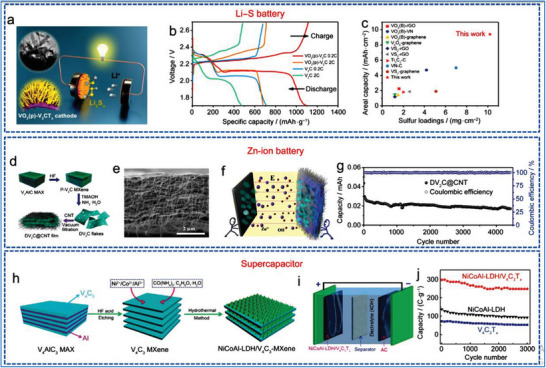
a) Scheme of VO_2_(p)–V_2_CT*_x_* cathode, b,c) Charge–discharge profiles, d) fabrication of delaminated freestanding film, e) SEM of D‐V_2_C@CNT, f) scheme of zinc hydroxide sulfate hydrate nanoflakes, and g,h) cycling performance and columbic efficiency, and scheme of synthetic process composite. i) Scheme shows hybrid SC device, j) cycling performance of electrodes in 1 m KOH. Reproduced with permission.^[^ ^203^ ^]^ Copyright 2020, Springer.

Rechargeable ZIBs, in addition to the typical battery types previously described are a battery technology that has drawn a lot of interest. In 2020, Venkatkarthick et al.^[^ ^276^ ^]^ synthesized a V_2_O*_x_* @V_2_CT*_x_* that could serve as an efficient cathode material for aqueous ZIB. The prepared V_2_O*_x_* @V_2_CT*_x_* electrodes achieved an average optimum rate performance. Wang et al.^[^ ^277^ ^]^ described a 3D‐grid heterostructure made of paramontroseite‐VO_2_ (VO_2_(p)) nanorods based cluster and 2D V_2_CT*_x_* MXene NM (**Figure** **25**a). In contrast to other V‐based cathode materials, the galvanostatic charge–discharge curves of cathode indicate strong discharge capacity performance (Figure 25b), and cathode has greater sulfur areal loading and bigger areal capacity of 9.3 mAh cm^−2^ (Figure 25c).

Wang et al.^[^ ^278^ ^]^ created a Zn‐ion SCs electrode using a CNT network that was evenly mixed with 2D V_2_CT*_x_* MXene NMs (Figure 25d–f). The long‐term cycling performance demonstrates a cycling life of over 4000 cycles at 0.5 A g^−1^ current density with a Columbic efficiency of ≈100% (Figure 25g). Researchers have showed that the structural stability of V_4_C_3_T*_x_* is enhanced over V_2_CT*_x_* due to an increased number of layers, making it a suitable material for energy storage.^[^ ^38^ ^]^ Wang et al.^[^ ^279^ ^]^ created a new supercapacitor heterostructure using Ni–Co–Al layered double hydroxide (NiCoAl‐LDH) and 2D V_4_C_3_T*_x_* MXene NM (Figure 25h,i). Their cycling stability of in 1 mol L^−1^ KOH at a current density of 20 A g^−1^ seems intact after 3000 cycles with a specific capacity of 248 C g^−1^ (Figure 25j). The 2D V‐MXene NMs also provide up new possibilities for white laser production and HER. It is also worth noting that V‐MXene improves capacity when compared to the 2D V‐MXene NMs. For example, Wang et al.^[^ ^280^ ^]^ synthesized 2D (V*_y_*, Ti_1‐_ *_y_*)_2_CT*_x_* NMs and investigated their applicability in LIBs. The addition of the heteroatom Ti to 2D (V*_y_*, Ti_1‐_ *_y_*)_2_CT*_x_* NMs increases their reversible capacity. The 2D V‐MXene NMs, like Mo‐containing 2D MXene NMs, have restricted applicability. Han et al.^[^ ^69^ ^]^ recently revealed that the electrical conductivity of Nb*_y_* V_2‐_ *_y_* CT*_x_* film rises with increasing V content, resulting in improved EMI shielding efficacy.

### 5.3 Cr‐MXene Applications

To predict catalytic performance and structural stability of Cr_2_CO_2_ MXenes with TM alteration and carbon vacancy engineering, DFT simulations were assessed.^[^ ^168^ ^]^ As weaker (25%) link between H atom and surface of Cr_2_CO_2_, the presence of carbon vacancies enhances HER activity. The bonding between the H atom and H_2_ molecules on potential sites in Cr_2_C was assessed by Yadav et al.^[^ ^153^ ^]^ The sum of chemisorption (1.2 wt%), weak electrostatic interactions (3.2 wt%), and Kubas interaction (3.2 wt%) yields the reversible H_2_ storage capacity of Cr_2_C at 7.6 wt%. The issue still resides in making surface terminated Cr‐MXenes, despite theoretical studies showing encouraging results. In conclusion, creating Cr‐based MAX phases necessitates laborious techniques, departing from the tried‐and‐true MAX formulas. On the plus side, Cr‐MAX phases are prepared using safer and less hazardous HF‐free procedures instead of traditional ones. About 2D Cr‐MXene NMs, its applicability for catalytic and magnetic activities is constrained. A huge potential may be concentrated on its synthesis and examination in many applications because of its cheaper cost and natural abundance.

### 5.4 Y‐MXene Applications

Two different etching methods such as HF and DMSO delamination (sample A) and combined HCl as well as LiF solution (sample B), for YAl_3_C_3_,^[^ ^172^ ^]^ produced specific capacitances of 18 and 6 F g^−1^, correspondingly. Also, a positive slope from Mott–Schottky plot along with the negative flat‐band potential (2.1 V vs Ag/AgNO_3_) show that Y_2_CF_2_ exhibits n‐type semiconductor behavior (1.9 eV B.G).^[^ ^173^ ^]^ Nevertheless, Y_2_CF_2_ was unstable and underwent oxidative degradation to generate fluorocarbon and carbonate species when used as a photocatalyst. Structural, electrical, optical, and thermal characteristics of Y‐MXene^[^ ^281^ ^]^ as well as their prospective uses have been researched using first‐principles simulations. For instance, the Y_2_C monolayer has a high theoretical specific capacity of 564 mAh g^−1^, a low diffusion barrier of 0.01 eV, and a substantial Na adsorption energy of ≈0.32 eV.^[^ ^282^ ^]^ Therefore, Y‐MXene has demonstrated good thermoelectric, electrochemical, and semiconductive capabilities when evaluated theoretically. In conclusion, it is still crucial to conduct experimental assessments of the stability, surface functionalization, and characteristics of Y‐based MXene NMs. Among the M‐MXene NMs, yttrium continues to be the least researched metal. First‐principles research has revealed the potential characteristics of Y‐MXene. Yet, rigorous experimental evaluation and additional testing of these features in a variety of applications are still necessary before they can be compared to those of other M‐MXenes.

### 5.5 Zr‐MXene Applications

Metallic qualities were present in Zr_3_C_2_F_2_, Zr_3_C_2_O_2_, and Zr_3_C_2_(OH)_2_, with Zr_3_C_2_O_2_ containing highest mechanical strength (c11 value of 392.9 GPa).^[^ ^177^ ^]^ In fact, it can influence the 2D MXene NMs general characteristics, and can result in optimal applications. Surface‐terminated Zr‐MXene was successfully tested in water‐splitting,^[^ ^283^ ^]^ electrocatalysis,^[^ ^284^ ^]^ and batteries^[^ ^285^ ^]^ using theoretical calculations. High‐theoretical Na atom capacities computed at 474 and 326 mAh g^−1^, correspondingly, Zr_2_CO_2_ and Zr_3_C_2_O_2_ function as metals and semiconductors.^[^ ^285^ ^]^ In the meanwhile, Zr_2_C (310 mAh g^−1^), Zr_2_CO_2_ (266 mAh g^−1^), Zr_2_CS_2_ (259 mAh g^−1^), and Zr_2_CF_2_ (58 mAh g^−1^) have been predicted to have specific Li capacities.^[^ ^286^ ^]^ This type of surface functionalization can offer a tactical method for forcing maximal functionalities onto any MXene. As a result, in situ chalcogenation (Se, S, and Te) and other approaches, such as coprecursor addition, can be researched in MXene NMs to further increase the conversion of highly desirable T*_x_* functional groups.

### 5.6 Hf‐MXene Applications

Based on first‐principles DFT, theoretically studied T*_x_* groups on Hf_3_C_2_T_2_ (T = F, O, OH) projected that Hf_3_C_2_O_2_ would have a high mechanical strength.^[^ ^55^ ^]^ Meanwhile, the OAS substitutions in Hf‐MXene have adjustable characteristics that lead to a range of responses in achieving B.Gs that may be relevant for a number of applications.^[^ ^184^ ^]^ A perfect hydrogen storage solution, Li/Hf_2_CF_2_, was made possible by Li‐decoration on F‐based T*_x_* Hf‐MXene NMs.^[^ ^182^ ^]^ The Hf‐MXene displayed reversible volumetric capacities of 1567 and 504 mAh cm^−3^ used in LIBs and NIBs, correspondingly.^[^ ^55^ ^]^ As a result, assessing Hf‐MXenes NM in applications needing superior electrochemical and electrocatalytic capabilities is a wonderful option.

### 5.7 Nb‐MXene Applications

In comparison to Ti_2_C and Ti_3_C_2_, the 2D Nb_2_C‐based MXene NMs demonstrated the best K‐ion capacitor performance with strong pseudocapacitive subject performances, quick kinetics, and long‐lasting cycle stability.^[^ ^287^ ^]^ Achieving a high energy density of 146.7 Wh kg^−1^ (50.3 mAh g^−1^ for 2.0 V),^[^ ^288^ ^]^ Nb_2_CT*_x_* MXene beat its reported aqueous MXene equivalents. While F, O, and OH are the most often included termination groups because of the interaction between H_2_O and F^−1^ and with bare Nb_2_C,^[^ ^192^ ^]^ Cl, NH, Se, S, Br, and Te are also studied to see how they affect the MXene NMs' property that causes some of their devices uses.^[^ ^289^ ^]^ Conduction band minimum of perovskite layer is greater or lower than the work function (WF) of O, OH, and F terminated Nb_2_CT*_x_*, which may be changed by varying the number of surface T*_x_* of MXene NM.^[^ ^290^ ^]^ Nb_2_CT*_x_* MXene NSs have successfully incorporated ─NH_2_ groups on their surface, as shown by the core‐level spectra N1s and F1s.^[^ ^291^ ^]^ With this method, the WF may be adjusted to suit the desired minimum conduction band for perovskite. The Nb‐based MXene NMs were tested thus far for a variety of uses, including photonics and optoelectronic devices,^[^ ^292^ ^]^ metal ions sensing and fluorescence imaging,^[^ ^293^ ^]^ medical implants,^[^ ^294^ ^]^ radiotherapy,^[^ ^295^ ^]^ batteries,^[^ ^296^ ^]^ capacitors,^[^ ^193^ ^]^ catalysts,^[^ ^297^ ^]^ and hydrogen storage.^[^ ^298^ ^]^ The Nb‐MXene NMs' flexibility shows a promising qualities and applications that may compete with Ti‐MXenes and other non‐Ti MXene NMs.

**Figure 26:**
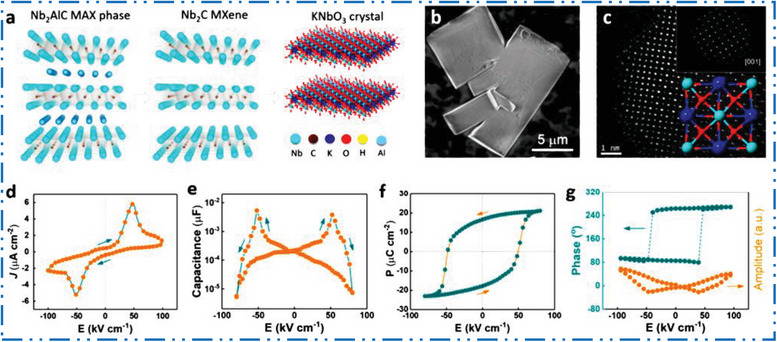
a) Crystal structure conversion of Nb_2_AlC to Nb_2_CT*_x_* and M‐KNbO_3_ by hydrothermal conversion, b,c) SEM and TEM of M‐KNbO_3_, and d–g) ferroelectric results of M‐KNbO_3_ crystals at RT. Reproduced with permission.^[^ ^192^ ^]^ Copyright 2019, Wiley.

Using Nb_2_CT*_x_* NM as a precursor material in a hydrothermal technique, KNbO_3_ ferroelectric crystals with a high aspect ratio and homogeneous shape have been created (**Figure** **26**).^[^ ^192^ ^]^ The Nb_2_CT*_x_* MXene was heated to 190 °C for 48 h in a KOH solution with sodium dodecyl sulfate (SDS) surfactant to undergo simultaneous oxidation and alkalization. Strong ferroelectric characteristics, such as a saturated polarization, a residual polarization, and a coercive field of 21 µC cm^2^, 17 µC cm^2^, and 50 kV cm^−1^, were present in the MXene‐derived KNbO_3_ crystals at room temperature. When Nb_2_CT*_x_* MXene is substituted for commercial NbC powder under the identical conditions, cube‐like KNbO_3_ powders with a nonuniform size distribution are produced. The final products' form is erratic in the absence of SDS surfactant. The 2D layered structure of Nb_2_CT*_x_* and the shape‐modulation impact of SDS served as the foundation for the effective synthesis of high‐aspect‐ratio KNbO_3_ ferroelectric crystal. In essence, the 2D character of MXene is inherited by KNbO_3_, leading to a highly rough crystal shape.

**Figure 27:**
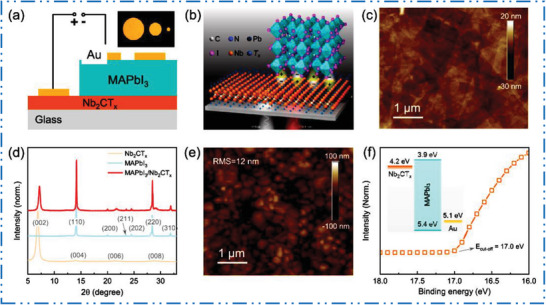
a–f) Scheme of heterostructure built of MAPbI_3_ perovskite and 2D Nb_2_CT*_x_* MXene NM and corresponding characterization (AFM and XRD) and photodiodes applications. Reproduced with permission.^[^ ^34^ ^]^ Copyright 2021, American Association for the Advancement of Science (AAAS).

The capacity of 2D MXene NMs to absorb light effectively is considerably enhanced by surface plasmons vibrating at their 2D surfaces. The 2D MXene NMs have demonstrated excellent plasmonic absorptions in the vis and IR regimes thus far. However, their potential utility in IR optoelectronic uses, such as photodiodes, has received only cursory attention. Furthermore, due to their inherent strong dark current, their comparatively poor resistivity has hampered their usage as photosensing materials. Therefore, Zhixiong Liu et al.^[^ ^299^ ^]^ studied heterostructures built of MAPbI_3_ perovskite and 2D Nb_2_CT*_x_* MXene NM with a matching band structure are created and used to power self‐powered vis–NIR photodiodes (**Figure** **27**). Using MAPbI_3_ has allowed the MAPbI_3_/Nb_2_CT*_x_* photodiode to operate in the vis‐regime while suppressing the comparatively substantial dark current of the NIR‐absorbing Nb_2_CT*_x_*. As a result, the manufactured photodiode reacted linearly to white light illumination with a responsivity of 0.25 A W^−1^ and a photoresponse time of <4.5 µs. Furthermore, when irradiated with an NIR laser (1064 nm), our photodiode has a greater on/off ratio (≈10^3^) and faster reaction times (30 ms) than planar Nb_2_CT*_x_* NMs only detectors (2 and 20 s, respectively). The space–charge–limited current and capacitance measurements show that the coordinate bonding between the surface groups of the 2D MXene NM and the undercoordinated Pb^2+^ ions of the MAPbI_3_ at the passivated MAPbI_3_/Nb_2_CT*_x_* interface is responsible for such an efficient and enhanced charge transfer.

**Figure 28:**
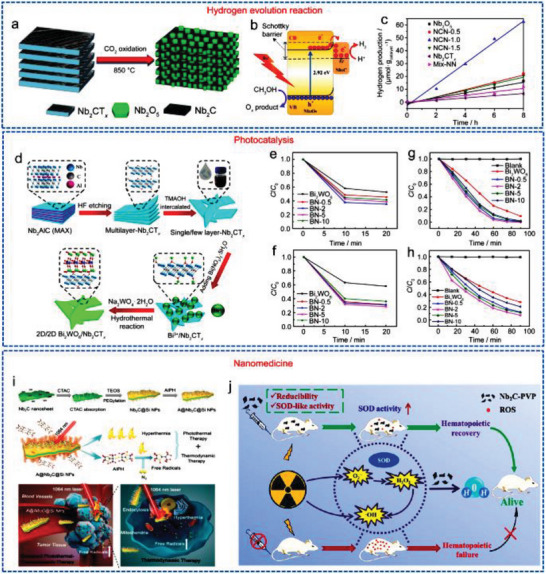
a,b) Scheme shows synthesis of Nb_2_O_5_/C/Nb_2_CT*_x_* composite, and its photocatalytic method, and c) H_2_ creation activity, d) scheme shows NSs synthesis, e,f) absorption of RhB and MB in existence of Bi_2_WO_6_, and g,h) photodegradation of RhB and MB in the presence of Bi_2_WO_6_. i) Scheme shows synthesis of AIPH@Nb_2_CT*_x_* @mesoporous (m) SiO_2_ NPs, and j) hematopoietic recovery after radiation by free radical scavenging. Reproduced with permission.^[^ ^203^ ^]^ Copyright 2020, Springer.

Su et al.^[^ ^300^ ^]^ developed a Nb_2_O_5_/C/Nb_2_CT*_x_* hybrid material as a photocatalyst for HER (**Figure** **28**a,b), which exhibits good hydrogen generation activity of 7.81 mmol h^−1^ g^−1^ catalyst (Figure 28c). Cui et al.^[^ ^301^ ^]^ produced another example in which Bi_2_WO_6_/Nb_2_CT*_x_* (Figure 28d) was used as a photocatalyst for the breakdown of organic contaminants. Rhodamine‐B (RhB) and methylene blue (MB) absorbed extremely well in vis–light (Figure 28e–h). Nb_2_CT*_x_* also has biomedical uses because to its enzyme/H_2_O_2_‐responsive biodegradability and biocompatibility. Xiang et al.,^[^ ^302^ ^]^ for example, developed mesoporous silicon on the surface of Nb_2_CT*_x_*, where mesopores function as initiator carriers and 2D MXene NM induces photothermal effects at the near‐infrared‐II biowindow (NIR‐II). The 2D Nb_2_CT*_x_* confers photothermal stability and strong NIR‐II absorption in the tissue transparency biowindow (Figure 28i). As a result, initiators are rapidly released and degraded, producing oxygen‐independent free radicals. Similarly, Ren et al.^[^ ^295^ ^]^ created the Nb_2_CT*_x_*‐PVP core/shell structure. After radiotherapy, this hybrid substance was certified as hematopoietic recovery medication (Figure 28j).

It is worth noting that the limited uses of 2D Nb‐MXene NMs hint that they may encounter the same issues as other hybrid MXene systems. Nb‐MXene NMs outperform their pure equivalents in EMI shielding applications. In EMI shielding, for example, NbyV_2‐_ *_y_* CT*_x_* (15–36 dB depending on the amount of V) outperforms Nb_2_CT*_x_* (10–15 dB) at 8.2–12.4 GHz. However, for energy storage, the addition of heterocomponents may result in inefficiency. After 20 cycles, the specific capacities of Nb‐containing MXenes (e.g., (Nb_0.8_, Ti_0.2_)_4_C_3_T*_x_* (158 mAh g^−1^) and (Nb_0.8_, Zr_0.2_)_4_C_3_T*_x_* (132 mAh g^−1^)) are somewhat lower than Nb_4_C_3_T*_x_* (189 mAh g^−1^).^[^ ^303^ ^]^ After 100 cycles at a current density of 100 mA g^−1^, Nb_3.5_Ta_0.5_C_3_T*_x_* and Nb_3.9_W_0.1_C_3_T*_x_* had lower specific capacities of 38 and 35 mAh g^−1^, respectively, than Nb_4_C_3_T*_x_* (52 mAh g^−1^).^[^ ^304^ ^]^ More efforts should be made to explain the structures and characteristics of 2D Nb‐MXene NMs, as well as to discover their benefits and game‐changing applications.

### 5.8 Mo‐MXene Applications

**Figure 29:**
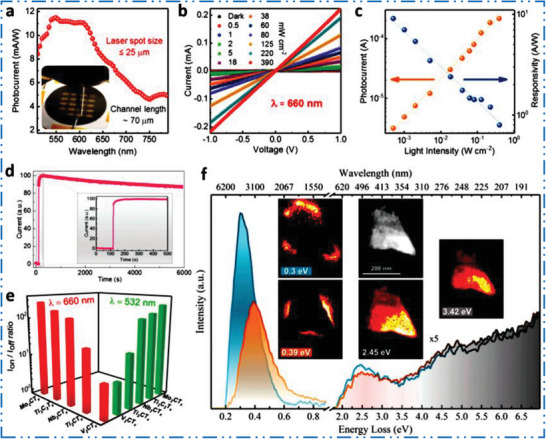
Photoresponse based on Mo_2_CT*_x_*, a) photocurrent at 0.7 V bias voltage, b) linear *I*–*V* graph under different light intensities (660 nm), c) photocurrent and responsivity as a function of light intensity, d) photostability under continuous illumination, e) *I*_on_/*I*_off_ ratio under excitation wavelengths of 660 and 532 nm, and f) zero‐loss peak subtracted EELS spectra of a Mo_2_CT*_x_* with a truncated triangular shape. Reproduced with permission.^[^ ^312^ ^]^ Copyright 2019, Wiley.

Mo‐MXene has shown to be more effective as catalyst for hydrogen evolution than conventional Ti‐MXene.^[^ ^305^ ^]^ As compared to b‐Mo_2_C and Cu–ZnO–Al_2_O_3_ standard, T*_x_*‐free, Mo‐terminated 2D‐Mo_2_C demonstrated astounding catalytic activity and stability.^[^ ^306^ ^]^ The lack of surface terminal groups showed that 2D‐Mo_2_C was more active and selective in CO_2_ hydrogenation. Several researches have demonstrated tremendous relevance in engineering a development of Mo‐based MXene by tweaking its features. One method for dealing with host polymorphism in Mo_2_C MXene crystals is phase engineering.^[^ ^307^ ^]^ Diffusion barrier in elemental precursors of Mo and C may be adjusted by varying the thickness of copper foil used as a catalyst, which also allows for fine control of the development rate of the Mo_2_C crystal. A preferential HER electrocatalytic reactivity for Bernal (AB) stacking as opposed to traditional AA‐stacked crystals may be achieved in Mo‐MXene by controlling the stacking type.^[^ ^307^ ^]^ As in the case of the Fe‐^[^ ^308^ ^]^ and Co‐substitution in Mo_2_CT*_x_*,^[^ ^309^ ^]^ metal substitution may be housed inside the configuration of MXene NMs, which can also boost a electrocatalytic activity. The Mo‐based MXene has also been used in growth of new NMs, such as CoP/Mo_2_CT*_x_* catalysts for energy conversion and water splitting,^[^ ^310^ ^]^ Mo‐based MXene NMs in doped CNTs as HER electrocatalysts,^[^ ^310^ ^]^ and Mo_2_CT*_x_* for humidity monitoring^[^ ^311^ ^]^ (**Table** **1**). Among the MXenes, Mo_2_CT*_x_* performed the best in the visible spectral range responding at 9 A W^−1^ and detecting at 5 × 10^11^ Jones, respectively. It was discovered that the surface plasmon‐assisted hot electrons in the Mo_2_CT*_x_* film play a significant role in the photocurrent. Transverse surface plasmon energy of Mo_2_CT*_x_* coincides with wavelength region of 400–650 nm, where a significant photocurrent was discovered. Under incident light of 660 nm and a bias of 1 V, the highest *I*_on_/*I*_off_ ratio of 200 was discovered. The Mo_2_CT*_x_* thin‐film photodetector has outstanding stability under continuous illumination and in addition repetitive mechanical stress to good photodetection capability (**Figure** **29**).

**Table 1:** Non‐Ti MXene NMs, with production, key findings, and applications.

M‐MXene	Preparation	Key findings	Applications	Refs.
Sc_2_CO*_x_*	MS‐based deposition	Direct production, and no use of etchant and MAX precursors Sensitive to O_2_	Light absorption spectrum and photoluminescence	[139]
ScC*_x_* OH	Etching (TMAOH) of ScAl_3_C_3_	HF‐free technique Traces of Al(OH) 4‐surface groups and intercalated TMA^+^ ions detected	Direct B.G semiconductor	[138]
V_2_CT*_x_*	HF‐etching of V_2_AlC MAX	High contents of O and OH terminations than F, causes high gas adsorption ability	Gas sensors	[272]
V_2_CT*_x_*	HF‐based etching of V_2_AlC	Contains MAX precursor residue, functionalized layers, and stacked layers limiting the capacity	Positive electrode for NIBs	[319]
V_2_O*_x_* @V_2_CT*_x_*	(NaF + HCl)‐based etching of V_2_AlC MAX precursor	Formation of V_2_O*_x_* at high heat treatment based etching technique and electrochemical active process Impurity/formation of by‐product Na_5_Al_l3_F_14_	ZIBs	[276]
V_2_N	HF‐based etching of V_2_AlN MAX precursor	V_2_N can generate abundant reactive O_2_ species that can eradicate bacteria (in vitro) and promote the healing of subcutaneous abscesses with negligible toxicity (in vivo)	Photothermal‐enhanced anti‐infective therapy	[320]
V_2_CT*_x_*	HF‐based etching of V_2_AlC	In situ generation of VO*_x_* through electrochemical oxidation of V during the initial charging, transforming V valence from V^2+^/V^3+^ to V^4+^/V^5+^, all while maintaining inner V–C–V layer residual V_2_AlC are still formed	ZIBs	[321]
a‐VO*_x_*/V_2_C	HF‐based etching of V_2_AlC	Anodic oxidation of MLV_2_CT*_x_* reversible V–O vibration and valence evolution (V^4+^/V^5+^) in disordered framework	Cathode for NIBs	[322]
Cr_2_CT*_x_*	Chemical etching (FeCl_3_ and tartaric acid) of Cr_2_AlC	Prevented the formation of insoluble alumina existence of Cr_7_C_3_ as phase impurity	Sensor for hydrazine detection	[158]
Cr_2_C	Etching (LiF/NaF + HCl) of Cr_2_AlC	Formation of residual salts Incomplete removal of Al	Evaluation of magnetic properties	[323]
Y_2_CF_2_	Solid‐state reaction of Y_2_C, YC, and YF_3_	Direct synthesis, and presence of YF_3_ and YOF impurity phases not very stable during photoreaction undergoing oxidative degradation (formation of fluorocarbon and carbonate species)	Vis–light induced photocatalytic activity	[173]
Y_2_CF_2_	Solid state reaction of YF_3_,Y‐metal, and graphite	Direct synthesis, and controlled F‐termination high purity of Y_2_CF_2_ easy oxidation because of air exposure	Evaluation of electronic structure and oxidations sensitivity	[171]
YC*_x_* Td etching (LiF + HCl) of YAl_3_C_3_	HF‐based etching of YAl_3_C_3_, Y_2_O_3_ impurity Nonuniform sheets	Y_2_O_3_ impurity	LIBs as well as SCs electrode	[172]
Zr_3_C_2_T*_z_*	HF‐based etching of Zr_3_Al_3_C_5_	Traces of rock‐salt‐like cubic ZrC impurities Zr_3_C_2_ shows superior thermal stability than Ti‐based MXene	Evaluation of structural, mechanical, and electronic properties	[177]
ZrC	MS‐deposition	Direct production, and controlled film quality	Optoelectronic material for ultrafast mode‐locked fiber lasers	[324]
Nb_2_C	HF‐based etching of Nb_2_AlC	Al layer by selective etching	Evaluation of ultrafast relaxation dynamics and nonlinear response	[292]
Nb_2_C	HF‐based etching of Nb_2_AlC	Removal of Al layer by selective etching	Electrodes for LIBs	[271]
Nb_4_C_3_T*_x_*	HF‐based etching of Nb_4_AlC	High breakdown current density of 1.1 × 10^8^ A cm^−2^	Evaluation of electronic properties and high breakdown current density response	[325]
Nb_2_CT*_x_*	HF‐based etching of Nb_2_AlC	Work functions are modulated by replacing the surface terminated ─F groups with ─NH_2_ group	Electron transport layer and perovskite additive for perovskite solar cells	[291]
Nb_2_CT*_x_*	HF‐based etching of Nb_2_AlC	Occurrence of unreacted Nb_2_AlC and residual Al‐containing secondary phases	Evaluation of electronic and mechanical properties, and surface chemistry	[326]
Mo_2_CT*_x_*	HF‐based etching of Mo_2_Ga_2_C	Relative stability against oxidation compared to Ti_3_C_2_T*_x_*, Nb_2_CT*_x_*, T_2_CT*_x_*, and V_2_CT*_x_* possible oxidized forms of Mo–O moieties in product	Plasmonic photodetection	[312]
Mo_2_C	UV‐induced etching (H_3_PO_4_) of Mo_2_Ga_2_C	HF‐free production of high‐quality F‐free MXene NMs	LIBs and NIBs	[327]
Mo_2_CT*_x_*	HF‐based etching of Mo_2_Ga_2_C	Optimization of Ga removal (HF at 140 °C after 96 h)	Catalytic activity in the water‐gas shift reaction	[328]
Mo_2_CT*_x_*	HF‐based etching of Mo_2_Ga_2_C	Stability in acid Mo_2_CT*_x_* was found to shows high HER activity than Ti_2_CT*_x_*	HER	[305]
Mo_2_N	Ammoniation of Mo_2_CT*_x_*	Retained MXene structure upon transformation conductivity values of Mo_2_N are three magnitudes larger than Mo_2_CT*_x_*	Evaluation of thermoelectric properties	[329]
Mo_2_C	Epitaxial synthesis through chemical conversion of MoS_2_	Excellent sheet resistance (123.6 Ω sq^−1^) and carrier concentration (5.84 × 10^13^ cm^−2^)	Evaluation of electronic properties	[202]
Hf_3_C_2_T*_z_*	HF‐based etching of Hf_3_[Al(Si)]_4_C_6_	Trace of unreacted Hf_3_[Al(Si)]_4_C_6_, and small amount of cubic HfC impurities more than 65% conversion from MAX to MXene NMs	LIBs and NIBs	[174]
Ta_4_C_3_	HF‐based etching of Ta_4_AlC_3_	Surface modification improved biocompatibility and physiological stability with no noticeable toxicity	Dual‐mode photoacoustic/CT‐scan and in vivo photothermal ablation of tumors for biomedical uses	[330]
Ta_4_C_3_	HF‐based etching of Ta_4_AlC_3_	Third‐order nonlinear optical characteristics	Mode‐locked fiber lasers	[316]
Ta_4_C_3_T*_x_*	HCl‐based etching of Ta_4_AlC_3_, then with KOH solution	F‐free etching method 84% removal of elemental Al promoted higher degree of OH and O groups functionalization	Biocompatible material for SCs electrodes	[317]
W_1.33_CT*_x_*	Etching (HF) of (W_2/3_Y_1/3_)_2_AlC or (W_2/3_Sc_1/3_)_2_AlC	Trace of MAX phase	Electrode for SCs	[216]
W_1.33_C	Etching (HCL + LiF) of (W_2/3_R_1/3_)_2_AlC (R = Gd, Tb, Dy, Ho, Er, Tm, and Lu)	Antiferromagnetic paramagnetic for *R* = Lu‐derived W‐MXene complicated magnetic moment alignments for *R* = Tb, Dy, and Ho derived W‐MXene	Evaluation of electrochemical properties	[217]
W_1.33_CT*_x_*	Etching (HF) of (W_2/3_Sc_1/3_)_2_AlC Etching (LiF + HCl) of (W_2/3_Sc_1/3_)_2_AlC Etching (HF) of (W_2/3_Y_1/3_)_2_AlC	Ordered metal divacancies Pure phase Ordered metal divacancies Pure phase Ordered metal divacancies Impurity phases of original MAX phase, YAl_3_C_3_, W, and Y_2_O_3_	HER	[97]
W_1.33_C	Etching (LiF + HCl) of (W_2/3_Y_1/3_)_2_AlC	Ordered divacancies and desirable biocompatibility/biodegradability	Tumor retention and theranostic functionalities for biomedical uses	[331]

**Figure 30:**
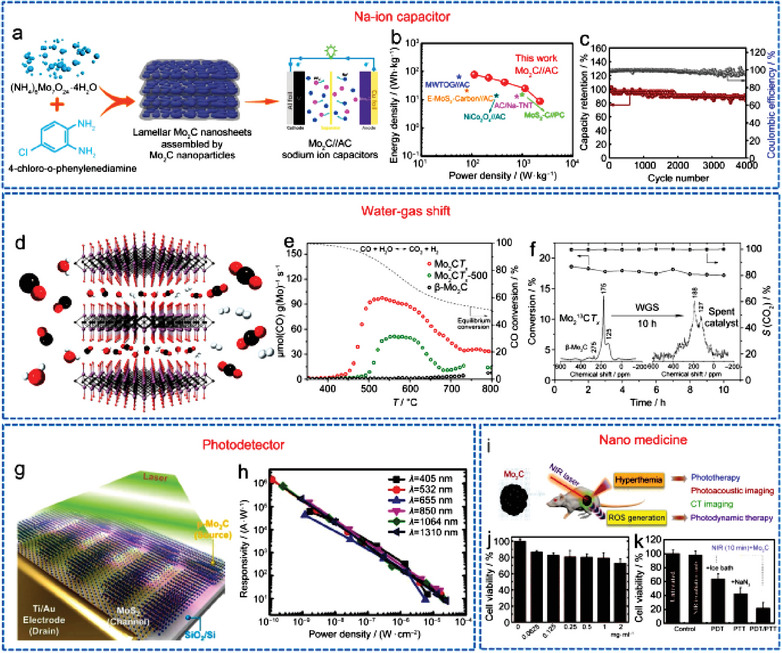
a) Scheme of Mo_2_CT*_x_* NSs synthesis and with Mo_2_CT*_x_* NPs, b) Ragone‐plot, c) cycling stability of electrode at 1A g^−1^, d) scheme of water‐gas shift (WGS), e) WGS catalytic activity test, f) WGS stability test, g) scheme figure of photodetector device, h) responsivity of MoS_2_/multiple periods (mp)‐Mo_2_C hybrid device, i) scheme shows Mo_2_C‐mediated cancer theranostic, j) cell viability, and k) cell viability after PTT, PDT, and synergistic treatment of PDT/PTT. Reproduced with permission.^[^ ^203^ ^]^ Copyright 2020, Springer.

By using EELS mapping, the precise surface plasmon energies and distribution of Mo_2_CT*_x_* were investigated and seen. At 0.30, 0.39, 2.45, and 3.42 eV, four unique peaks were found, and they are categorized as longitudinal dipole, longitudinal quadrupole, transversal modes, and interband transition, correspondingly. It should be noted that only MXenes have such multipolar surface plasmon modes been identified in 2D NMs. From the instance of Ti_3_C_2_T*_x_*, it is known that population of surface termination groups, which closely correlates with the free electron density of 2D MXene NMs, may be controlled to further tailor the longitudinal and transversal surface plasmon energies of MXenes.^[^ ^242^ ^]^ The shape of each flake affects longitudinal dipole and quadrupole modes as well. Each monolayer MXene nanoflake keeps its own surface plasmon modes as separate NSs in the ML film due to the weak interlayer interaction of 2D MXene NMs, which enables the effective production of broadband plasmonic photodetection. Li et al.^[^ ^55^ ^]^ created a sodium‐ion capacitor (NIC) with an anode made of assembled Mo_2_C Ns (**Figure** **30**a). The as‐prepared capacitor had a maximum energy density of 76.1 Wh kg^−1^ at a power density of 112 Wh kg^−1^ and outperformed other active carbon‐based capacitors (Figure 30b). Cycling stability at 1 A g^−1^ current density further demonstrates the good capacity retention (Figure 30c). Mo_2_CT*_x_* catalytic activity in the water‐gas shift process was reported by Sokol et al.^[^ ^51^ ^]^ (Figure 30d). The results show that the 2D Mo_2_CT*_x_* had the highest activity of all investigated catalysts, with the maximal activity attained at roughly 520 °C and a peak CO consumption rate of ≈100 µmol(CO) g(Mo)^−1^ s^−1^ (Figure 30e). Furthermore, negligible deactivation was seen after 10 h on stream at roughly 500 °C, as illustrated in Figure 30f. Anasori et al.^[^ ^49^ ^]^ coupled MoS_2_ and Mo_2_C to create a photodetector with excellent sensitivity and broad spectrum (Figure 30g). This Mo_2_C‐based hybrid device has good responsiveness at various lighting power densities (Figure 30h). Hadi et al.^[^ ^48^ ^]^ used 2D Mo_2_CT*_x_* NM as a theranostic nanoagent in the medical field (Figure 30i). In this setup, high cell viability was attained, confirming Mo_2_CT*_x_*’s superior biocompatibility (Figure 30j,k). Established research on Mo‐MXene NMs has taken the possibility of switching the precursor from MAX to MAB precursors. This provides a fantastic chance to examine MBenes, which may have unique features that is not present in MXene NMs. In addition, investigations on molybdenum could be constrained by their low abundance. Thus, it is important to look into metal substitutes on Mo‐based MXene NMs with competitive characteristics.

### 5.9 Ta‐MXene Applications

Ta element (atomic number = 73) is investigated a 2D structure in biological uses^[^ ^313^ ^]^ because Ta provides strong image contrast that might be advantageous for XRD and CT based imaging. Delaminated 2D Ta_4_C_3_ that had been changed with soybean phospholipid (SP) and MnO*_x_*/Ta_4_C_3_ MXene NSs and modified with SP both demonstrated photoacoustic/CT imaging capacity and biofunctionalities that were suitable for biological uses. Ta‐MXene, a 2D structure, may be used as an effective host to create hybrid materials with extended functionalities, for instance, Ta_4_C_3_‐IONPs, which are very effective theranostic agents for breast cancer.^[^ ^314^ ^]^ Outside of biomedical uses, Ta_4_C_3_ can also provide remarkable conductive qualities^[^ ^315^ ^]^ that have been used and investigated in optoelectronics,^[^ ^209^, ^316^ ^]^ electrodes for supercapacitors (SCs),^[^ ^317^, ^318^ ^]^ and catalysts for water splitting.^[^ ^318^ ^]^ Table 1 provides an overview of other Ta‐MXene production and uses.

### 5.10 W‐MXene Applications

For use in NH_3_ gas sensing applications, the surface T*_x_* of W_2_C with O and F are seen and studied.^[^ ^218^ ^]^ Strong physisorption and robust interactions in the substrate and NH_3_ were seen in both systems, giving O/F terminated W‐based MXene NMs excellent sensor capabilities at low NH_3_ concentration within a broad temperature window. In order to comprehend the variety of potentials of W‐based and other MXene NMs that have not yet been identified, such first‐principles research is highly appreciated. Table 1 shows catalogue of the non‐Ti 2D MXene NMs, with production, key findings, and various novel devices uses.

### 5.11 Double Transition Metal MXenes Applications

In spite of the fact that novel MAX and MXenes developed continually over the past few years, and computational approaches were extremely helpful in forecasting their characteristics and performance.^[^ ^332^ ^]^ The chemical formulas M_2_MC_2_T*_x_* and M_2_′ M_2_″ C_3_T*_x_* have been shown to have the most stable terminations^[^ ^333^ ^]^ (M′ and M″ are two distinct metals; M′ = Cr, V, Ti, or Nb; M″ = Ta, Nb, Ti, or V; and T = O and/or OH). Mo_2_TiC_2_ and Mo_2_Ti_2_C_3_ were anticipated to have the lowest formation energies, and they have previously been created experimentally.^[^ ^334^ ^]^ Ti_2_ZrC_2_O_2_, TiZrCO_2_, and TiZr_2_C_2_O_2_ were projected to have theoretical capacities of 586, 441, and 374 mAh g^−1^, correspondingly. These materials were shown to outperform their monotitanium‐ and mono‐Zr carbides.^[^ ^335^ ^]^ Theoretically, SL Ti_2_MnC_2_T*_x_*, Hf_2_‐MnC_2_T*_x_*, and Hf_2_VC_2_T*_x_* (T = F, OH, O, etc.) are anticipated to have metallic and semiconducting characteristics.^[^ ^260^ ^]^ Understanding these characteristics it will eventually enable one to comprehend how well they operate in various contexts, including HER,^[^ ^333^ ^]^ Al‐,^[^ ^336^ ^]^ and Na‐ion batteries.^[^ ^337^ ^]^ The experimentally formed double TM MXene NMs and their uses are listed in **Table** **2**. Mo–O motifs were used to anchor the ordered double transition‐metal Mo_2_TiC_2_T*_x_* MXene, which was then transformed into Mo─S bonds using the in situ sulfidation technique.^[^ ^338^ ^]^ For anodes in LIBs, the unique heterostructures of FL MoS_2_ on Mo_2_TiC_2_T*_x_* led to high specific capacities and Columbic efficiencies, high rate capability, and outstanding cycle stability. This in situ conversion method is highly intriguing since it uses the primary metals to produce a secondary material with only a small amount of precursor input. In the same way, serving as an inspiration for subsequent research, metal oxides derived from MOFs,^[^ ^339^ ^]^ multiple metals incorporated in MOF structures^[^ ^340^, ^341^ ^]^ or multiple metal‐based oxides,^[^ ^342^, ^343^, ^344^ ^]^ as well as doping methods^[^ ^345^ ^]^ are all examples of synthesis methods that can use a base material to include additional advantageous compounds. Nevertheless, using MXene NMs as the basis NM still need a better theoretical and experimental comprehension of their characteristics. The inherent magnetism and half‐metallic properties of the 2D MXene NMs offer significant promise for spintronic and magnetic devices, such as attaining complete spin‐filtering in vdWs magnetic tunnel junctions (MTJs). Cui et al.^[^ ^346^ ^]^ investigated theoretically the spin‐dependent transport properties of 2D ScCr_2_C_2_F_2_ NMs‐based vdWs MTJs, where ScCr_2_C_2_F_2_ acts as the spin‐filter tunnel barriers, 1T‐MoS_2_ acts as the electrode, and 2H‐MoS_2_ acts as the tunnel barrier. They discovered that spin‐up electrons in the parallel configuration stage play an important role in transmission behavior. All of built MTJs could maintain significant tunnel magnetoresistance (TMR) ratios greater than 9 × 10^5^%. Especially, the maximum giant TMR ratio of 6.95 × 10^6^% can be found in vdWs MTJ with trilayer 2H‐MoS_2_ as the tunnel barrier. These results indicate that the potential for spintronic applications of vdWs MTJs based on 2D ScCr_2_C_2_F_2_.

**Table 2:** Experimental synthesis of double transitional metal MXenes NM and their applications.

Origin of MAX precursor	Etchant	Double metal MXene	Applications	Refs.
TiVAlC	LiF, HCl	TiVCT*_X_*	Dual‐functional antibacterial capability	[348]
Ti*_x_* Ta_4‐_ *_x_* AlC_3_	HF	Ti*_x_* Ta_4‐_ *_x_* C_3_	LIBs anodes	[349]
TiNbAlC	HF	TiNbCT*_x_*	LIBs anode	[350]
Mo_4_VAlC_4_	HF	Mo_4_VC_4_	Electrical/optical properties Evaluation	[108]
Mo_2_TiAlC_2_	HF	Mo_2_TiC_3_T*_x_*	Evaluation of semiconductor properties	[351]
Mo_2_Ti_2_AlC_3_		Mo_2_Ti_2_C_3_T*_x_*		
Mo_2_TiAlC_2_	HF	Mo_2_TiC_2_T*_x_*	Flexible memristors	[352]

### 5.12 Non‐IIIB to VIB Metal Group Applications

First‐principles calculations revealed that the Lu_2_C(OH)_2_ arrangement had an ultralow work function and showed a straight B.G (1.4 eV). In the meanwhile, 4.6 eV was determined as the work function of F‐based MXene NMs, like, Lu_2_CF_2_. This expected low work function for lanthanide‐MXene NMs has significant potential for use in electrical devices. According to first‐principles simulations study of electronic and magnetic characteristics of Mn_2_CT_2_ MXene NMs (T = H, Cl, OH, O, and F),^[^ ^347^ ^]^ Mn_2_CF_2_ is a half‐metal with a large half‐metallic gap and RT ferromagnetism. Evolution of MXene NMs migrates to a completely new adventure with the bordering of MXene NMs beyond the IIIB to VIB family. Theoretical research on Mn‐MAX precursors and Mn‐based MXene NMs, which are members of the VIIB family, may provide attractive and exciting options for studying well‐known battery NMs like nickel, cobalt, etc. Thus, theoretical analyses are highly required to ascertain the likelihood of producing these novel MXene NMs using experimental means. Table 2 shows mostly studied experimental synthesis of double TMs based 2D MXene NMs and their novel technological advanced applications.

## 6 Stability and Advantages of Non‐Ti MXenes and Their Challenges

The 2D MXene NMs' stability at high‐temperature is determined through their composition and surrounding environments. As a result, many investigations have observed varying high temperature behavior. In the existence of O_2_ and H_2_O, MXene NMs based nanoflakes do not remain stable forever.^[^ ^353^ ^]^ In O_2_ free degassed water or dry air, however, they are relatively stable. In addition, light can hasten the oxidation of the colloidal MXene solution. Thus, it is advised that MXene colloids are stored in the O_2_ free, dark environment. Generally, MXene nanoflakes oxidation begins at the edges, resultant in creation of metal oxide nanosize crystals (e.g., TiO_2_) that decorate the nanoflakes edges, with subsequently spreads throughout the whole surface during nucleation and growth.^[^ ^354^ ^]^ Oxidation resistance of 2D MXene NMs is determined by the production process; higher‐quality single nanoflakes MXene have superior oxidation resistance.^[^ ^355^ ^]^ MXenes' high‐temperature stability science is continually evolving. Nonstoichiometric TMCs phase diagrams may aid in predicting 2D MXene NMs phase stability.^[^ ^247^ ^]^ The XRD or Raman analysis required to prove if any novel phases have developed, despite the characterization results seem appealing. The Zr_3_C_2_T*_x_* has been proven for good thermal stability and preserve its 2D nature at about 1000 °C in vacuum. The superior thermal stability of the fact that Zr_3_C_2_ has a more energetically advantageous structure than bulk ZrC. Zr_3_C_2_T*_x_* may be advantageous for high temperature uses because of its increased stability. So far, attempts to synthesize Cr_2_C from Cr_2_AlC have failed.^[^ ^356^ ^]^ One probable explanation is that chromium carbide is less stable than other carbides due to its lower cohesive energy.^[^ ^357^ ^]^ Cr_2_C may develop in aqueous solutions during etching and swiftly convert to other phases, such as chromium oxides. For further details about stability and solution can read our recently published article.^[^ ^358^ ^]^

Titanium was the focus of MXene NMs research on development since it was first time studied. The outstanding selection of TM candidates within the group‐(III–VI)B family member has demonstrated the viability of replacing Ti in MXene NMs, as addressed above in this paper. In the biomedical area, where safety and toxicity are issues, MXene, rely on Ta, V, and Nb, shown biocompatibility and low‐toxicity, which is much appreciated. The electrical characteristics of Nb‐, Mo‐, Y‐, and V‐MXene were excellent and suitable for electrocatalytic and energy storage devices. Using untapped non‐Ti 2D MXene NMs in particular showed improved characteristics to the conventional Ti‐MXene NMs. While Ti_2_C showed less theoretical capacity than V_2_C and Nb_2_C, Mo_2_C showed significantly more HER activity. The study of non‐Ti MXene in semiconductors (Sc, Y, and Zr), lasers (Nb, Ta, and Zr), and sensors (V, Nb, and Cr) has also shown encouraging results. Ti‐MXene make almost 70%, that has more studied and hence has a strong advantage over other non‐Ti MXenes materials because, Mo, Nb, V, Cr, W, Zr, Hf, Ta, Sc, Y, and others make up the remaining 30%. Non‐Ti MXene studies are primarily restricted to theoretical research, despite the fact that they are attracting a lot of interest and attention. As a result, there is a significant research void for non‐Ti MXene uses. In fact, several non‐Ti MXene NMs have not yet been experimentally investigated in SCs, batteries, or catalysis.

Despite this restriction, there is rising interest in studying non‐Ti MXenes using theoretical and computational approaches. The formation energies of several MXene types^[^ ^33^ ^]^ provided the best indication of whether or not these MXenes could be synthesized experimentally. It was projected that the M_2_N adsorption energies (M = Ti, Zr, Hf, V, Nb, Ta, Cr, Mo, and W) would function as effective CO_2_ conversion catalysts.^[^ ^359^ ^]^ Because no practical investigations have comprehensively enumerated the benefits of non‐Ti or M‐based MXene NMs to yet, several papers demonstrated their theoretical qualities that can be associated to their future uses. The various applications that may be influenced by these theoretical qualities can then be connected. In comparison to other M_2_C‐type MXenes, such as Cr, Nb, Mo, Hf, Ta, and W, Sc_2_C, Ti_2_C, V_2_C, and Zr_2_C MXene NMs are projected to exhibit rapid charge/discharge rates due to their lower Li diffusion energy barriers.^[^ ^360^ ^]^ The Sc_2_CO_2_, Ti_2_CO_2_, Zr_2_CO_2_, Hf_2_‐CO_2_, and Mn_2_CO_2_ turned out to be semiconducting for the catalytic activity of the 2D MXene NMs with M*_n_*_+1_CnO_2_ (*n* = 1, 2; M = Sc, V, Cr, Mn, Zr, Nb, Mo, Hf, Ta, and W) configurations. The disadvantages of M‐MXene over other alternatives are also known, such as Sc_2_C(OH)_2_ and Zr_2_C(OH)_2_′s inability to remove Pb in comparison to other M_2_X(OH)_2_ systems (M = V, Cr, Nb, Mo, Hf, Ta, and X = C or N).^[^ ^361^ ^]^ Another illustration shows that when compared to Ti_2_CO_2_, V_2_CO_4_, Nb_2_CO_2_, and Mo_2_CO_2_, Sc_2_CO_2_, Y_2_CO_2_, and Cr_2_CO_2_ did not attain greatest recommended formation energy to produce freestanding 2D MXenes NSs.^[^ ^362^ ^]^ In practice, this might make it challenging and difficult to experimentally synthesize Sc‐, Y‐, and Cr‐based M_2_CO_2_ NMs.^[^ ^363^ ^]^ As compared to other M_2_N‐type MXene (M = Zr, Hf, V, Nb, Ta, Cr, Mo, and W), Cr_2_N, Mo_2_N, and W_2_N are less appropriate materials for possible candidates for CO_2_ reduction.^[^ ^359^ ^]^ The B.Gs of Sc_2_C(OH)_2_ (0.74 eV) and Mo_2_CF_2_ (0.84 eV) are inadequate compared to M_2_C‐type MXenes like Zr_2_CO_2_, Hf_2_CO_2_, Sc_2_CF_2_, Sc_2_CO_2_^[^ ^283^ ^]^ to give sufficient energy for water splitting. Even if the majority of these texts have looked into computational analysis of M‐type MXene NMs, there is still a strong necessitating supporting theories with experimental data. Unfortunately, 2D MXene NMs cannot be prepared in a way that allows for a direct comparison of their attributes to those of another MXene. Therefore, theoretical approaches offer a good grasp of the characteristics of various M‐MXene to decide whether they are practical in particular applications.

## 7 Summary

MXene is a family of 2D materials composed of TMCs, TMNs, or TM carbonitrides. While Ti‐based 2D MXene NMs have been extensively studied, but there are also many non‐Ti MXenes that have been discovered and investigated, such as vanadium carbide, niobium‐based, molybdenum‐titanium carbide, tungsten carbide, etc., MXenes. Many Ti‐based MXenes rely on the extraction of titanium, which can be costly and environmentally damaging. By contrast, non‐Ti MXenes may be synthesized from more abundant and environmental friendly sources, making them an attractive alternative. These non‐Ti MXene NMs exhibit unique properties, such as high electrical conductivity, mechanical strength, thermal stability, and good electrochemical and thermal properties, making them promising candidates for a variety of applications such as energy storage, catalysis, thermal management, and wear‐resistant coatings. Further research is needed to fully explore the potential of non‐Ti MXenes in various fields. The M_2_C‐type (M = Sc, V, Zr, Nb, Mo, Hf, Ta) and M_2_N‐type (M = Zr, Hf) with F, O, and OH MXene systems have strong electrical conductivity but weak thermoelectricity.^[^ ^231^ ^]^ Regarding to various technological novel applications of non‐Ti MXenes, Sc_2_CF_2_, Zr_2_CF_2_, and Mo_2_CF_2_ are the best acceptable cathode material for asymmetric supercapacitors in aqueous and ionic/organic systems.^[^ ^364^ ^]^ Among 2D vanadium carbide MXenes (V_2_C, V_2_CT*_x_*, etc.) the V_2_CF_2_ is the most recommended anode material for asymmetric supercapacitors.^[^ ^364^ ^]^ Theoretically, V_2_NS_2_ demonstrated a larger Na capacity (99.8 mAh g^−1^) than Ti_2_NS_2_ (84.77 mAh g^−1^).^[^ ^365^ ^]^

The M_2_N‐type (M = Zr, Hf) with F, O, and OH‐MXene NMs, V‐, Nb‐, and Ta‐based 2D MXene NMs exhibit high electrical conductivity but relatively subpar thermoelectricity.^[^ ^231^ ^]^ While Zr‐, and Hf‐MXene NMs with F, O, and OH functionalized, exhibit average thermoelectricity while the finest thermoelectricity exhibited by Mo‐ and semiconducting nonmagnetic Cr‐based MXenes.^[^ ^231^ ^]^ The Ta‐, Nb‐, and V‐based MXenes exhibit strong electrical conductivity but low thermoelectricity.^[^ ^231^ ^]^

This shows that 2D Mo_2_C MXene NMs are preserving with the presence of vacancies have improved structural stability, electrical conductivity, and Li storage capacity.^[^ ^366^ ^]^ Among various M_2_C MXene (M = V, Zr, Hf, Nb, Ta, Mo, W), Mo_2_C and W_2_C are among the most promising MXenes for CO_2_ collection and posterior release procedures.^[^ ^367^ ^]^ The Cr_3_C_2_, Mo_3_C_2_,^[^ ^368^ ^]^ and W_2_CO_2_^[^ ^369^ ^]^ 2D MXene NMs show that they have the most promising CO_2_ to CH_4_ selective conversion capabilities.

As compared to other MXenes Sc_2_CO_2_, especially Zr_2_CO_2_, Hf_2_CO_2_, has better hazardous SO_2_ gas detection performance.^[^ ^370^ ^]^ The Mo_2_CO_2_ and V_2_CO_2_ are less effective in adsorbing NH_3_ and NO than Nb_2_CO_2_.^[^ ^231^, ^371^ ^]^ The HER catalytic activity at high H coverage is shown by V_2_CO_2_, Cr_3_C_2_O_2_^[^ ^372^, ^373^ ^]^ while at low H coverage, it is catalyzed by Zr_3_C_2_O_2_, Nb_2_CO_2_, W_2_CO_2_, and W_3_C_2_O_2_.^[^ ^373^ ^]^ The Nb_2_NO_2_ also shows promising HER performance.^[^ ^231^, ^374^ ^]^

The Cr_2_C(OH)_2_, Cr_2_NF_2_, Cr_2_N(OH)_2_, and Cr_2_NO_2_ are ferromagnetic,^[^ ^373^ ^]^ while Zr_2_C and Ti_2_C exhibit spontaneous magnetism.^[^ ^361^, ^362^ ^]^

Y_2_CF_2_ (0.98 eV) and Y_2_CCl_2_ (0.69 eV) are semiconductors with indirect B.Gs, together with Sc_2_CF_2_ (1.06 eV) and Sc_2_CCl_2_ (1.31 eV).^[^ ^281^ ^]^ The Zr_2_CO_2_ and Hf_2_CO_2_ are potential single photocatalysts with excellent photocatalytic efficiency.^[^ ^283^ ^]^

The 2D niobium‐based MXenes NMs (e.g., Nb_2_C and Nb_4_C_3_T*_x_*) are with high mechanical strength and good thermal stability, making them suitable for applications in structural materials and coatings. Only Nb_2_NF_2_ exhibits dynamic and thermal stability in the unique ground state.^[^ ^375^ ^]^

The 2D tungsten carbide MXene NMs (W_2_C and W_2_CT*_x_*) with high mechanical strength and good thermal stability, making them promising for use in wear‐resistant coatings and cutting tools. Compared to Hf_2_CO_2_, the W_2_CO_2_ had greater mechanical strength (592.7 GPa).^[^ ^224^, ^362^ ^]^

Research on 2D non‐Ti MXene NMs is still in its early stages, but the unique properties of these materials suggest that they have great potential for a variety of novel applications.

## 8 Future Prospects of Non‐Ti MXene Materials: Problems, Outlook, and Solutions

Increasing metal options exterior of their typical family group has created new opportunities for the prospective use of other M metals with advantageous features. Although the theoretical and electrical characteristics of the non‐Ti 2D MXene NMs were discussed in this study and only a little amount of research has been done to determine their practical use. The MXenes facing issues are to be solved about preparation of non‐Ti MXenes stoichiometric MAX phase with great purity. Other solution is nonstoichiometric phase preparation such as MAB. The double transition metal MXene and multiple‐metal incorporated MXene approach can be utilized to obtain tuned properties. The in situ growth of non‐Ti MXenes on other MXenes surface can solve the stability issues. The proposed solutions for non‐Ti MXenes are discussed in following section.

### 8.1 Non‐Ti MAB

Currently, scientists are working to create and incorporate extra X elements into 2D boride systems. Generally, the MAX phase is a frequent precursor of 2D MXene NMs but there is another phase precursor, which is MAB. The MAB produces when X (carbon and/or nitrogen) successfully replaced with B (boron). It is important to assess effective experimental MAX and MAB phase preparations with excellent phase purity. Thus, effective manufacturing techniques should be researched in order to remove the A‐layer successfully exclusively producing side results. Since the discovery of MAB phase will require entirely novel study to explore the novel MAB phase combinations that will yield new MBene phases, such as MXene NMs from MAX precursors.

### 8.2 Double Transition Metal MXene and Multiple‐Metal Incorporated MXene

Recent research using computing techniques and first‐principles investigations has led to the discovery of double TM MXene NMs.^[^ ^33^ ^]^ The formation of solid solutions on the M and X sites allows for the synthesis of a sizable number of nonstoichiometric MXenes with precisely controlled characteristics and mixed transition metals or carbon nitrides.

Till now only few experimental studies have demonstrated how to make this double TM MXene. The effective conversion of novel ternary and quaternary MAX phases into equivalent MXenes with excellent purity deserves special emphasis, even though several research have examined these phases.^[^ ^376^ ^]^ Thus, appropriate double‐metal‐MAX phases and etching methods need to be carefully studied. The Ti was the only metal used in the M layer's initial design of MXene. It took the discovery of double TM MXene to introduce a secondary metal by doping in their MAX precursor.^[^ ^309^ ^]^ In light of the evaluation of trimetallic oxides, it may not be difficult to forecast trimetallic TM 2D MXene NMs.

### 8.3 MXene‐Derived NMs

According to intriguing research on in situ sulfurization of Mo‐based 2D MXene NMs, like 2D MoS_2_ heterostructures successfully developed on MXene NM.^[^ ^377^ ^]^ Strong heterojunctions between MXene and another material are possible with the aid of in situ manufacturing procedures. The 2D MXene NM has also been used to produce metal oxide^[^ ^378^ ^]^ and carbon,^[^ ^379^ ^]^ with potential applications for the chalcogenides group.^[^ ^380^ ^]^ Even though this discovery has a lot of promise, further theoretical and experimental analysis needed to be done. For instance, it is important to carefully consider customized metal cleaving or accurate metal conversions inside single metal or ordered double‐metal 2D MXene NMs, correspondingly. Additionally, core–shells/hollow structures, hybrid composites, derivation to metal chalcogenides, hierarchical structure formation (0D to 3D), flexible and conductive current collectors, and transformation to novel MXene materials with special properties may cater to advance applications that may all serve as inspirations.

While progress was attained in the production of TMCs based 2D MXene NMs and in 2D TMNs and non‐TI MXene still has some limitations. To integrate 2D MXene NMs on chips using current microfabrication device nanotechnologies, vapor phase production is essential. Generally non‐Ti based 2D MXene NMs will be used in future additive manufacturing techniques based nanotechnologies if large‐scale, environmentally friendly manufacturing techniques are introduced. Defined control configuration and surface chemistry, including defects and strain engineering, should cover the approach for theoretically anticipated intrinsically semiconducting, TIs, and ferromagnetic 2D non‐Ti MXene NMs, and additional their physics and chemistry breakthroughs. The non‐Ti MXene NMs that are mechanically robust, somehow environmentally stable, and highly conductive may have an important influence on flexible, printable, and wearable self‐powered electronics. The use of MXene NMs, especially non‐Ti MXene NMs in conjunction with other 2D NMs to self‐assemble heterostructures and electronics is very interesting idea. Overall, the exploration of non‐Ti MXenes offers an opportunity for the discovery of new materials with unique properties and potential applications, as well as the development of more sustainable materials.

## Conflict of Interest

The authors declare no conflict of interest.
